# Survival and divergence of Arctic endemic cod species during 50,000 years of climate change

**DOI:** 10.1038/s41467-026-78810-4

**Published:** 2026-10-16

**Authors:** Julek Chawarski, Flor Vermassen, Kim Vane, Hauke Flores, Jutta Wollenburg, Hendrik Grotheer, Claudia Morys, Nicole Hildebrandt, Helen K. Coxall, Pauline Snoeijs-Leijonmalm

**Affiliations:** 1 Department of Ecology, Environment and Plant Sciences, Stockholm University, Stockholm, Sweden; 2 Department of Geological Sciences, Stockholm University, Stockholm, Sweden; 3 Bolin Centre for Climate Research, Stockholm University, Stockholm, Sweden; 4 Alfred Wegener Institute Helmholtz Centre for Polar and Marine Research, Bremerhaven, Germany; 5 Department of Geosciences and MARUM Centre for Marine Environmental Sciences, University of Bremen, Bremen, Germany; 6 Present Address: ASL Environmental Sciences, Victoria, British Columbia Canada; 7 Present Address: AquaEcology GmbH & Co. KG, Oldenburg, Germany

**Keywords:** Evolutionary ecology, Palaeoecology

## Abstract

Despite significant climate variability in the Northern Hemisphere ~50,000–12,000 years ago, the potential for vertebrates to periodically thrive in the glacial Arctic Ocean has been largely overlooked because ecosystem productivity was assumed to be too low. Here, we analyze fish otoliths preserved in deep-sea sediments and find that two Arctic endemic cod species, ice cod (*Arctogadus glacialis*) and polar cod (*Boreogadus saida*), lived at 87–89 °N ~ 48,000–35,000 years ago and reappeared at the onset of the Holocene ~12,000 years ago. *A. glacialis* also occurred north of Greenland at 84–85 °N ~ 20,000 years ago, during the peak of the Last Glacial Maximum, suggesting the presence of refugia that enabled survival and divergence from *B. saida* under the harshest climate conditions. These findings suggest that the glacial Arctic Ocean was not a barren ice desert but rather a breeding ground for the evolution of cold adaptation in marine fish.

## Abstract (web summary)

The high Arctic Ocean is generally considered too unproductive to support persistent vertebrate populations. Here, an analysis of ancient fish ear bones shows that endemic cod species inhabited the region over the past 50,000 years, including the Last Glacial Maximum.

## Introduction

Whether vertebrate life existed beneath the ice cover of the Earth’s Arctic Ocean during past ice ages remains unknown. It has been suggested that the Arctic deep basins and ridges north of 84 °N (here referred to as the “Central Arctic Polar Domain”) were covered by thick perennial ice during the second half of the last ice age ~50–12 thousand years before present (ka BP), allowing for almost no biological production^1–3^. However, the Northern Hemisphere climate during this period was highly variable, with episodes of extreme glacial expansion culminating in the Last Glacial Maximum (LGM) ~ 27–19 ka BP, interspersed with brief periods of relative warming^4–6^. Temporary reductions in sea-ice extent and volume, combined with increased advection of Atlantic and Pacific water masses and elevated sedimentation rates, suggest that intermittent periods of slightly increased productivity may have occurred even at the highest latitudes^7–9^.

This raises the question of whether the biological productivity during warmer episodes over the past 50,000 years was sufficient to support complex food webs, including vertebrates such as fish and their predators (seabirds, seals, polar bears), in the Central Arctic Polar Domain. Current annual net primary production in the area is about 1 g C m^−2^ year^−1^^10^, roughly two orders of magnitude lower than that in the more nutrient-rich Arctic shelf seas (such as the Barents, Kara, and Chukchi Seas) and in the adjacent North Atlantic and North Pacific Oceans. Despite the low productivity in the Central Arctic Polar Domain, two cryophilic gadoid (cod) species are present today: the Arctic endemics ice cod (*Arctogadus glacialis*) and polar cod (*Boreogadus saida*). *B. saida* is the most abundant fish species in the Central Arctic Polar Domain and adjacent shelf seas, whereas *A. glacialis* has a circumpolar distribution but has not been recorded north of ~84 °N^11,12^ and appears to be most abundant along the northeast coast of Greenland^13^. These two Arctic gadoids are key links between the lower and higher trophic levels of the current Arctic food web^14,15^. However, despite their cold adaptations, such as antifreeze proteins that enable them to thrive in water temperatures below 0 °C^16^, it remains unclear whether these fish could have endured the extreme conditions of the last ice age, when ecosystem productivity in the Central Arctic Polar Domain was likely even lower than it is today.

In this study, we analyzed otoliths (ear stones) from the heads of bony fishes collected from deep-sea sediments in the Arctic Ocean. Otoliths have species-specific morphology, and those preserved in sediments provide a valuable archive of past species occurrences spanning thousands to millions of years^17,18^. We demonstrate that both *A. glacialis* and *B. saida* could sustain populations at 87–89 °N in the glacial CAO during the last ice age, but not under the harshest climatic conditions of the Last Glacial Maximum. *A. glacialis* (but not *B. saida*) was able to survive the peak of the Last Glacial Maximum in refugia at 84–85 °N, suggesting a mechanism of climate-driven divergence between these two Arctic endemic cod species.

## Results and Discussion

### Gadoid otoliths and climate over the past 50,000 years

**Fig. 1:**
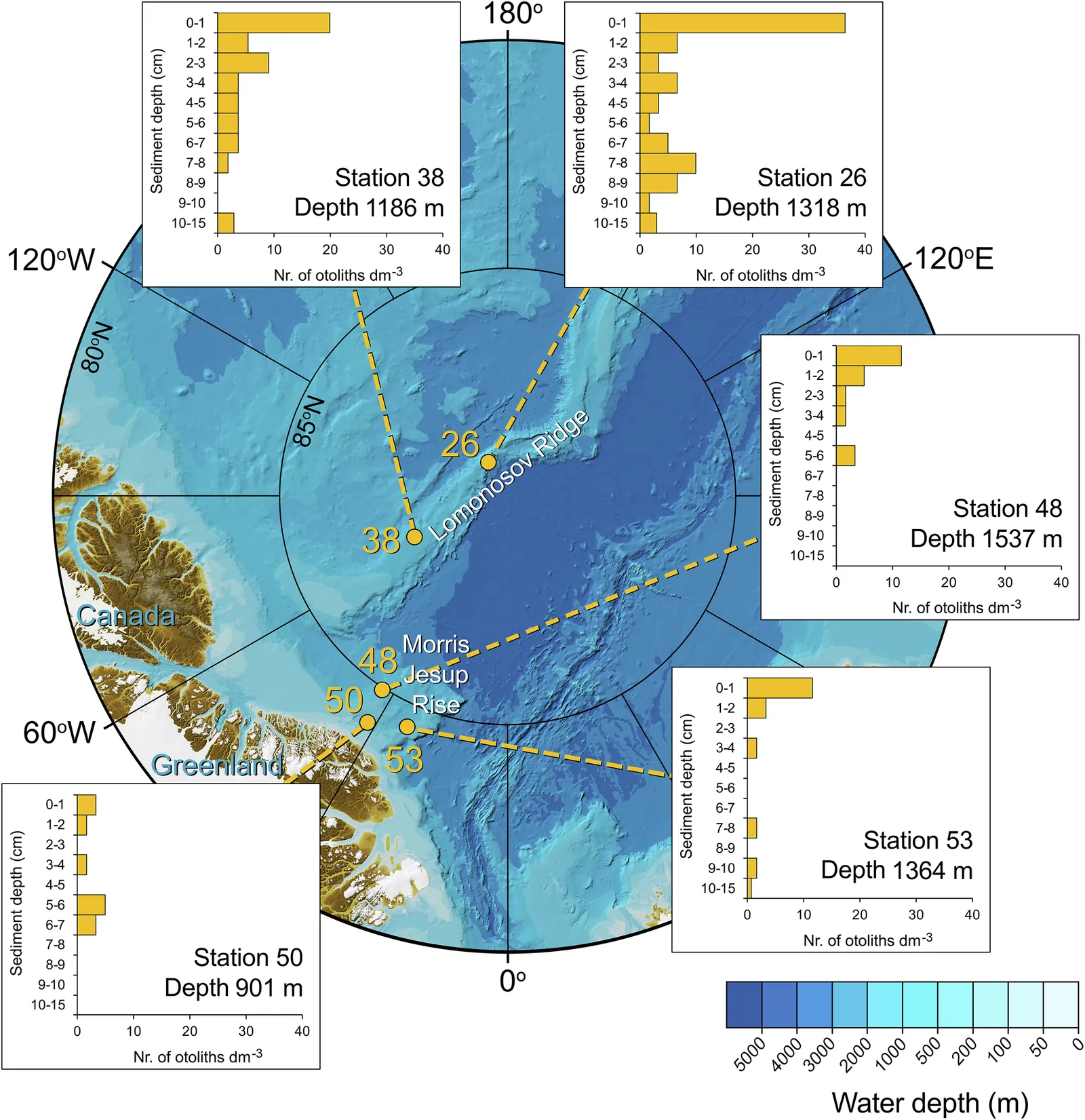
Map showing the locations of the five sampling stations and the downcore otolith abundances at each station. The otolith abundances are based on all 131 otoliths (across all species) found in the stratified sediment samples from push cores (Supplementary Data 2). The 125 otoliths found in the non-stratified bulk samples are not included in this figure. Stations 26 and 38 are located on the western Lomonosov Ridge (Central Arctic Ocean), while Stations 48, 50, and 53 are on the Morris Jesup Rise, which extends into the Eurasian Basin from the Northeast Greenland Shelf. Otolith abundance is expressed as the number of otoliths per volume of wet sediment within each stratum. The background map is based on the Bathymetric Map of the Arctic Ocean (IBCAO) Version 5^61^. Source data are provided as a Source Data file.

We analyzed 256 otoliths collected from deep-sea sediments at water depths ranging from 901 to 1537 m across five sampling stations between 84 °N and 89 °N (Fig. 1; Supplementary Data 1). Otoliths were scarce in the sediment samples, with average densities ranging from 1.0 (Station 50) to 6.4 (Station 26) otoliths per cubic dm of sediment (Supplementary Data 2). Other fish remains, such as teeth or vertebrae, were not immediately apparent in the sieved sediments. The highest otolith densities were observed on the western Lomonosov Ridge in the Central Arctic Ocean (CAO)^19^, and the lowest on the Morris Jesup Rise, which extends into the Eurasian Basin from the Northeast Greenland Shelf outside the CAO (Fig. 1). At four of the five sampling stations, the top 0–1 cm sediment layer had the highest otolith density.

**Fig. 2:**
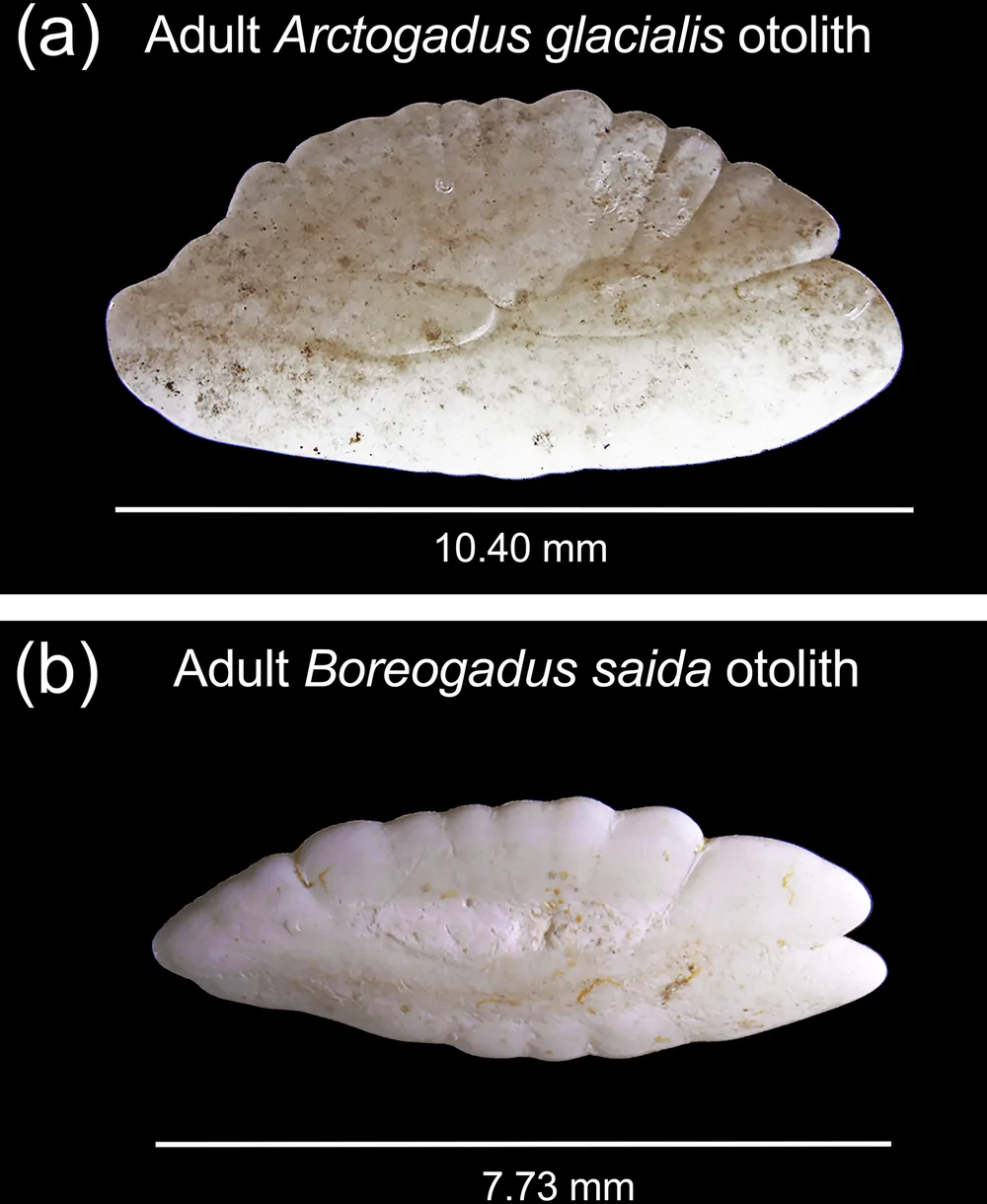
Light microscopy images of otoliths from two gadoid species found in deep-sea sediments on the western Lomonosov Ridge. **a** Ice cod (*Arctogadus glacialis*) medial face, otolith SO21_BC5_BULK_OTObc327, radiocarbon age 20,353 ± 289 years (calibrated mean age ± standard deviation). **b** Polar cod (*Boreogadus saida*) medial face, otolith SO21_BC5_BULK_OTObc345, radiocarbon age 11,310 ± 285 years. Adult *B. saida* otoliths have more crenulations (notches) around the entire margin than *A. glacialis* and are generally more elongated than the more elliptical otoliths of *A. glacialis*, although there is considerable shape overlap (Supplementary Figs 5 and 6). The morphological differences are generally clear and consistent across otoliths from both species. For further details on gadoid species identification, see Supplementary Figs 1 and 3.

Two hundred and six otoliths (80%) could be identified to the species level (Supplementary Figs 1–3), but for 50 otoliths (20%), this was not possible because their distinguishing features were insufficient due to small size and/or preservation state (Supplementary Data 3). The identified otoliths originated from the two Arctic endemic cod species, ice cod (*A. glacialis*; 57 otoliths, length 2.8–14.2 mm) and polar cod (*B. saida;* 145 otoliths, length 2.2–8.3 mm) (Fig. 2, Supplementary Fig. 1), as well as from snailfishes (*Paraliparis* sp.; 4 otoliths 1.2–2.0 mm in length)^20^, (Supplementary Fig. 2).

Radiocarbon dating of a subset of 79 otoliths showed that the calibrated ages ranged from 1,3 ± 0.2 ka BP (mean ± standard deviation) to 47,8 ± 2,2 ka BP (Supplementary Data 4). Thirty-seven otoliths were dated to 11.3–1.3 ka BP (Holocene), 13 to 20.3–18.6 ka BP (LGM), and 29 to 47.8–29.1 ka BP (last ice age before the LGM). Otoliths consist of precipitated calcium carbonate in the form of aragonite or vaterite, which are more prone to dissolution than calcite^21^. In some cases, calcite appears absent during recent interglacials at locations with high labile organic matter deposition^22,23^, due to long-term pore water dissolution^24^ or a shallowed glacial lysocline^25^. Although this might suggest vulnerability of our sedimentary otoliths to dissolution, extremely low sedimentation rates and foraminiferal abundances during the LGM^26,27^ would suggest that low biological productivity is the main reason for the low otolith abundances rather than dissolution.

**Fig. 3:**
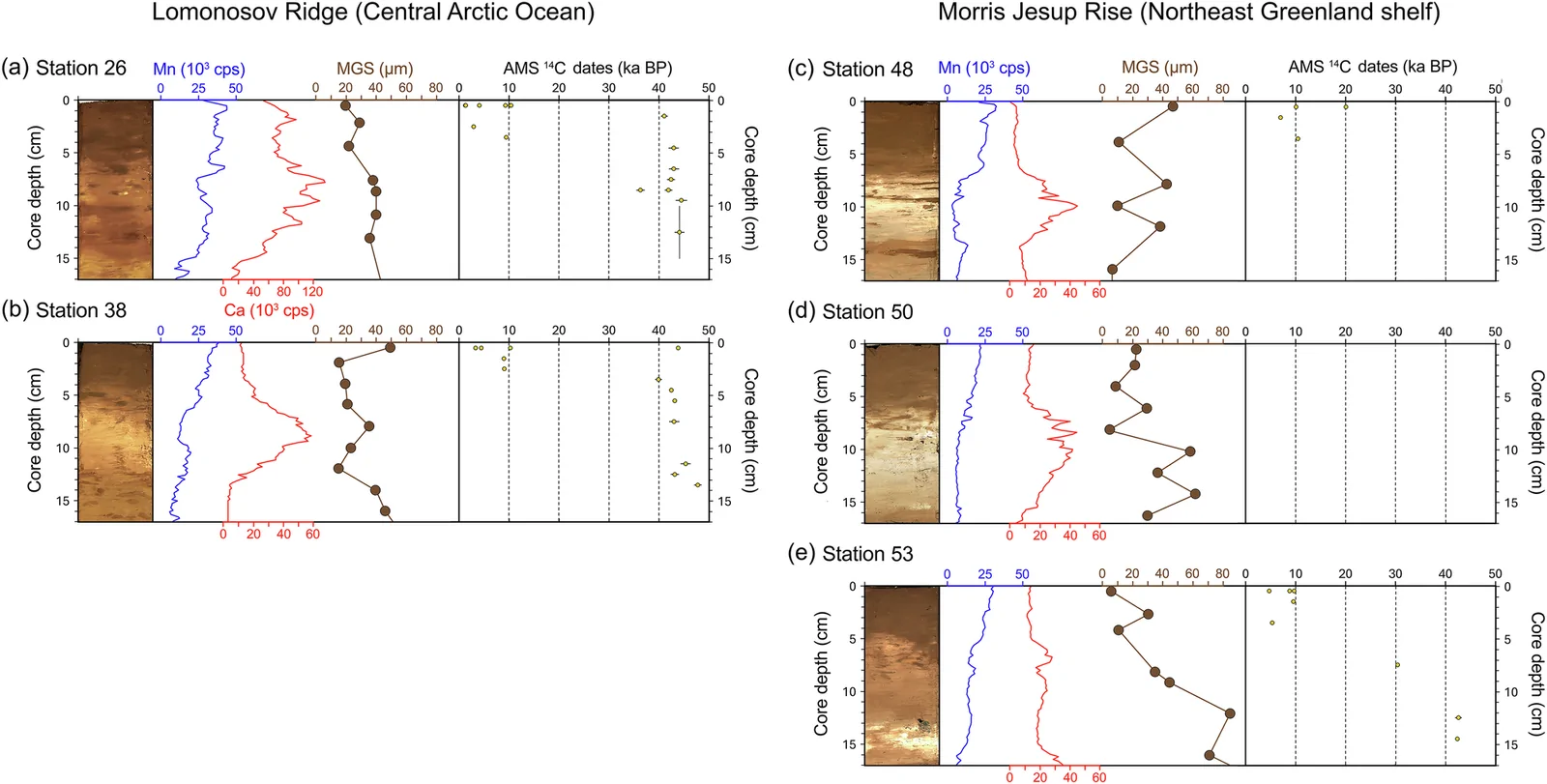
Accelerator Mass Spectrometry (AMS) ^14^C dates of otoliths in relation to sediment properties at the five sampling stations. This figure is based on 43 radiocarbon-dated otoliths from stratified push-core samples. The 36 radiocarbon-dated otoliths from non-stratified bulk samples are not included. **a** Station 26, western Lomonosov Ridge. **b** Station 38, western Lomonosov Ridge. **c** Station 48, Morris Jesup Rise. **d** Station 50, Morris Jesup Rise. **e** Station 53, Morris Jesup Rise. More otoliths suitable for radiocarbon dating were available in stratified samples from the western Lomonosov Ridge than from the Morris Jesup Rise. Photographs of split push core sections show sediment lithology, with the darker-brown Holocene layer at the upper 0 to ~5 cm and the lighter-colored glacial layers below. Otoliths younger than 12,000 years (Holocene) were generally found in the Holocene layer, and otoliths older than 35,000 years in the lower 5–15 cm glacial layers. Sediment mixing (bioturbation) likely occurred within each layer. Sediment chemical and physical properties include manganese content (Mn), calcium content (Ca), and mean grain size (MGS). The vertical error bar in (**a**) indicates that this otolith was sampled within the 10–15 cm interval, while the other 42 otoliths were sampled at 1 cm core depth intervals. Horizontal error bars represent 1 standard deviation for the mean calibrated accelerator mass spectrometry (AMS) radiocarbon dates. When no horizontal error bar is visible, the error bars fall within the yellow symbol, illustrating higher radiocarbon dating uncertainty for otoliths older than 30 ka BP. cps, counts per second; ka BP, thousand years before present. Source data are provided as a Source Data file.

Since our interpretations are based on direct otolith dating (without constructing age-depth models for the cores), the absence of LGM otoliths in the CAO does not stem from a stratigraphic gap lacking otoliths (Fig. 3). The modern aragonite lysocline occurs at approximately 3,500 m of ocean depth^28^, well below the depths of our sampling stations (901–1,537 m). Although the lysocline depth likely shallowed during glacial stages^25^, the co-occurrence of our MIS3-aged otoliths with calcareous microfossils and (aragonitic) pteropod shells suggests that aragonite dissolution bias is unlikely. Otolith fragments (*A. glacialis* and *B. saida*) from our sediment samples analyzed with Scanning Electron Microscopy (SEM) / Energy Dispersive Spectroscopy (EDS), show no difference in structure or chemical composition from recent *B. saida* otoliths, except that recent otoliths have an organic surface coating (rich in C and N) that is missing in the sediment otoliths (Supplementary Fig. 7).

There was no linear correlation between sediment depth and the radiocarbon age of the otoliths. Instead, two distinct layers indicated two separate time periods: otoliths younger than 12,000 years (Holocene) were found in the darker-brown top 0 to ~0–5 cm sediment layer, and otoliths older than 35,000 years were found in the lighter-colored (calcium-enriched) lower ~5–15 cm sediment layer (Fig. 3). Exceptions to this pattern included two otoliths older than 40,000 years recovered from the top 0–2 cm of the Lomonosov Ridge cores, which may have been displaced by mixing between the two layers. Apart from these exceptions, our data suggest that sediment mixing likely occurred within each layer (Fig. 3).

These observations could be explained by changes in sedimentation and bioturbation rates over time. One possibility is that sedimentation rates during the second half of the last ice age, but before the LGM, were relatively high for a short period^29^, which could explain the apparent clustering and the larger sediment layer interval (5–15 cm) containing otoliths older than 35,000 years. Subsequently, as the climate cooled toward the peak of the LGM, primary productivity, sedimentation rates, and bioturbation were essentially halted in the CAO beneath very thick perennial ice, as previously suggested^1,3^. The LGM period would thus be a very condensed horizon ( < 1 cm) in our records, or possibly a hiatus^29,30^, corresponding to the lithological boundary observed around 5 cm of sediment depth (Fig. 3). Thereafter, sedimentation rates likely returned to the typical, relatively low interglacial levels, accompanied by bioturbation in the top 0–5 cm of sediment^27,31^, thus mixing Holocene-aged otoliths within the Holocene layer.

**Fig. 4:**
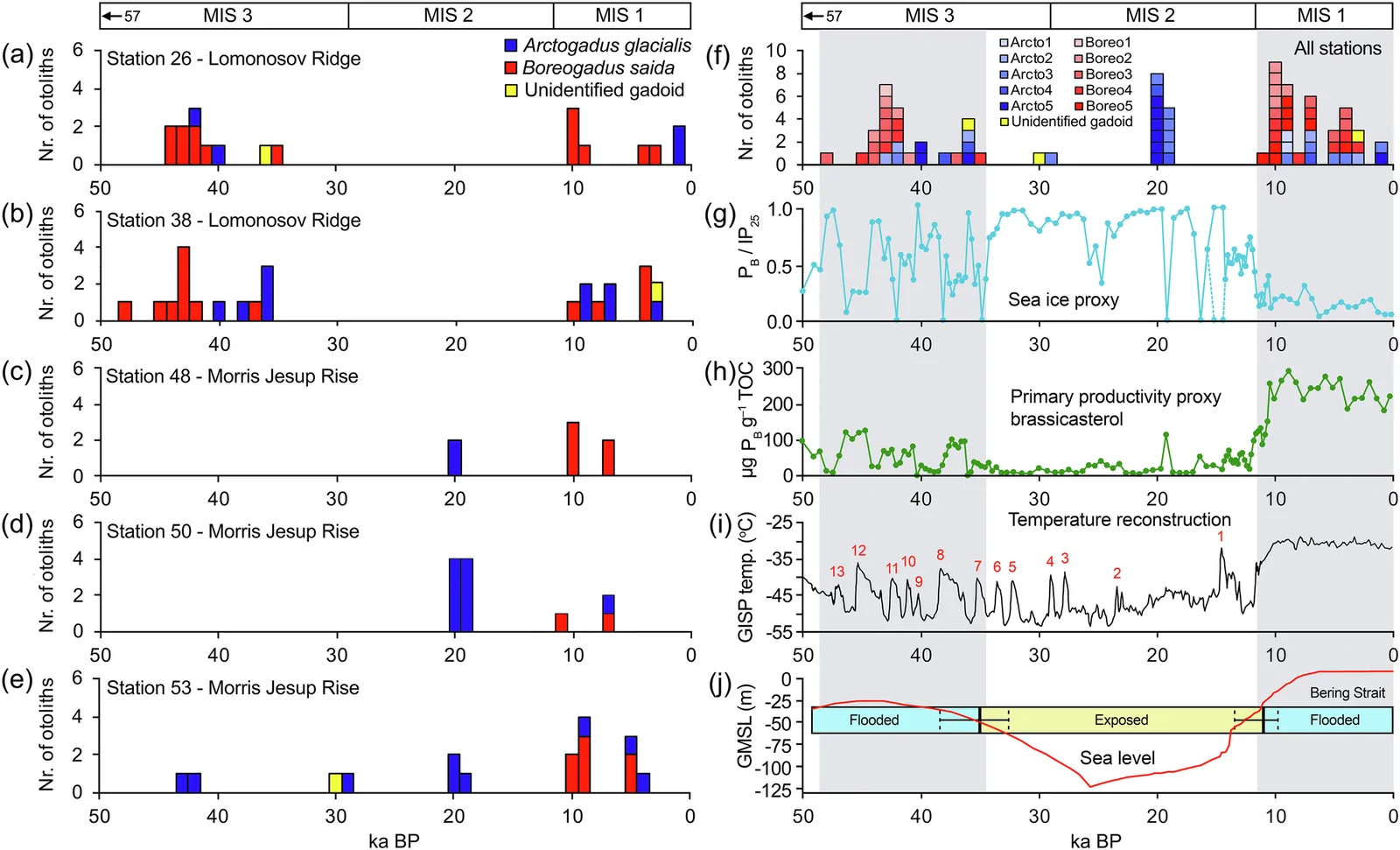
Occurrences of Arctic gadoid otoliths in sediment samples, in relation to oceanic and climate proxies in the North Atlantic and North Pacific Oceans, over the past 50,000 years. This figure is based on all 79 radiocarbon-dated otoliths, 43 from stratified and 36 from bulk sediment samples (34 *Arctogadus glacialis*, 42 *Boreogadus saida*, and three unidentified gadoids). **a** Station 26 (n = 19 radiocarbon-dated otoliths). **b** Station 38 (n = 25). **c** Station 48 (n = 7). **d** Station 50 (n = 11). **e** Station 53 (n = 17). **f** All stations combined, showing identification confidence scores 1-5 (Supplementary Fig. 3) for *A. glacialis* and *B. saida*, illustrating that erosion and damage of the otolith surfaces were evident throughout the entire study period. **g** Sea ice index P_B_/IP_25_, the ratio (g/g) of the phytoplankton-derived sterol lipid brassicasterol (P_B_) and the isoprenoid lipid (IP_25_), which is mainly synthesized by diatoms; the data were derived from sediment samples collected in the North Atlantic between Iceland and Norway, redrawn from Fig. 5 in Hoff et al. (2016)^36^. **h** Proxy for surface primary productivity, expressed as micrograms of brassicasterol per gram of total organic carbon (TOC), measured from sediment samples collected in the North Atlantic between Iceland and Norway, redrawn from Fig. 5 in Hoff et al. (2016)^36^. **i** Temperature reconstruction based on an ice core from the Greenland Ice Sheet Project (GISP), featuring Dansgaard-Oeschger (D-O) events (abrupt periods of warming during the last ice age), numbered 1–13, redrawn from Fig. 3 in Cuffey & Claw (1997)^34^. **j** Global Mean Sea Level (GMSL) reconstruction and timing of open and closed water exchange between the Arctic Ocean and the North Pacific through the Bering Strait, sea level redrawn from Fig. 4 in Farmer et al. (2023)^9^. MIS = Marine Isotope Stage; MIS 3, a glacial period before the LGM; MIS 2, the Last Glacial Maximum (LGM); MIS 1, the Holocene; ka BP, thousand years before present. The period between the gray bars (35–12 ka BP) in (**f–j**) marks the intermittent period of maximum glaciation (**g**) and lower productivity (**h**), coinciding with a closed Bering Strait (**j**). Source data are provided as a Source Data file.

The geological record covered by our study encompasses three of the global Marine Isotope Stages (MIS)^32^. Our otolith dataset includes much of MIS 3 ( ~ 57–29 ka BP), MIS 2 ( ~ 29–12 ka BP), and MIS 1 (Holocene, since ~12 ka BP) (Fig. 4a–f). On the western Lomonosov Ridge, the earliest period with otolith occurrences of *A. glacialis* and *B. saida* spanned MIS 3 from about 48 to 35 ka BP. Overall, MIS 3 was characterized by climate instability and abrupt global warming periods, known as “Dansgaard-Oeschger (D-O) events”^33,34^ (Fig. 4i), providing a plausible mechanism for short-lived improvements in environmental conditions in the CAO. However, the general uncertainty in radiocarbon dating beyond 30 ka BP (Supplementary Data 4) limits the ability to match individual otolith peaks with specific D-O events.

### CAO fish survival depends on Atlantic and Pacific inflows

Our otolith records from 87–89 °N in the CAO (western Lomonosov Ridge) during MIS 3 (Fig. 4a, b) likely reflect millennial-scale pulses of Atlantic and Pacific water entering the Arctic basins during D-O events, reducing marine glacial conditions and causing periods of reduced sea-ice extent and increased biological productivity sufficient to support vertebrate life. Independent geological data from the broader Arctic region support intermittently milder conditions during MIS 3. For example, rapid climatic shifts during MIS 2–4 were documented by analyses of planktic foraminifers in Fram Strait^35^. Further south, sediment records from the Norwegian Sea during this period show minima in the sea-ice proxy P_B_/IP_25_ (Fig. 4g)^36^ and maxima in the phytoplankton-derived biomarker brassicasterol (Fig. 4h)^36^, indicating periods of open water, Atlantic water inflow, and increased surface productivity in the Nordic Seas during D-O events, which interrupted the otherwise extensive glacial sea-ice conditions in the region (Fig. 4i)^34^.

Furthermore, the Northeast Greenland Ice Stream retreated beyond its current extent^37^, the Laurentide Ice Sheet underwent a marked reduction^38^, and central Scandinavia was likely ice-free from approximately 55 ka BP to 35 ka BP^39^. Coinciding with the instability of the Laurentide Ice Sheet, warming during the initial phase of D-O events 8–12 (45–35 ka BP; Fig. 4i)^34^ caused sufficient sea-ice thinning to allow pulses of primary and secondary production along ridges in the CAO^40^. Additionally, sea-level rise from melting ice sheets during MIS 3 submerged the glacial land bridge across the Bering Strait (Fig. 4j)^9^, allowing nutrient-rich Pacific waters to flow in and further boost biological productivity (Fig. 4h)^36^. Collectively, these conditions suggest that biological productivity in the CAO during MIS 3 was probably – at least periodically – sufficient to support populations of gadoid fishes, potentially reaching levels similar to their modern abundances and distributions.

By around 35 ka BP, the climate began to cool toward the LGM (Fig. 4g)^36^. Extreme glaciation caused global sea levels to drop by more than 100 m, exposing large areas of the Arctic continental shelves, and re-establishing a land bridge across the Bering Strait (Fig. 4j)^9^. Advection of nutrients and plankton to the CAO sharply declined as the Pacific inflow ceased and the Atlantic inflow decreased^7,8^. Additionally, land-derived particle-associated organic matter decreased in the absence of the Transpolar Drift and Beaufort Gyre, and organic matter was mainly retained near the edges of the Arctic deep basins^41,42^.

Consistent with these environmental changes, our sedimentary otolith records from the western Lomonosov Ridge (Stations 26 and 38, separated by hundreds of kilometers) show an approximately 25,000-year absence of *A. glacialis* and *B. saida* otoliths toward the end of MIS 3 and throughout MIS 2 (Fig. 4a, b). Of the 44 radiocarbon-dated otoliths from these two stations, 25 are older than 35,000 years, 19 are younger than 12,000 years, and none are from the period between 12 and 35 ka BP. These data suggest that, during the LGM, biological productivity in the CAO fell below levels needed to sustain significant gadoid fish populations, coinciding with thick perennial sea ice across much of the deep basins and with ice shelves potentially extending hundreds of kilometers from the coasts^43^. This interpretation aligns with other proxies indicating extensive perennial ice and near-zero biological productivity in the CAO at that time^2,40^. The Greenland, Laurentide, and Fennoscandian ice sheets reached their maximum extents in the past 50,000 years during the LGM^44^, and even the Nordic Seas were largely ice-covered, with low net primary productivity (Fig. 4g, h)^36,39^.

After about 25,000 years of absence, otoliths of *A. glacialis* and *B. saida* reappeared on the western Lomonosov Ridge at the onset of the Holocene ( ~ 12 ka BP) (Fig. 4a, b). A reduction in global ice sheet volume led to approximately 100 m of sea-level rise, reopening the Bering Strait (Fig. 4j)^9^ and increasing inflow to the CAO through both the Atlantic and Pacific gateways. The reappearance of the two Arctic endemic gadoids was likely driven by increased nutrient advection from the Atlantic and Pacific Oceans, along with thinner sea ice that enhanced biological productivity. Global proxy record compilations indicate that the first half of MIS 1, which roughly coincides with the early Holocene Climatic Optimum, was relatively warm, with global average surface temperatures about 0.7 °C higher than during the nineteenth century^45^. Maximum otolith occurrence during MIS 1 (Fig. 4f) coincided with the Holocene Climatic Optimum in the western Arctic ( ~ 11–9 ka BP)^46^. Ice sheet retreat and the subsequent establishment of consistent summer ice-free conditions during the Holocene Climatic Optimum enabled a post-glacial range expansion for many pelagic marine species and likely facilitated the arrival of Arctic fish predators, including seabirds, seals, and whales. After the Holocene Climatic Optimum, gadoid otoliths in the sediments decreased to present-day levels (Fig. 4f). The youngest (1,276 to 1,415 years old) *A. glacialis* otoliths found in the CAO, located in the upper 0–1 cm sediment layer, could indicate that this species currently has a broader distribution in the CAO than assumed^12^.

### Climate-driven divergence between Arctic endemic cod species

Our otolith records from 84–85 °N on the Morris Jesup Rise show sporadic, low occurrences of *A. glacialis* otoliths and the absence of *B. saida* otoliths during the glacial periods MIS 3 and MIS 2 (Fig. 4c–e). Genomic studies suggest that these two species experienced cycles of co-occurrence and isolation during the last ice age^47,48^, possibly reflecting periodic absences and recolonizations of *B. saida* in the Central Arctic Polar Domain^49^. The absence of *B. saida* from the Morris Jesup Rise and the Lomonosov Ridge during MIS 2, along with the presence of only *A. glacialis* at the Morris Jesup Rise, aligns well with previous hypotheses about their climate-change adaptation strategies: *A. glacialis* likely persisted in a localized refuge, experiencing a population bottleneck and reduced genetic diversity, whereas *B. saida* appears to have retreated to lower latitudes, maintaining higher genetic variation through range expansion^47^.

A distinct period of increased *A. glacialis* otolith presence occurred in the middle of the LGM over a narrow span of about 1,700 years (20.3–18.6 ka BP) (Fig. 4c–e). This period coincides with the formation of open-water areas surrounded by sea ice (polynyas) along the northwestern margin of the Eurasian ice sheet around 20–19 ka BP, following the collapse of the Svalbard-Barents ice sheet^50^. Modern polynyas form through the combined effects of katabatic winds blowing seaward from ice shelves and the upwelling of warmer, subsurface Atlantic water. Similar conditions during this short period may have created “oases” of biological productivity in the glacial Arctic north of Greenland. A brief but pronounced decline in sea-ice cover, accompanied by a sudden increase in net primary productivity in the North Atlantic, as indicated by the proxies P_B_/IP_25_ and brassicasterol (Fig. 4g, h)^36^, further supports the presence of temporarily milder conditions in the Nordic Seas. Enhanced Atlantic water circulation likely played a crucial role in maintaining glacial oases on the Morris Jesup Rise during MIS 3 and MIS 2. During the early Holocene, sea-ice reconstructions and modeling indicate a transition from perennial to seasonal sea ice north of Greenland during the Holocene Climatic Optimum^51,52^. Consistent with this, otolith records from the Morris Jesup Rise indicate that *A. glacialis* and *B. saida* coexisted there during the Holocene, mirroring the pattern observed on the western Lomonosov Ridge (Fig. 4a–e).

**Fig. 5:**
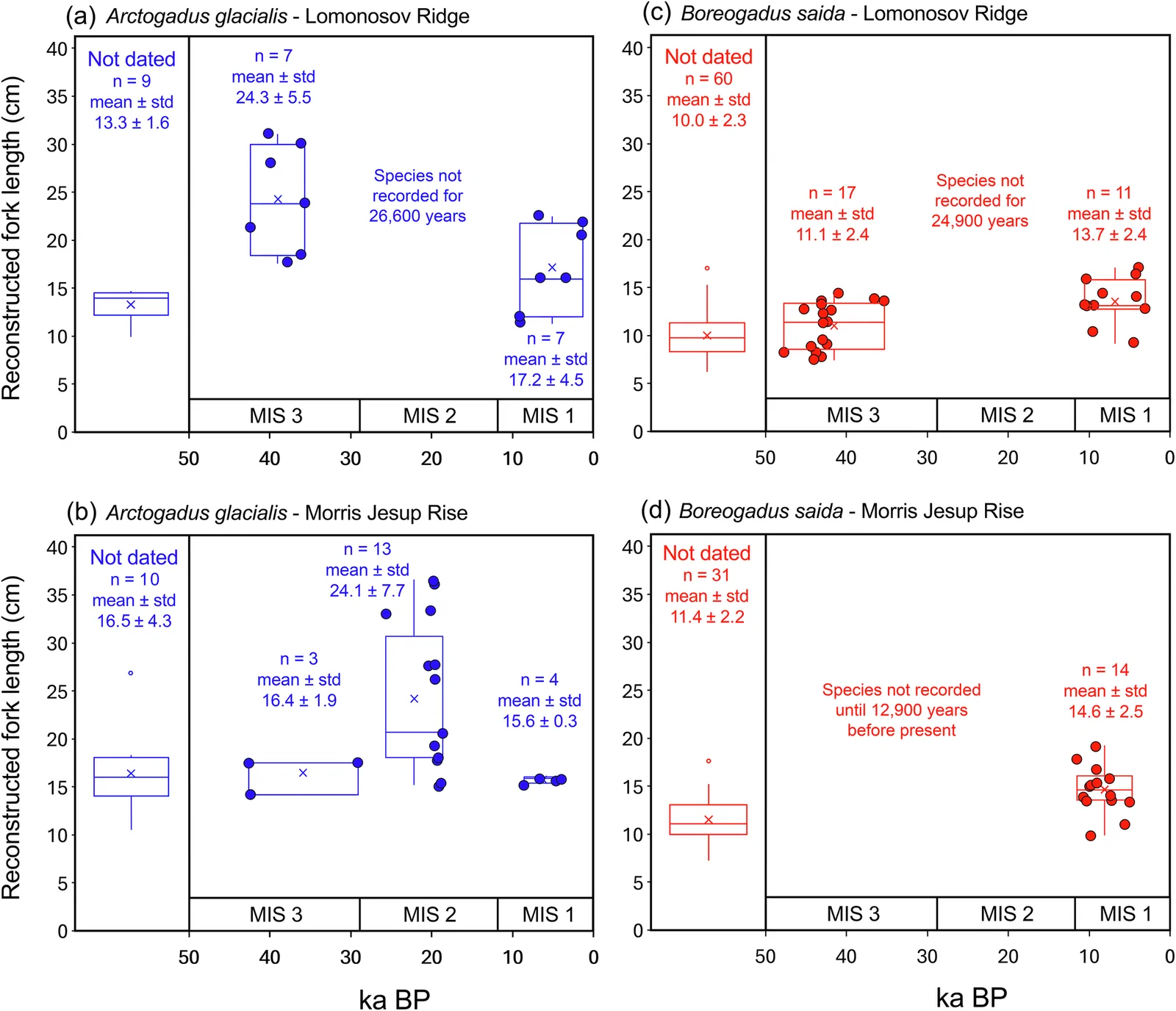
Box and whisker plot comparison of reconstructed fish body size (fork length) between *Arctogadus glacialis* and *Boreogadus saida* for the western Lomonosov Ridge and the Morris Jesup Rise, shown separately. This figure is based on 186 morphologically intact otoliths, which enabled both fork-length reconstruction and species identification. Of these 186 otoliths, 76 were radiocarbon-dated (34 *A. glacialis*, 42 *B. saida*) and 110 were not radiocarbon-dated (19 *A. glacialis*, 91 *B. saida*). **a** *A. glacialis* from the western Lomonosov Ridge (Stations 26 and 38). **b** *A. glacialis* from the Morris Jesup Rise (Stations 48, 50, and 53). **c** *B. saida* from the western Lomonosov Ridge. **d** *B. saida* from the Morris Jesup Rise. The non-radiocarbon-dated otoliths indicate that nearly all large otoliths were selected for radiocarbon dating, supporting the finding that the largest *A. glacialis* otoliths in the dataset were found during MIS 3 on the western Lomonosov Ridge and during MIS 2 on the Morris Jesup Rise. The Box and Whisker plots display the median fork length (middle line), the upper and lower quartiles, and for the dated otoliths the radiocarbon age range (box width), the minimum and maximum fork lengths (whiskers), the mean value (x), and outliers (o). Fork length is measured from the tip of the snout to the fork of the tail and was calculated using published allometric relationships between otolith size and body size for each species^72^. MIS Marine Isotope Stage, MIS 3, glacial period before the LGM, MIS 2, Last Glacial Maximum (LGM), MIS 1, Holocene, ka BP, thousand years before present. Source data are provided as a Source Data file.

Differences in the size distributions of *A. glacialis* otoliths across marine isotope stages indicate shifts in ecological conditions. Otolith-based size reconstructions show that the mean size of *A. glacialis* was larger during MIS 3 than during MIS 1 at the Lomonosov Ridge (Fig. 5a). At the Morris Jesup Rise, both mean size and size diversity of *A. glacialis* were higher during a period of increased occurrence during MIS 2 than during MIS 3 and MIS 1 (Fig. 4c–e; Fig. 5b). In contrast, no significant changes in size distributions between MIS stages or regions were observed for *B. saida* (Fig. 5c–d). Together with the absence of *B. saida* from the Morris Jesup Rise during MIS 3 and MIS 2, these patterns suggest that changes in habitat structure contributed to the subtle divergence in the life histories of the two species we see today^14^.

Our findings demonstrate that *A. glacialis* and *B. saida* are adapted to endure planetary ice ages, which were the dominant states of Earth’s climate during their evolutionary history^49,53^. These two species are sister taxa with a more recent common evolutionary origin than other gadoid species^49,54^. *A. glacialis* and *B. saida* are thought to have diverged between 5 and 10 million years ago^16,54^, and have likely undergone allopatric speciation after establishing reproductive isolation, with little to no ongoing gene flow^55^. While remaining sympatric during mild interstadial and interglacial conditions, climate-driven divergence between the two species, such as during the LGM in our study, highlights a potentially important aspect of their speciation process.

### Anthropogenic global warming and Arctic endemic fish

Contemporary observations show that *A. glacialis* thrives in glacier-proximal waters of northeast Greenland, including deep fjords^13,14^ and the Northeast Water Polynya^56^, suggesting that *A. glacialis* is an ice-dependent glacial-edge specialist. Its low intraspecific genetic variability^47^ may constitute a bottleneck during warmer periods. Hence, *A. glacialis* is probably the Arctic endemic gadoid most vulnerable to the ongoing anthropogenic global warming. *B. saida* is also cold-adapted^16^, but it might have a greater capacity to adapt to changing environmental conditions because of high genetic variation^57^. Interspecific differences in tolerance to ice-free conditions are also reflected in current distribution patterns. *A. glacialis* is more strictly Arctic within its current southernmost Atlantic distribution limit at the Greenland coast around 60 °N, whereas *B. saida* can be found as far south as the Gulf of St. Lawrence at 46 °N^12^.

The results of this study demonstrate that extreme cold conditions during the last ice age did not create a permanently frozen desert across the Central Arctic Polar Domain. Earlier depictions of ice-age ecosystems as lacking higher-trophic marine life are insufficiently supported by the limited paleontological and phylogeographic evidence. Instead, millennial-scale climate variability drove local environmental changes that could periodically support Arctic fish populations and, potentially, a complex food web, including seabirds and marine mammals, similar to the one observed today.

In 2024, the global average temperature exceeded 1.5 °C above the 19^th^-century level for the first time in human history, roughly doubling the warming during the Holocene Climatic Optimum^45,58^. Even the sea ice covering the deep basins and submarine ridges of the CAO is becoming progressively thinner, younger, and more dynamic, while midwater temperatures continue to rise^59^. Further anthropogenic climate warming is likely to soon exceed the thermal tolerance of the Arctic endemics *A. glacialis* and *B. saida*, which have inhabited the unique permanently ice-covered marine ecosystem of the CAO for the past 50,000 years. Northward expansion of boreal species will increase competition and predation in the Arctic pelagic system, putting these two cryophilic cod species at significant risk of population declines.

## Methods

### Ethical statement

The scientific research conducted during the 2021 Swedish Synoptic Arctic Survey (SAS) expedition on the Morris Jesup Rise within the Greenland Exclusive Economic Zone, including destructive sampling of fish otoliths found in sediments, complies with all relevant ethical regulations and national guidelines with permission from the Danish Ministry of Foreign Affairs, Copenhagen (JTHAV Nr. 2021-27049, August, 19^th^, 2021). No permission was necessary for research conducted in the high seas of the Central Arctic Ocean, as this area is outside national jurisdiction.

### Sediment sampling and otolith collection

Field sampling was conducted during the Synoptic Arctic Survey (SAS) expedition aboard the Swedish icebreaker *Oden* from August 19 to September 6, 2021^60^. Suitable areas with stratified sediments were identified using ship-based acoustic sub-bottom profiling^61^. Box-core sediment samples were collected with a “Giant Box Corer” covering 50×50 cm^2^ and reaching a maximum depth of about 40 cm at Stations 26 and 38 (western Lomonosov Ridge, 87–89 °N) and at Stations 48, 50, and 53 (Morris Jesup Rise, 84–85 °N) (Fig. 1).

One box core was collected at each of the five sampling stations and immediately subsampled after lifting the box corer onto the deck. To obtain stratified sediment samples for otolith analyses, 11–12 Plexiglas^®^ core liners (“push cores”, diameter 8.0 cm) were inserted into the box cores from the sediment surface to the underlying layers. Combined stratified samples were created by slicing the upper 0-15 cm of each push core at 1 cm intervals and pooling depth-equivalent strata from all otolith push cores within one box core into a single combined sample per 1 cm interval. Two additional push cores from each box core were stored at 4 °C for later stratigraphic and sedimentary analyses in the laboratory.

The combined samples were sieved directly on deck through a 300 µm mesh sieve with a 60 cm diameter. Otoliths visible to the naked eye were carefully collected from the sieve, and the remaining material was later sorted under a microscope in the onboard laboratory. Otoliths were scarce in the sediment samples and absent from 19 strata collected across the five sampling stations (Supplementary Data 2), despite sieving substantially larger volumes of stratified samples (9 dm^3^ per station) than are typically used in studies of microfossils such as foraminifers^62^ and ostracods^40^. We therefore maximized the otolith yield per sampling station by collecting as many additional otoliths as possible from the 0–15 cm of sediment remaining between the push cores (including those collected for other research projects) using the same sieving procedure. We refer to these non-stratified, non-quantitative sediment samples as “bulk” samples.

Altogether, one set of 131 otoliths was collected from the stratified samples within the push cores, and an additional set of 125 otoliths was taken from the top 0–15 cm of sediment between the push cores in the box cores (non-stratified bulk samples) (Fig. 1, Supplementary Data 2). Otoliths from bulk samples were unsuitable for stratigraphic analysis but could be used for species identification, morphometric measurements, and radiocarbon dating. Each of the 256 otoliths was assigned a unique ID number and photographed using a Leica M205C stereo microscope equipped with a digital camera (MC170 HD) connected to a computer running Leica Application Suite version 12.0.

### Identification of gadoid fish species

The past and present fish community composition and abundances in the CAO are virtually unknown. To constrain the likely species contributing to the sediment record, an inventory of fish species occurring in and around the CAO was compiled from existing literature^10,12,63^, followed by an examination of otolith morphology from these species using published identification guides^64–68^. This pre-assessment indicated that the sedimentary otoliths were dominated by two closely related Arctic gadoids, *Boreogadus saida* and *Arctogadus glacialis*. Species identification was based on a visual assessment of the three-dimensional otolith morphology, including the shape of the collicula, crenulations, lobing of the lateral surface, and length-width ratio (Supplementary Fig. 1). Extensive reference collections of *B. saida* otoliths at the Alfred Wegener Institute (Bremerhaven, Germany) allowed small interspecific variations in three-dimensional morphology to be recognized. In contrast, accessible collections of *A. glacialis* otoliths are lacking, and the species is rarely encountered in fisheries surveys. Identification of *A. glacialis* was therefore based on published three-dimensional descriptions and illustrations^66,67,69^. Many of the collected otoliths exhibited signs of prolonged burial, including boreholes, fragmentation, partial dissolution, and coatings of minerals or organic matter. Due to the varying levels of erosion and/or damages, each sediment otolith was given an identification confidence score from 1 to 5 (1 = very low confidence, 2 = low confidence, 3 = medium confidence, 4 = high confidence, 5 = very high confidence, Supplementary Fig. 3). Using this approach, 202 otoliths were identified as either *A. glacialis* or *B. saida* with an identification confidence score ranging from 1 to 5. Otoliths that could not be identified to the species level, but resembled both gadoid species, were classified as “unidentified gadoid”. Four non-gadoid otoliths were also identified, belonging to *Paraliparis* sp. (Supplementary Fig. 2).

### Two-dimensional feature analysis

We compared our species identifications with two-dimensional features extracted from photographic images using Fiji software^70^. Nine features were initially considered; area, perimeter, major axis, minor axis, elongation, solidity, roundness, ellipse, and aspect ratio (Supplementary Fig. 4). With non-normally distributed data, Spearman’s Rank Correlation Coefficients (r_S_) were calculated to evaluate the relationships among these nine morphometric features. Five morphometric features were excluded from further statistical analysis due to high collinearity (rS > 0.90) (Supplementary Fig. 4). A principal component analysis (PCA)^71^ was performed on the remaining two-dimensional features (area, solidity, ellipse, and aspect ratio) and principal components were plotted with the a priori determined species identifications, sizes, and confidence scores (Supplementary Fig. 5).

### Electron microscopy and elemental analysis

Scanning electron microscopy (SEM) images and energy dispersive X-ray spectroscopy (EDS) measurements were performed using an Axia ChemiSEM from ThermoFisher™. Otoliths were mounted on aluminium stubs using conductive double-sided carbon tape and analyzed without a conductive coating in low-vacuum mode. Elemental concentrations in different regions of an otolith represent the average value over 180 seconds per measurement (Supplementary Fig. 7). The regions for measuring the elemental concentrations were aligned roughly perpendicular to the sensor.

### Fish size reconstruction

Fish fork length (FL), defined as the length of a fish measured from the tip of its snout to the fork of the tail, was estimated for 186 identified, morphologically intact *A. glacialis* (49) and *B. saida* (129) using otolith length (OL) and previously published allometric relationships for *A. glacialis* (FL = 23.13*OL + 35.17) and *B. saida* (FL = 20.86*OL + 16.85)^72^. For otolith length, we used the major axis measurement as defined in the morphometric analysis.

### Radiocarbon dating

Accelerator mass spectrometry (AMS) ^14^C analysis was performed on 79 otoliths at the MICADAS - Mini Carbon Dating System laboratory at the Alfred Wegener Institute Helmholtz Center for Polar and Marine Research. The analysis used a carbonate handling system and a gas interface system connected to a Mini Carbon Dating System (CHS-GIS-MICADAS), following a standard operating procedure^73^. Forty-three otoliths came from stratified sediment samples, while 36 originated from bulk samples. Because the otoliths from stratified samples at Station 50 were poorly preserved, these samples were excluded from radiocarbon dating.

To ensure that radiocarbon dating of our otoliths was not influenced by autigenic calcite deposits on their outer surface, pore-water exchange on a diagenetically altered surface, or surface ^14^C contamination during sample processing, a leaching method was used to minimize potential errors in radiocarbon dating, as previously observed in foraminifers^31,74^. To meet the weight requirements for radiocarbon analysis, including leaching, small ( < 3 mm) otoliths were processed as whole, while only fragments were used from larger otoliths. Each whole otolith or fragment was carefully cleaned by manually scraping off metal deposits, and sediment remains from boreholes made by invertebrates using a metal scalpel. Radiocarbon dating was performed twice for each otolith sample. The first measurement was taken from the leachate after about 36 ± 2% (mean ± % standard deviation, n = 79 otoliths) of the outer part had been leached with 0.05 M HCl. The second measurement was taken from the otolith residue after leaching, following further hydrolysis of the inner part with ≥ 85% H_3_PO_4_, until complete dissolution^74^.

The results of the AMS measurements were normalized against the Oxalic Acid II reference gas (CO_2_ produced from Oxalic Acid II, NIST SRM4990C) and blank-corrected against ^14^C-free CO_2_ reference gas (leachate) or against sample size-matched and leached ^14^C-free IAEA-C1 (remains) processed and analyzed in the same sequence. Normalization and blank correction were performed using BATS software^75^. The leaching treatment was effective, as the inner otolith parts were on average 8 ± 10% (mean ± % standard deviation) older than the outer parts, and this difference was even more pronounced for inner parts of otoliths older than 20 ka, which were on average 20 ± 8% (up to 37%) older than the outer parts (Supplementary Data 4). SEM analyses of otolith fragments showed no authigenic overgrowth (Supplementary Fig. 7), so overall well-preserved otoliths were measured. However, the otoliths exhibited a rough surface texture (even in modern specimens), which increases the likelihood of porewater-induced or sample-treatment-induced ^14^C-alterations. As only the otolith surface was affected, potential ^14^C-alterations were effectively eliminated by the leaching method.

The software OxCal (version 4.4) was used to calibrate radiocarbon ages to calendar ages^76^ using the Marine20 calibration curve^77^. The regional reservoir correction (ΔR) is notoriously difficult to determine for the Arctic Ocean because of the lack of suitable reference material, i.e., historic samples of known age predating 1950. We used ΔR_marine20_ = 300 ± 150 years based on recently updated values of 300 ± 300 years^78^ and 330 ± 41 years^79^. However, while these values might be realistic for the Holocene, ΔR could be larger during glacial periods due to limited mixing of carbon between the atmosphere and the ice-covered Arctic Ocean^80^. Therefore, the glacial ^14^C ages reported in this paper should be considered minimum radiocarbon ages.

### Stratigraphy and sedimentology

Two push cores from each sampling station were used for stratigraphic and sedimentary analysis at the Department of Geological Sciences, Stockholm University, to document depositional conditions along the cores. Core photographs were taken using the Linescan function of a Geotek multi-sensor core logger. Contrast and brightness were slightly adjusted to enhance the visualization of sedimentary structures. X-ray fluorescence (XRF) measurements were performed on the archive half of the split cores using an ITRAX XRF core scanner. The molybdenum tube was set at 55 kV and 50 mA, with a step size of 2 mm and an exposure time of 25 seconds. Grain-size measurements were conducted with a Malvern Mastersizer 3000 laser diffraction particle size analyzer. Wet samples were immersed in a dispersing agent ( < 10% sodium hexametaphosphate solution) and placed in an ultrasonic bath to disaggregate particles, then poured into the Malvern measurement chamber.

### Reporting summary

Further information on research design is available in the Nature Portfolio Reporting Summary linked to this article.

## Supplementary information

Supplementary Information

Description of Additional Supplementary Files

Supplementary Data 1

Supplementary Data 2

Supplementary Data 3

Supplementary Data 4

Reporting Summary

Transparent Peer Review file

## Source data

Source Data

## Data Availability

Data collected during the Swedish Synoptic Arctic Survey (SAS) expedition with the icebreaker *Oden* in 2021, including photographs of all 256 otoliths analyzed in this study, are available at the Bolin Centre Database (https://bolin.su.se/data/oden-sas-2021-expedition). The specimen identifiers used in this database are provided in Supplementary Data 3, along with the results of all measurements performed on each otolith. The radiocarbon-dated otoliths were destroyed during laboratory processing; the remaining (parts of) the otoliths are stored at the Alfred Wegener Institute in Bremerhaven, Germany (Courtesy Hauke Flores). All numerical data on which this study is based are included in Supplementary Data 1-4. Source data are provided with this paper.
