# Supplementary material for: Survival and divergence of Arctic endemic cod species during 50,000 years of climate change
Source: Nat Commun. 2026 Oct 16;17:10876. doi: 10.1038/s41467-026-78810-4 (PMC13675226; doi:10.1038/s41467-026-78810-4)
Supplement: Supplementary file 1 [file 41467_2026_78810_MOESM1_ESM.pdf]

Supplementary Figure 1

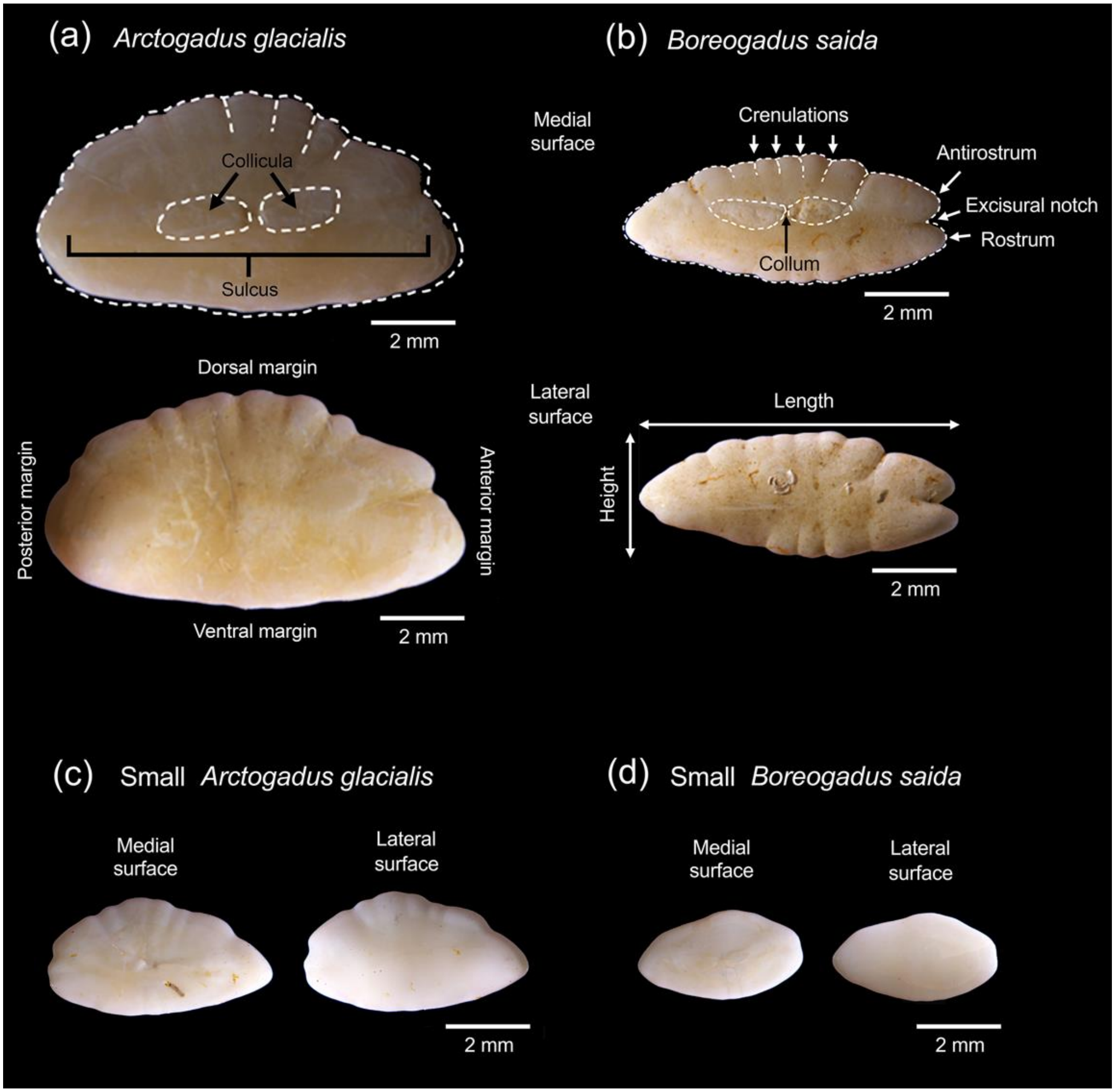

Supplementary Fig. 1. Comparative morphological features of the otoliths of the two Arctic endemic gadoids *Arctogadus glacialis* and *Boreogadus saida*. a *A. glacialis*, otolith SO21_BC5_BULK_OTObc394. **b** *B. saida*, otolith SO21_BC5_BULK_OTObc345. **c** Small *A. glacialis* otolith SO21_BC5_BULK_OTObc325. **d** Small *B. saida* otolith SO21_BC5_BULK_OTObc324. Gadoid otoliths have a straight sulcus with a pseudobiostial opening with clearly divided collicula (also referred to as advanced homosulcoid), with the collicula closed far from the otolith edge[1]. The adult otolith morphology of *A. glacialis* and *B. saida* is characterized by relatively flat medial and lateral surfaces, in contrast to the more concave surfaces of subarctic Atlantic and Pacific gadoids, including Atlantic cod (*Gadus morhua*), haddock (*Melanogrammus aeglefinus*), walleye pollock (*Gadus chalcogrammus*),

Pacific cod (*Gadus macrocephalus*), and Greenland cod (*Gadus ogac*)[1,2,3,4,5,6]. The otolith sulci of *A. glacialis* and *B. saida* display collicula or raised portions of the sulcus floor, and a constriction (collum), usually located near the middle of the sulcus. In subarctic gadoids, the collicula are more pronounced and extend over the entire length of the otolith[4]. Adult *B. saida* otoliths have more crenulations (notches) around the entire margin than *A. glacialis* and are more elongated than the more elliptical otoliths of *A. glacialis*. The anterior margin has an excisural notch (excisura major), an opening of the sulcus near the otolith margin that separates the extension of the rostrum from the antirostrum of the otolith. The posterior end of the otolith is thickened, and the sulcus opening is minor (excisura minor) or absent in both *A. glacialis* and *B. saida*. Reconstructed body length, based on otolith length-to-body length equations[7], suggests that all our *B. saida* otoliths belong to stage 1+ individuals with standard length >5.5 cm[8] (Supplementary Data 3). However, both *A. glacialis* and *B. saida* typically reach sexual maturity at age 2+. The juvenile forms of the Arctic gadoids' otoliths are poorly described in the available literature, with only one example of a "juvenile" *B. saida* individual measuring 7.4 cm in body length[4]. Although small otoliths are difficult to identify to the species level, they do display the early features of the adult otoliths. The collicula of *A. glacialis* and *B. saida* are already visible in small otoliths. The margins of juvenile otoliths have relatively indistinct crenulations and develop only into moderately pronounced crenulations in their adult forms. In the otoliths of juvenile subarctic gadoids, crenulations are much more pronounced, consistent with the highly pronounced crenulations of their adult forms. The medial surface of the Arctic gadoid otoliths is relatively smooth, whereas in subarctic gadoids this surface is strongly lobed.

Supplementary Figure 2

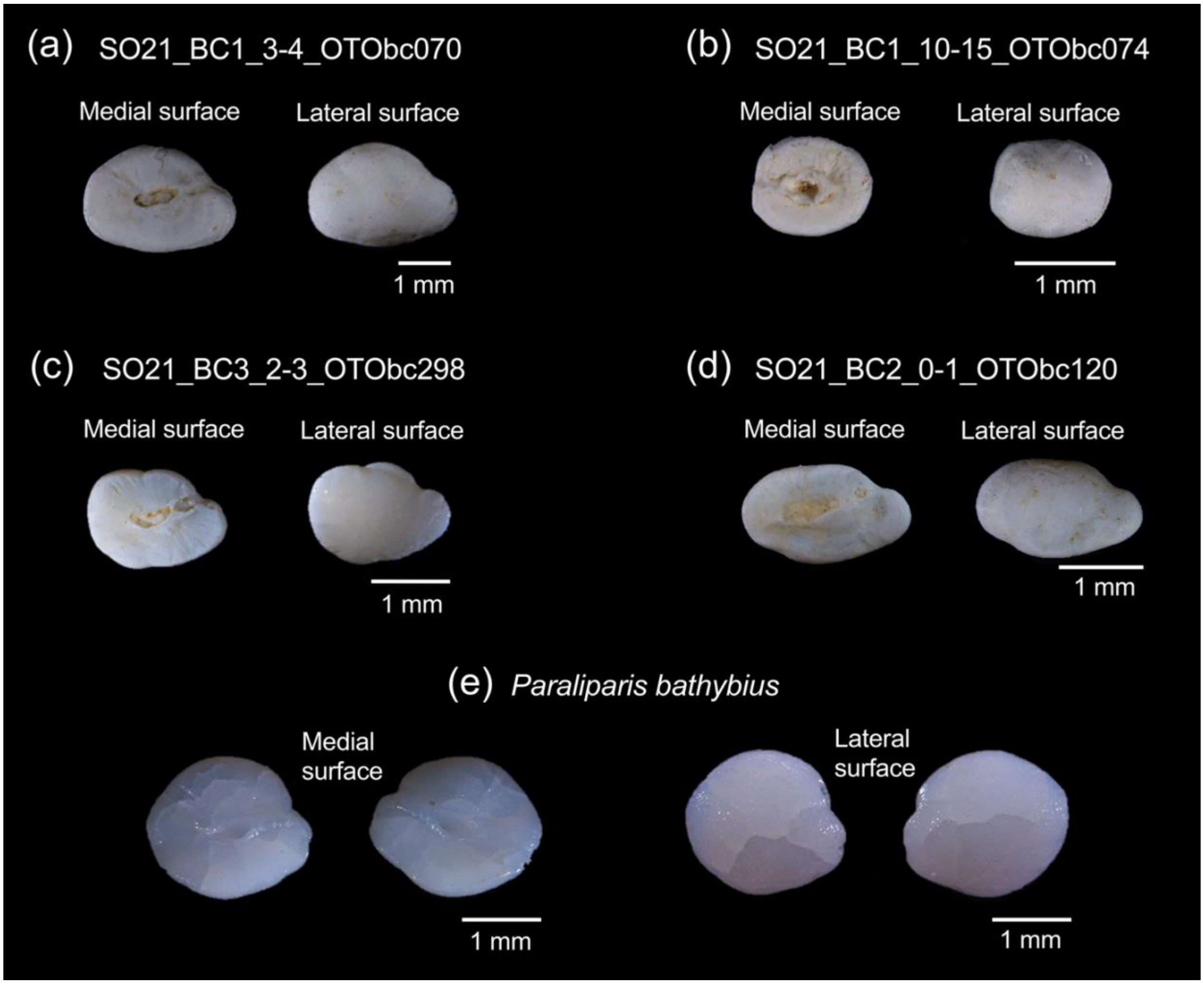

Supplementary Fig. 2. Comparison of the four *Paraliparis* sp. otoliths found in sediment samples with otoliths from a *Paraliparis bathybius* individual caught in the Central Arctic Ocean in 2020. **a** Station 26, stratified push-core sample, stratum 3–4 cm. **b** Station 26, stratified push-core sample, stratum 10–15 cm. **c** Station 48, stratified push-core sample, stratum 2–3 cm. **d** Station 38, stratified push-core sample, stratum 0–1 cm. **e** For comparison: the two otoliths from a *Paraliparis bathybius* individual caught in the Central Arctic Ocean in February 2020 during the MOSAiC expedition[9]. *P. bathybius* is a common deep-water demersal snailfish in the Arctic and northwest Atlantic Ocean, and its otoliths are well-described[10].

Supplementary Figure 3

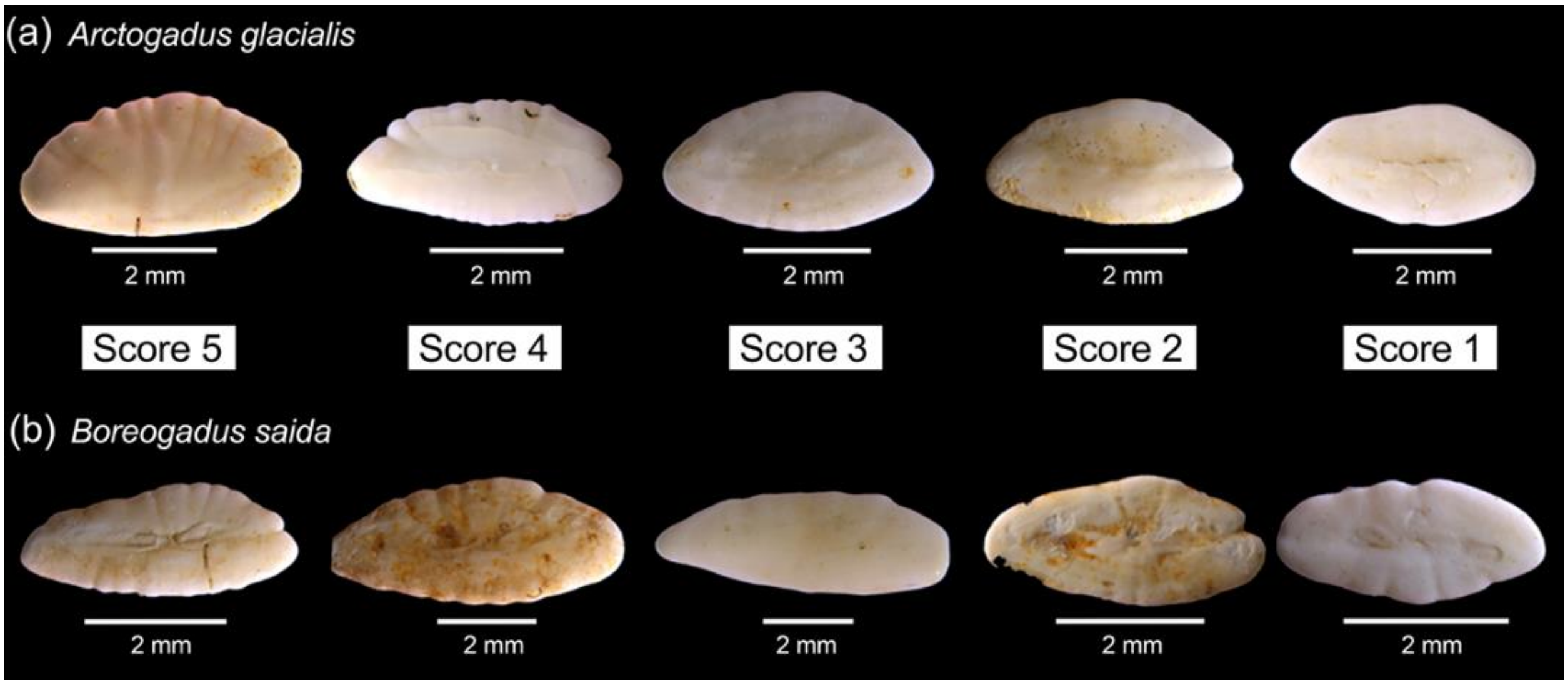

Supplementary Fig. 3. Examples of otoliths with different identification confidence scores. **a** *Arctogadus glacialis*. **b** *Boreogadus saida*. These examples show otoliths that received different confidence scores during visual identification of the n=202 gadoid otoliths that could be identified to the species level: 1 = very low confidence, 2 = low confidence, 3 = medium confidence, 4 = high confidence, 5 = very high confidence. The identification confidence scores were affected by erosion and/or damage that obscured three-dimensional otolith features: 22 were chipped, 75 showed signs of dissolution/diagenesis, 45 had boreholes made by invertebrates, and 30 were coated with a thin dark diagenetic layer (Supplementary Data 3). In the top cm of the sediment, otoliths were more frequently chipped (10%) and/or had boreholes (29%) than those in the 14 cm thick layer below (3 and/or 5%, respectively), but fewer otoliths showed signs of partial dissolution/diagenesis (14%) and/or were coated (10%) compared to those in the 14 cm below (28 and/or 19%, respectively). Additionally, smaller otoliths generally received lower identification confidence scores than larger ones, consistent with the two-dimensional morphometric analysis (Supplementary Fig. 5). Undamaged smaller otoliths displayed typical features of Arctic gadoids, as described and illustrated in Supplementary Fig. 1, but these features were less distinct than in larger otoliths.

Supplementary Figure 4

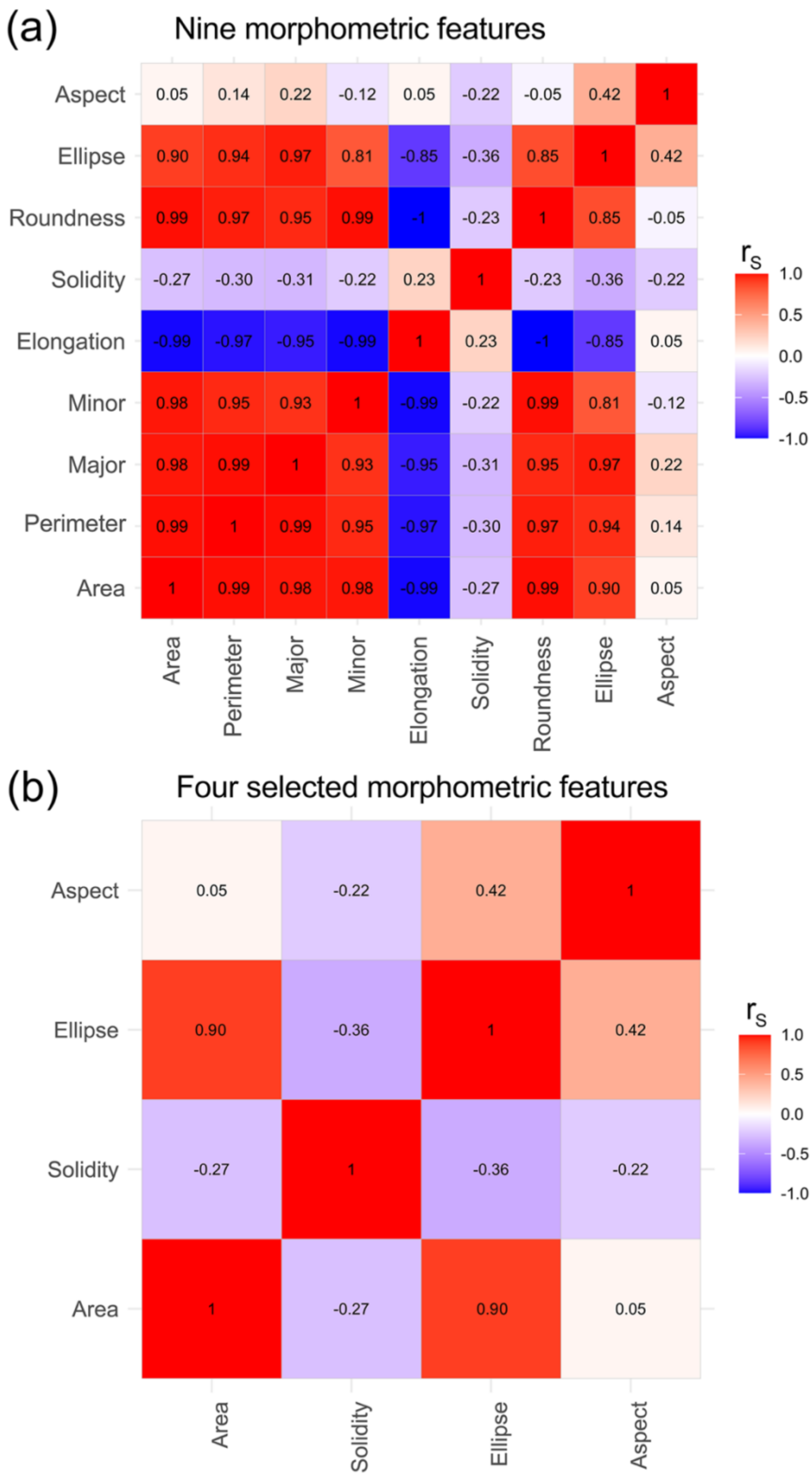

Supplementary Fig. 4. Spearman's Rank Correlation Coefficients (r_S) between two-dimensional morphometric features of the otoliths. The correlation analyses were based on two-dimensional morphometric features from n=206 gadoid otoliths (49 *Arctogadus glacialis*, 129 *Boreogadus saida*, 28 unidentified gadoids) for which measurements could be obtained, i.e., morphologically intact otoliths. Since two species were involved, the data were not normally distributed. r_S values above 0.14 (positive correlation) and below -0.14 (negative correlation) were significant ($p>0.05$). **a** All nine tested two-dimensional morphometric features. **b** The four two-dimensional morphometric features selected for principal component analysis (PCA, Supplementary Fig. 5) after discarding the five features with $r_S>0.90$. Aspect = the ratio between the major and the minor axes, Ellipse = the difference between the major and minor axes in mm, Roundness = $(4*area)/(\pi*major\_axis^2)$, where a value of 1 indicates a perfect circle and a values greater than 1 indicate an increasingly non-circular shape, Solidity = the otolith area divided by its convex area (also known as "convexity"), Elongation = 4*perimeter /area, Minor axis = the secondary axis of the best-fitting ellipse in mm, Major axis = the primary axis of the best-fitting ellipse in mm, Perimeter = the length of the outer boundary of the otolith in mm, Area = the surface area of the otolith in mm^2. The correlation matrix was calculated using the R "factoextra" package[11]. Source data are provided as a Source Data file.

Supplementary Figure 5

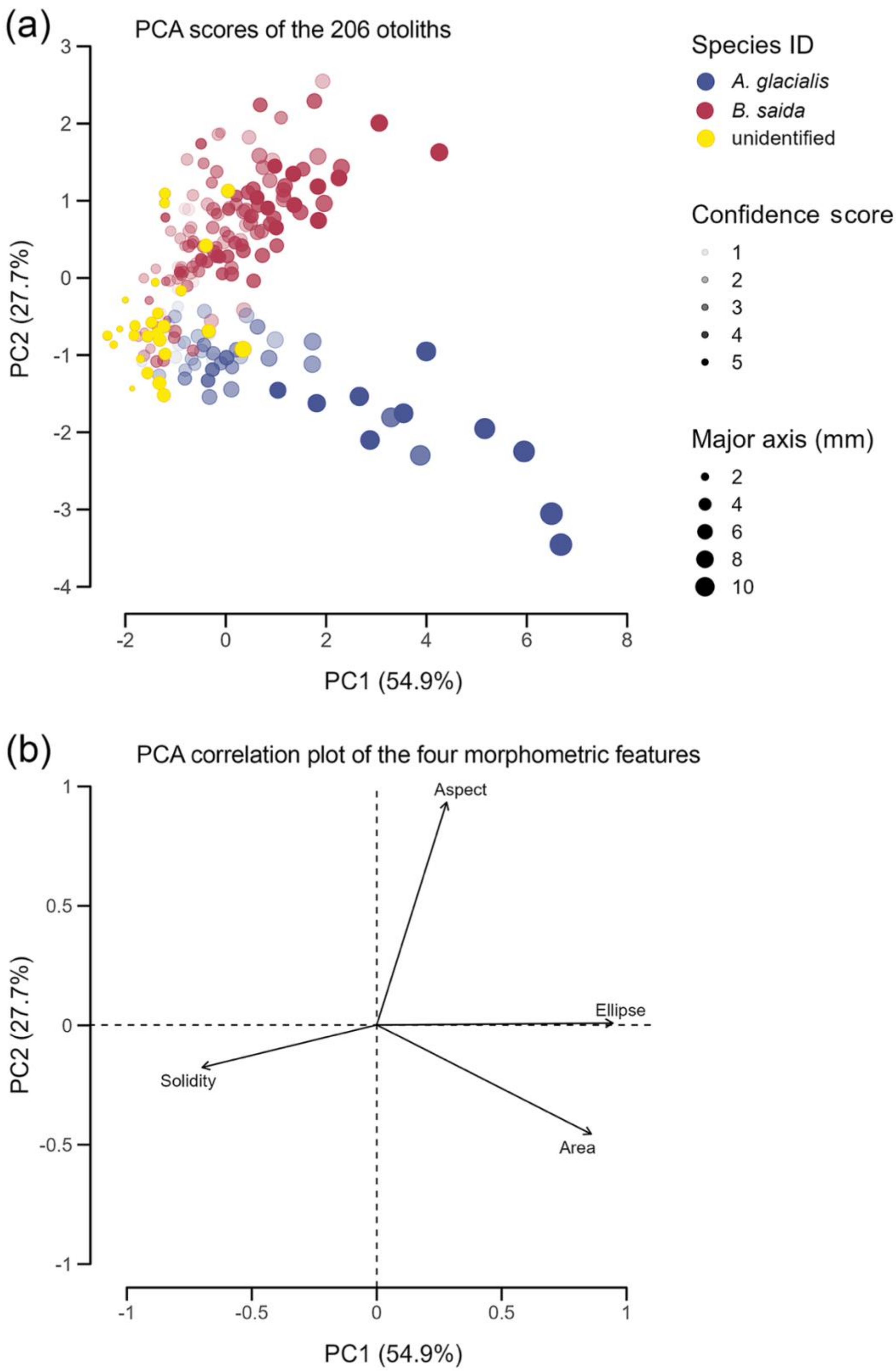

Supplementary Fig. 5. Results of principal component analysis (PCA) based on two-dimensional morphometric features. a PCA scores for each otolith in relation to species identification (dot color), identification confidence score (dot color intensity), and otolith length (dot size). **b** PCA correlation plot displaying the contribution of each of the four tested morphometric features along the first two PCA axes. The PCA was performed using four two-dimensional morphometric features from n=206 gadoid otoliths (49 *Arctogadus glacialis*, 129 *Boreogadus saida*, 28 unidentified gadoids) for which measurements could be obtained, i.e., morphologically intact otoliths. The input data for the PCA included: Area = the surface area of the otolith in mm^2, Solidity = the otolith area divided by its convex area (also known as "convexity"), Ellipse = the difference between the major and minor axes in mm, and Aspect = the ratio between the major and minor axes. Five morphometric features were excluded from the PCA analyses due to high collinearity (Supplementary Fig. 4), which could bias PCA results by overemphasizing certain eigenvectors. Our species identifications, based on three-dimensional microstructures of the otoliths, along with their identification confidence scores and otolith length, were passively overlaid (not included in the statistical analysis) on the ordination plot for comparison with the PCA results, which relied solely on the four two-dimensional morphometric features. Ellipse contributed most to the PC1 axis, and the aspect ratio contributed most to the PC2 axis. The PCA was performed using the R "stats" package[11]. Source data are provided as a Source Data file.

Supplementary Figure 6

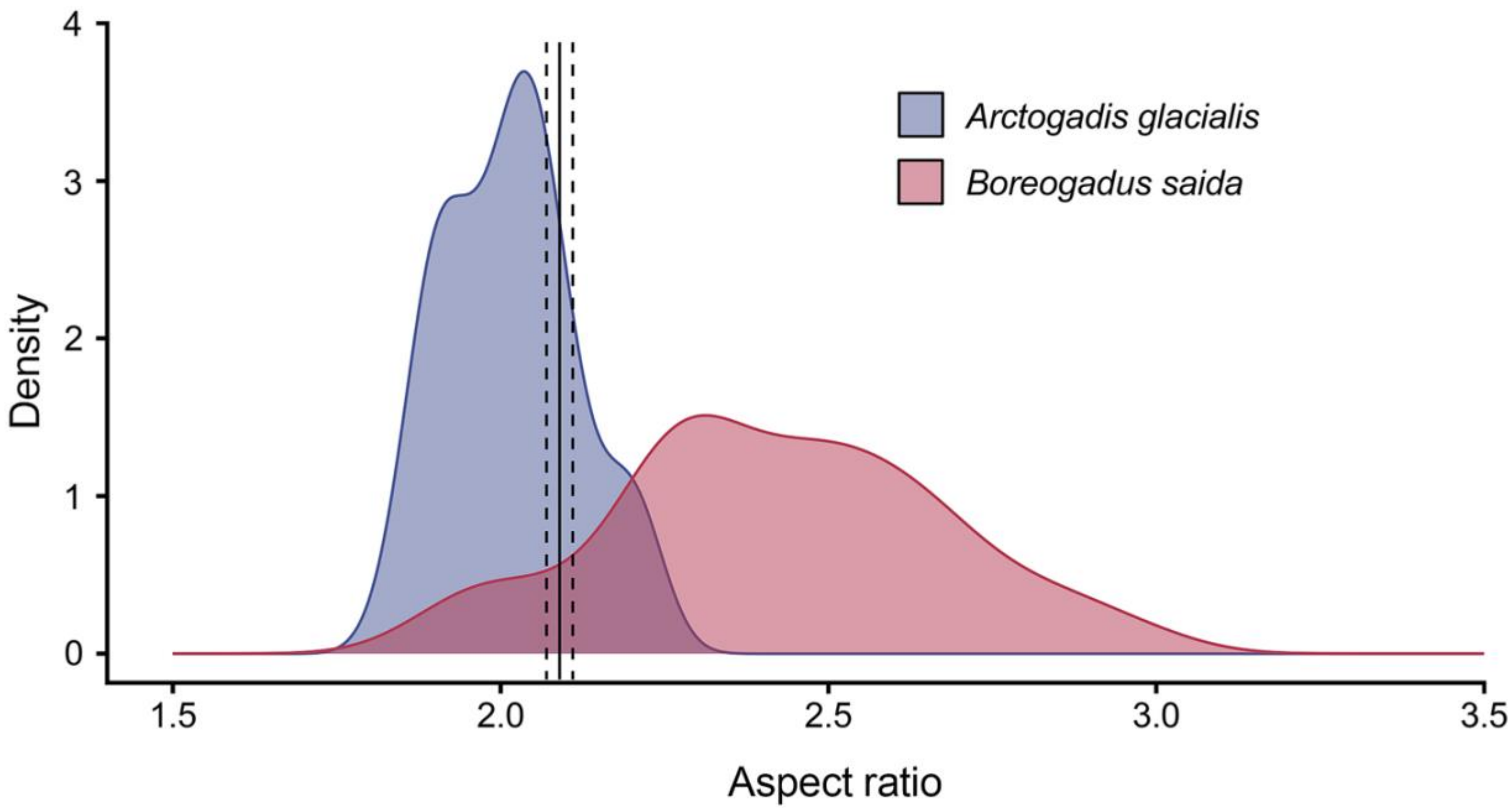

Supplementary Fig. 6. Results of a binomial logistic regression model predicting the species identity of *Arctogadus glacialis* and *Boreogadus saida* based solely on the aspect ratio of their otoliths. The model uses the n=178 identified otoliths (49 *A. glacialis*, 129 *B. saida*) from which two-dimensional morphometric measurements could be obtained, i.e., morphologically intact otoliths. The aspect ratios of the two species overlapped: for *A. glacialis*, it was 2.02 ± 0.10 (mean ± standard deviation), range 1.84–2.23, n=49 otoliths, and for *B. saida*, 2.43 ± 0.25, range 1.88–3.00, n=129 (Supplementary Data 3). The binomial logistic regression model predicts that an aspect ratio of 2.09 ± 0.02 (standard error) corresponds to a 0.5 probability of correctly distinguishing between the two species. However, overlap in aspect-ratio ranges reduces the reliability of using aspect ratio alone for species identification. The analysis was performed using the R "stats" package[11]. Source data are provided as a Source Data file.

Supplementary Figure 7

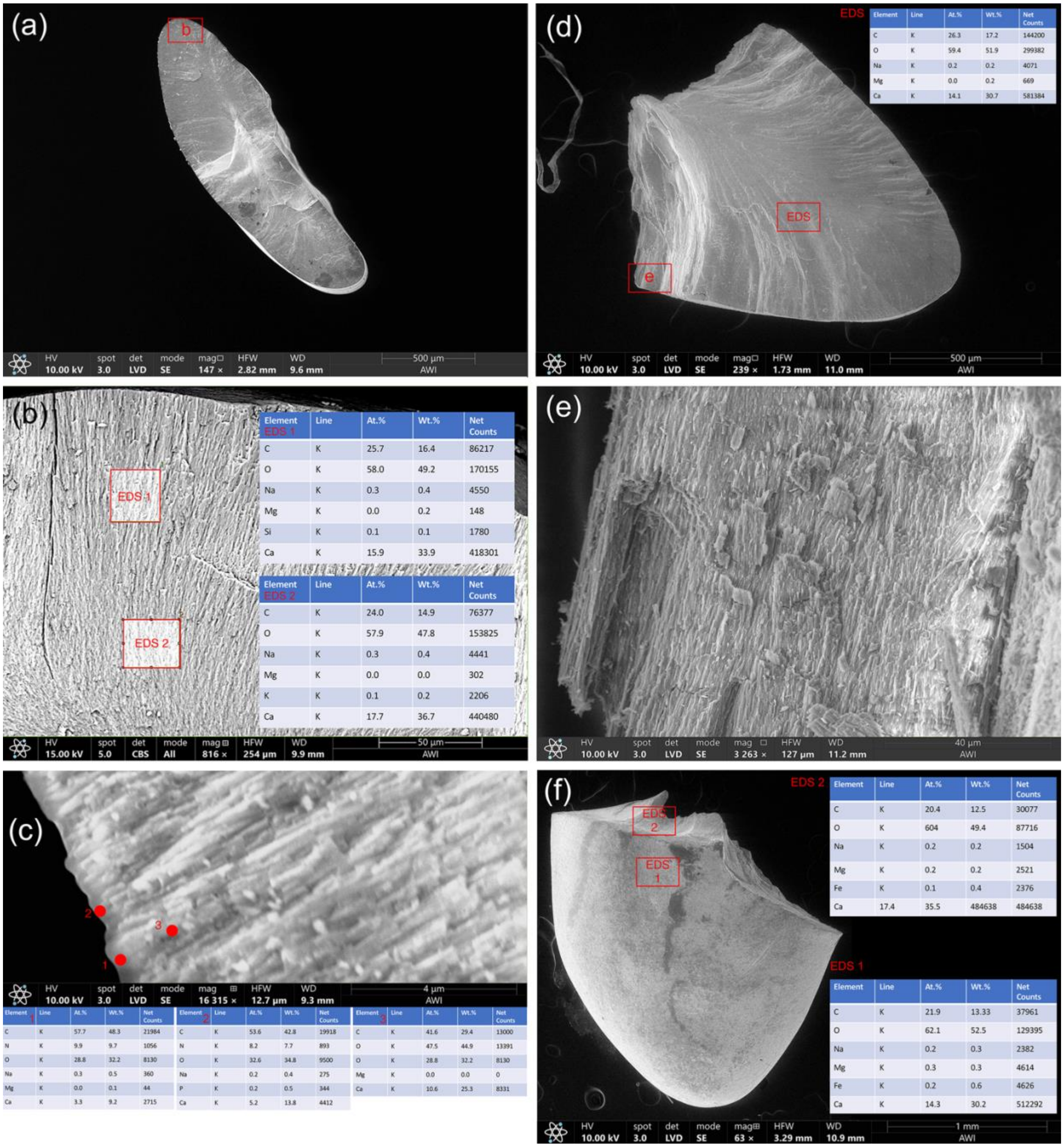

Supplementary Fig. 7. Comparison of the structure and elemental composition of recent and sediment otoliths using Scanning Electron Microscopy (SEM) / Energy Dispersive Spectroscopy (EDS). Elemental concentrations in different parts of an otolith (shown as squares in the images) represent the average over 180 seconds for each measurement. The regions used to measure the elemental concentrations were aligned roughly perpendicular to the sensor. **a** Recent *Boreogadus saida* otolith (PS106.2 BSA46_1, AWI Collection). **b** Fine structure of the

same otolith with EDS results from the area indicated in a. **c** Close-up of the same otolith, showing EDS results of the organic coating at the otolith surface (1,2) and further away from the surface. **d** Fragment of a sediment otolith from Station 50 (*Arctogadus glacialis*, SO21_BC5_BULK_OTObc394) with EDS results. **e** Fine structure of the same otolith from the area indicated in d. **f** Fragment of a sediment otolith from Station 50 (*B. saida*, SO21_BC5_BULK_OTObc371) with EDS results, showing that an organic surface coating is absent. These SEM/EDS analyses show no authigenic overgrowth and no differences in structure or chemical composition between recent (*B. saida*) and sediment otoliths (*A. glacialis* and *B. saida*), except that recent otoliths have an organic surface coating (rich in C and N) that is missing in the sediment otoliths.

Supplementary references

1. Svetocheva, O., Stasenkova, N. & Fooks, G. Guide to the otoliths of the bony fishes of the White Sea. IMR/PINRO Joint Report Series **3**, 1–46 (2007).
2. Boulva, J. Morphometries of three sympatric Arctic codfishes of the genera *Arctogadus* and *Gadus*. *Journal of the Fisheries Research Board of Canada* **29**, 243–249 (1972).
3. Frost, K. J. Descriptive key to the otoliths of gadid fishes of the Bering, Chukchi, and Beaufort Seas. *Arctic* **34**, 55–59 (1981).
4. Härkönen T. *Guide to the Otoliths of the Bony Fishes of the Northeast Atlantic*. Danbiu ApS., Hellerup, Denmark. 256 pp. (1986).
5. Lidster, W. W., Lilly, G. R. & Dawe, E. G. Otoliths of Arctic cod (*Boreogadus saida*), small Atlantic Cod (*Gadus morhua*), and three other fish species from Newfoundland waters: Description and relationship of body length to otolith lengths. *Journal of Northwest Atlantic Fishery Science* **16**, 33–40 (1994).
6. Nolf, D. & Steurbaut, E. Evidence from otoliths for establishing relationships within gadiforms. *Science Series, Natural History Museum of Los Angeles County* **32**, 89–111 (1989).
7. Finley, K. J., Bradstreet, M. S. W. & Miller, G. W. Summer feeding ecology of harp seals (*Phoca groenlandica*) in relation to Arctic cod (*Boreogadus saida*) in the Canadian High Arctic. *Polar Biology* **10**, 609–618 (1990).
8. Geoffroy, M. et al. The circumpolar impacts of climate change and anthropogenic stressors on Arctic cod (*Boreogadus saida*) and its ecosystem. *Elementa: Science of the Anthropocene* **11**, 00097 (2023).
9. Snoeijs-Leijonmalm, P. et al. Unexpected fish and squid in the central Arctic deep scattering layer. *Science Advances* **8**, eabj7536 (2022).
10. Campana, S.E. *Photographic Atlas of Fish Otoliths of the Northwest Atlantic Ocean*. NRC Research Press, Ottawa, Canada. 284 pp. (2004).
11. R Core Team, A Language and Environment for Statistical Computing. R Foundation for Statistical Computing, Vienna (2025). https://www.R-project.org
