# Supplementary material for: Survival and divergence of Arctic endemic cod species during 50,000 years of climate change
Source: Nat Commun. 2026 Oct 16;17:10876. doi: 10.1038/s41467-026-78810-4 (PMC13675226; doi:10.1038/s41467-026-78810-4)
Supplement: Supplementary file 2 [file 41467_2026_78810_MOESM2_ESM.pdf]

Description of Additional Supplementary Files

Supplementary Data 1- Properties of the five sampling stations where the box cores used in this study were collected during the Synoptic Arctic Survey (SAS) expedition with the Swedish icebreaker Oden (SO21). Device_operation = an expedition code that includes the expedition name (SO21), the station number, and the cast number on the sampling date. Box_core_ID = the box core identifier according to the Expedition Logbook (BC1, BC2, BC3, BC5, BC6). Time_bottom_UTC = the Coordinated Universal Time (UTC) when the boxcore sampler reached the seafloor. Depth_bottom_m = the water depth in m at the sampling station measured by EK80 acoustics when the box-core sampler hit the seafloor. Longitude_bottom_DM = the longitude when the box-core sampler hit the seafloor in degrees and minutes (DM). Latitude_bottom_DM = the latitude when the box-core sampler hit the seafloor in degrees and minutes (DM).

Supplementary Data 2- Otolith counts and density in the sediment samples. Sampling_station = the number of the sampling station. Box_core_ID = the box core identifier according to the Expedition Logbook (BC1, BC2, BC3, BC5, BC6). Sample_contents = a description of the sediment sub-sample from which the otoliths were collected. This includes two types of samples: (1) Stratified samples with the top 0–10 cm analyzed per 1 cm sediment stratum and the combined 10–15 cm stratum. (2) Non-stratified bulk samples from the top 0–15 cm of sediment collected between push cores. Number_of_otoliths = the number of otoliths collected. Number_of_pushcores = the number of push cores inserted into the box core for collecting otoliths. Pushcore_diameter_cm = the diameter of the push cores in cm. Stratum_thickness_cm = the thickness of the sampled stratum in cm. Sediment_volume_dm3 = calculated sediment volume in dm3. Otoliths_per_dm3 = calculated number of otoliths per dm3. N/A = not applicable.

Supplementary Data 3- Properties of n=256 otoliths sampled from the five box-core sediment samples. Otolith_ID = a unique identifier assigned to each individual otolith. File_name_photograph = the name of the photographic file for each individual otolith in the open-access Bolin Centre Database at Stockholm University (https://bolin.su.se/data/oden-sas-2021- expedition). Sampling_station = the number of the sampling station. Box_core_ID = the box core identifier according to the Expedition Logbook (BC1, BC2, BC3, BC5, BC6). Stratum_interval_cm = the sediment depth interval in cm for stratified samples or “bulk” for combined samples from the upper 15 cm of the sediment collected between the push cores. Stratum_nr = a sequential number for each stratum per station. Completeness = whether the otolith is intact or partly missing (“chipped”). Worn = whether the otolith is worn by dissolution/diagenesis. Boreholes = whether the otolith has boreholes made by invertebrates. Coated = whether the otolith is coated with a thin dark diagenetic layer. Identified = whether it was possible to identify the fish species from the otolith (yes/no). ID_conf_score = the identification confidence score: 1 = very low confidence, 2 = low confidence, 3 = medium confidence, 4 = high confidence, 5 = very high confidence. Species_name = the species name or “unidentified gadoid”. AMS_14C_dated =

whether the otolith was radiocarbon dated by accelerator mass spectrometry (AMS) (yes/no). 14C_CALIB_mean = calibrated radiocarbon age in thousand years before present (ka BP). 14C_CALIB_SD = standard deviation for the calibrated radiocarbon age in ka BP. Morphometrics = whether it was possible to collect morphometric data from the otolith (yes/no). Area_mm2 = surface area of the otolith in mm2. Perimeter_mm = length of the outer boundary of the otolith in mm. Major_axis_mm = primary axis of the best-fitting ellipse in mm. Minor_axis_mm = secondary axis of the best-fitting ellipse in mm. Elongation = 4*perimeter /area. Solidity = the otolith area divided by its convex area (also known as "convexity"). Roundness = (4*area)/(π*major_axis2), where a value of 1 indicates a perfect circle and a value greater than 1 indicates an increasingly non-circular shape with increasing difference from 1. Ellipse = the difference between the major and minor axes in mm. Aspect ratio = the ratio between the major and minor axes. Weighed = whether it was possible to weigh the otolith (yes/no). Mass_mg = weight of the otolith in milligrams. Aged = whether it was possible to estimate the fish age at death from growth increments (yes/no). Age_at_death_y = fish age at death in years. Fish size = whether it was possible to reconstruct fish fork length (FL) data from the otolith (yes/no). Reconstructed_FL_cm = reconstructed fork length (length of a fish measured from the tip of its snout to the fork of the tail) in cm. N/A = not applicable.

Supplementary Data 4- Results of radiocarbon dating of n=79 otoliths. Otolith_ID = a unique identifier assigned to each individual otolith. Sampling_station = the number of the sampling station. Box_core_ID = the box core identifier according to the Expedition Logbook (BC1, BC2, BC3, BC5, BC6). Stratum_interval_cm = the sediment depth interval in cm for stratified samples or "bulk" for combined samples from the upper 15 cm of the sediment. Stratum_nr = a sequential number for each stratum per station. Species_name = the species name or "unidentified gadoid". AWI_ID = the analysis number assigned by the 14C laboratory at the Alfred Wegener Institute, Bremerhaven. L refers to the CHS-GIS leachate (200 µL 0.05 M HCl), R refers to the CHSGIS remains of the otolith after leaching (200 µL ≥ 85% H3PO4). F14C = fraction modern carbon. 1sigma = one standard deviation. 14C_age_yBP = radiocarbon age in years before present. Cweight_µgC = carbon weight in micrograms C_leachate = percentage of carbon weight in the leachate. R_age-L_age_y = difference between the uncalibrated radiocarbon age of the otolith after leaching and that of the leachate in years. Age_difference_% = percentage difference between the uncalibrated radiocarbon age of the otolith after leaching and that of the leachate. 14C_CALIB_mean = mean calibrated radiocarbon age. 14C_CALIB_SD = standard deviation of the mean calibrated radiocarbon age. CALIB_median = median calibrated radiocarbon age. CALIB_95%_upper = upper 95% confidence of median calibrated radiocarbon age. CALIB_95%_lower = lower 95% confidence of median calibrated radiocarbon age. N/A = not applicable.
