# Supplementary material for: Survival and divergence of Arctic endemic cod species during 50,000 years of climate change
Source: Nat Commun. 2026 Oct 16;17:10876. doi: 10.1038/s41467-026-78810-4 (PMC13675226; doi:10.1038/s41467-026-78810-4)
Supplement: Supplementary file 3 [file 41467_2026_78810_MOESM7_ESM.pdf]

Corresponding author(s): Pauline Snoeijs-Leijonmalm

Last updated by author(s): Aug 17, 2026

Reporting Summary

Nature Research wishes to improve the reproducibility of the work that we publish. This form provides structure for consistency and transparency in reporting. For further information on Nature Research policies, see our Editorial Policies and the Editorial Policy Checklist.

Statistics

For all statistical analyses, confirm that the following items are present in the figure legend, table legend, main text, or Methods section.

n/a	Confirmed	
☐	☒	The exact sample size (n) for each experimental group/condition, given as a discrete number and unit of measurement
☐	☒	A statement on whether measurements were taken from distinct samples or whether the same sample was measured repeatedly
☐	☒	The statistical test(s) used AND whether they are one- or two-sided<br>*Only common tests should be described solely by name; describe more complex techniques in the Methods section.*
☒	☐	A description of all covariates tested
☐	☒	A description of any assumptions or corrections, such as tests of normality and adjustment for multiple comparisons
☐	☒	A full description of the statistical parameters including central tendency (e.g. means) or other basic estimates (e.g. regression coefficient) AND variation (e.g. standard deviation) or associated estimates of uncertainty (e.g. confidence intervals)
☒	☐	For null hypothesis testing, the test statistic (e.g. F, t, r) with confidence intervals, effect sizes, degrees of freedom and P value noted<br>*Give P values as exact values whenever suitable.*
☒	☐	For Bayesian analysis, information on the choice of priors and Markov chain Monte Carlo settings
☒	☐	For hierarchical and complex designs, identification of the appropriate level for tests and full reporting of outcomes
☐	☒	Estimates of effect sizes (e.g. Cohen's d, Pearson's r), indicating how they were calculated

Our web collection on statistics for biologists contains articles on many of the points above.

Software and code

Policy information about availability of computer code

Data collection	We did not construct new codes = we used the standard implementations in the R statistical packages.
Data analysis	As described in the manuscript, we used the following statistical analyses and software: Principal Components Analysis (PCA) implemented with the R 'stats' package (2025, reference provided). 'FactoMineR' (2008, reference provided) was used was used to visualize multivariate patterns in morphometric features. The software Oxcal (version 4.4) was used to calibrate radiocarbon age to calendar age (2009, reference provided).

For manuscripts utilizing custom algorithms or software that are central to the research but not yet described in published literature, software must be made available to editors and reviewers. We strongly encourage code deposition in a community repository (e.g. GitHub). See the Nature Research guidelines for submitting code & software for further information.

Data

Policy information about availability of data

All manuscripts must include a data availability statement. This statement should provide the following information, where applicable:

- Accession codes, unique identifiers, or web links for publicly available datasets
- A list of figures that have associated raw data
- A description of any restrictions on data availability

Data collected during the Swedish Synoptic Arctic Survey (SAS) expedition with the icebreaker Oden in 2021, including photographs of all 256 otoliths analyzed in this study, are available at the Bolin Centre Database (https://bolin.su.se/data/oden-sas-2021-expedition). The specimen identifiers used in this database are provided in the Supplementary Data file, along with the results of all measurements performed on each otolith. The radiocarbon-dated otoliths were destroyed during laboratory processing; the remaining (parts of) the otoliths are stored at the Alfred Wegener Institute in Bremerhaven, Germany (Courtesy Hauke Flores). All

numerical data on which this study is based are included in the Supplementary Data file. The source data for all figures in this study are included in the Source Data file.

Field-specific reporting

Please select the one below that is the best fit for your research. If you are not sure, read the appropriate sections before making your selection.

☐ Life sciences ☐ Behavioural & social sciences ☒ Ecological, evolutionary & environmental sciences

For a reference copy of the document with all sections, see nature.com/documents/nr-reporting-summary-flat.pdf

Ecological, evolutionary & environmental sciences study design

All studies must disclose on these points even when the disclosure is negative.

Study description	We analyzed fish ear stones (otoliths) from marine sediments and found that two Arctic endemic fishes, polar cod (Boreogadus saida) and ice cod (Arctogadus glacialis), lived at 84-89 °N between 48 and 35 ka BP, and again since the beginning of the Holocene about 12 ka BP. Around 20 ka BP, during the Last Glacial Maximum, ice cod were present north of Greenland, suggesting that refugia for its survival and divergence from polar cod existed during the harshest climatic conditions.
Research sample	Deep-sea sediment samples with otoliths were taken in the Central Arctic Ocean from ice-breaker Oden with a large box-corer.
Sampling strategy	Sample size depended on the number of otoliths recovered from box-core subsamples from 5 sampling stations.
Data collection	Julek Chawarski and Pauline Snoeijs-Leijonmalm collected the otoliths on board. Hauke Flores and Nicole Hildebrand photographed and catalogized the sampled otoliths on board.
Timing and spatial scale	August, 19, 2021 - 6 September 2021, Lomonosov Ridge and Morris Jersup Rise (see below)
Data exclusions	No data were excluded from the analyses.
Reproducibility	We cover two sampling stations at the Lomonosov Ridge in the Central Arctic Ocean with similar results and three sampling stations on the Morris Jesup Rise with similar results.
Randomization	Two groups were distinguished by geographical location: 2 sampling stations were on the Lomonosov Ridge in the Central Arctic Ocean Large Marine Ecosystem and 3 stations were on the Morris Jesup Rise, the continental rise north of Greenland.
Blinding	Blinding was only performed for the analysis of the species from which the otoliths originated. This was performed by two independent observers (Julek Chawarski and Kim Vane) after the expedition. After this blind test consensus was reached between the two observers.

Did the study involve field work? ☒ Yes ☐ No

Field work, collection and transport

Field conditions	Central Arctic Ocean, summer, air temperature ranged from -10 to -5 degrees Celsius, no rain but often fog.
Location	Station 26 = 150°08.330 'W. 89°07.030 'N; Station 38 = 66°24.329 'W, 87°45.084 'N; Station 48 = 33°30.226 'W, 84°55.693 'N: Station 50 = 32°28.905 'W, 84°10.092 'N, Station 53 = 24°32.521 'W, 84°31.193 'N
Access & import/export	The research conducted on the Morris Jesup Rise within the Greenland Exclusive Economic Zone complies with all relevant ethical regulations and national guidelines with permission from the Danish Ministry of Foreign Affairs, Copenhagen (JTHAV Nr. 2021-27049, August, 19th, 2021). No permission was necessary for research conducted in the high seas of the Central Arctic Ocean, as this area is outside national jurisdiction.
Disturbance	We took five 50x50x50 cm block sediment samples from a 14 million km2 area (the Arctic Ocean).

Reporting for specific materials, systems and methods

We require information from authors about some types of materials, experimental systems and methods used in many studies. Here, indicate whether each material, system or method listed is relevant to your study. If you are not sure if a list item applies to your research, read the appropriate section before selecting a response.

Materials & experimental systems

n/a	Involved in the study
☒	☐ Antibodies
☒	☐ Eukaryotic cell lines
☐	☒ Palaeontology and archaeology
☒	☐ Animals and other organisms
☒	☐ Human research participants
☒	☐ Clinical data
☒	☐ Dual use research of concern

Methods

n/a	Involved in the study
☒	☐ ChIP-seq
☒	☐ Flow cytometry
☒	☐ MRI-based neuroimaging

Palaeontology and Archaeology

Specimen provenance

No living animals were involved, only remnants (otoliths) of fish that once lived in the Arctic Ocean

Specimen deposition

The radiocarbon-dated otoliths were destroyed during laboratory processing; the remaining (parts of) the otoliths are stored at the Alfred Wegener Institute in Bremerhaven, Germany (Courtesy Hauke Flores).

Dating methods

Aging of the otoliths were performed by the 14C method according to a specific protocol that is described in detail in the paper M&M. Accelerator mass spectrometry (AMS) 14C analysis was performed at the MICADAS - Mini Carbon Dating System laboratory at the Alfred Wegener Institute Helmholtz Center for Polar and Marine Research. The analysis used a carbonate handling system and a gas interface system connected to a Mini Carbon Dating System (CHS-GIS-MICADAS), following a standard operating procedure (Mollenhauer, G., Grotheer, H., Gentz, T., Bonk, E. & Hefter, J. Standard operation procedures and performance of the MICADAS radiocarbon laboratory at Alfred Wegener Institute (AWI), Germany. Nuclear Instruments and Methods in Physics Research B 496, 45–51 (2021)). The software OxCal (version 4.4) was used to calibrate radiocarbon ages to calendar ages (Bronk Ramsey, C. Bayesian analysis of radiocarbon dates. Radiocarbon 51, 337–360 (2009)), using the Marine20 calibration curve (Heaton, T. J. et al. Marine20 – The marine radiocarbon age calibration curve (0–55,000 cal BP). Radiocarbon 62, 779–820 (2020)).

☒ Tick this box to confirm that the raw and calibrated dates are available in the paper or in Supplementary Information.

Ethics oversight

No ethical approval was required because we worked with remnants of organisms that died a long time ago. The research conducted on the Morris Jesup Rise within the Greenland Exclusive Economic Zone complies with all relevant ethical regulations and national guidelines with permission from the Danish Ministry of Foreign Affairs, Copenhagen (JTHAV Nr. 2021-27049, August, 19th, 2021). None of the samples fall within the Nagoya agreement (Greenland is not a member).

Note that full information on the approval of the study protocol must also be provided in the manuscript.
