# Supplementary material for: Survival and divergence of Arctic endemic cod species during 50,000 years of climate change
Source: Nat Commun. 2026 Oct 16;17:10876. doi: 10.1038/s41467-026-78810-4 (PMC13675226; doi:10.1038/s41467-026-78810-4)
Supplement: Supplementary file 4 [file 41467_2026_78810_MOESM8_ESM.pdf]

nature portfolio

# Peer Review File

# Survival and divergence of Arctic endemic cod species during 50,000 years of climate change

Corresponding Author: Professor Pauline Snoeijs-Leijonmalm

**This file contains all reviewer reports in order by version, followed by all author rebuttals in order by version. Parts of this Peer Review File have been redacted as indicated to remove third-party material.**

Version 0:

Reviewer comments:

Reviewer #1

(Remarks to the Author)
I really liked the intent of this paper. The use of deep-sea sediment core layers to test for temporal trends in fish diversity and abundance under the Arctic ice is an intriguing idea. Although otoliths recovered from sediment cores have been reported (rarely) before, their use under semi-permanent sea ice is an elegant solution to very challenging fish sampling logistics. That being said, I have several concerns about the design and interpretation of this study which should be addressed before any consideration of publication. In particular, I was concerned by the very low otolith numbers (often ≤2) in most of the sediment strata, which made the probability of zero counts in some strata more of a statistical inevitability than the evidence of biological absence suggested by the authors. Perhaps more concerning was the presence of otoliths in all sediment strata, in contrast with the radiocarbon dating indicated that half of the time (25,000 years), there were no fish. The apparent absence of intermediate dates seems more compatible with sediment mixing within strata or radiocarbon dating issues then true absence. Indeed, in the only two stations where there were more than 10 radiocarbon dates, the 40,000+ yr BP dates stretched through 90% of the core depth. Finally, there was no verification of the species identity of the otoliths, even though such would have been easy using fresh specimens. The statistical analysis of the otolith morphometrics was incorrect, and thus not at all reliable in supporting the visual identification.

The morphometric analysis and verification of species identity should be easy to remedy in a revision, but I'm not at all certain that the poor correlation between sediment depth and radiocarbon age can be addressed. I guess we'll have to see what the authors come up with there. Specific comments follow:

Line 33: Are fish sentient? That is arguable and certainly not well accepted. I suggest using a different word here.

Line 53 and throughout MS: The word "codfish" may be a valid word in informal writings, but the scientific literature only uses the word "cod". Using "codfish" throughout the MS will immediately cast the paper in a negative light to any reader familiar with fish or fisheries.

Line 62: The previous section states that the two fish species are highly adapted to extreme cold, but this sentence questions whether the environment during the last Ice Age might have been too extreme. We need some clarity here. What is the current thermal range for these fish species? State if they have any known minimum temperature tolerance. If not, please clarify what environmental extreme you're talking about.

Line 96: There is some circular reasoning here. The authors used their own judgement in identifying the species for each of the otoliths, and then used the same otoliths to classify them using ordination methods. If the authors erred in their initial identifications, the interpretation of the ordination results would also be wrong. What is required here is a set of otoliths of known species identity. Even otolith experts test their identification accuracy using otoliths extracted from fish of known species identity. No such test is reported here, leaving the reader uncertain if the authors are sufficiently skilled in otolith identification. Since fresh specimens of Arctic fish species are readily available, there is no reason why this test of accuracy could not be done, and the results reported. The accuracy of both the authors' otolith morphometric identifications and the ordination should be tested.

Fig 1 would benefit from some minor changes: 1) add place names to the land masses visible at the bottom left of the map; 2) indicate the age range of each of the sediment depths, either as min-max or a secondary axis; 3) indicate in the figure

caption if the 131 otoliths are all species, or only gadoids.

Line 117: The authors state that there is a general correlation between otolith radiocarbon date and sediment age, but that statement is misleading if not inaccurate. Fig. 2 shows that there is a cluster of dates <10 ka BP in the upper 3 cm of sediment, with no correlation therein. There is also another cluster of dates around 35-45 ka in the lower 12 cm, and again with no correlation within. In the intervening age period, there are only 2 dated otoliths: one at 20 ka and one at 30 ka BP. This suggests to me that much of the sediment column has been mixed up within (but not necessarily across) the major sediment layers. Indeed, in the only two stations (26 and 38) where there were more than 10 radiocarbon dates, the 40,000+ yr BP dates stretched through 90% of the core depth, from 2-15 cm (the end), with absolutely no correlation between age and core depth. The authors need to explain this, since this bears on the validity of the conclusions.

Line 166: The MS states that otoliths from the two gadoid species were absent from the Lomonosov Ridge for 25,000 years. However, the depth panel for Station 26 in Fig 1 shows that otoliths were recovered from every sediment stratum, with no gaps. Most of the other stations either lack otoliths in the lower half of the core, or show declining numbers from the surface, until there are vanishingly small sample sizes. Looking at Fig 2, it appears that the authors have interpreted the absence of radiocarbon dates between 10 and 35 ka BP as evidence of lack of fish. But if that’s the case, then the Fig 1 Station 26 panel demonstrates that there is no correlation between sediment depth and radiocarbon age (as per my comment on Line 117). In addition, the supposed absence of fish for 25,000 years could simply be an artifact of very low sample sizes. With the exception of the upper 2 cm of each core, otolith sample sizes are ≤3 for almost all depths, making zero counts a statistical inevitability, not necessarily a biological reality. These are serious issues, and need to be addressed. Otherwise, the conclusions of this MS become invalid.

Line 204: There is absolutely no basis for suggesting reduced predation or competition as a source of larger otoliths. The suggestion for a reduced mortality rate is possible, but the most likely explanation is an enhanced dissolution rate, which would preferentially dissolve the otoliths with the highest surface area to volume ratio (ie- the smallest otoliths). After all, large fish cannot exist without having also existed as smaller fish, yet the smaller fish are not evident in some of the samples. Such should be acknowledged in the MS. It would be interesting to correlate minimum otolith size with the various glacial regimes, to see if warmer conditions were most associated with larger otoliths.

Line 246: Did I misunderstand something along the way? The authors argued that there was a 25,000 year absence of all fish species in the high Arctic due to extreme glaciation. Yet here they argue that it was not a glacial desert. The two statements seem to be incompatible. Is it not possible that during the extreme glaciation events, that the two species existed only in suitable microhabitats in deep water, and then re-colonized the central basin when the temperatures warmed?

Line 512: The Methods state that otolith identification was based on Mecklenburg et al (2018), but that book only deals with fish identification, not otoliths. Which volumes were used in otolith identification?

Line 573: I don’t know what you mean by “elaborated”

Line 586: Given that the outer leachate was much older than the inner otolith core, isn’t it possible that the leaching process was asymmetric around the otolith, as is commonly observed? If so, some of the regions affected by diagenesis may not have been removed. Were any sequential leach samples investigated to test your assumption that the core was pristine? Contaminated cores might well explain the poor correspondence between sediment depth and radiocarbon age.

Line 832: The rationale for the otolith identification ordination is very strange. Why would you use PCA to analyze otoliths of unconfirmed species identity, rather than first analyze otoliths from fresh specimens of known identity? Why did you not use a discriminant analysis for classification, and then jackknife the classification to get an unbiased measure of classification accuracy? And most importantly, why did you include otolith area, perimeter and axis length in the PCA, when they are all highly correlated with fish length? You cannot put a covariate into a PCA; if you do, all you do is develop a classification tool that helps you tell large otoliths from small otoliths. The standard approach is to detrend the features that are correlated with fish length, then introduce the detrended variables into the PCA. As it stands, this PCA appears to be completely inappropriate and misleading.

Extended Data Fig 7: The reconstructed fork lengths are more consistent with otolith dissolution, ecological size segregation or size-biased detection in the sediment core, than enhanced growth rate or maximum length. Both species typically mature at more than 18-20 cm, so it is plausible that some samples/dates are associated with periods of very small maturation and maximum lengths. But if there are otoliths present from large fish, then smaller individuals must have been present too. Extended Data Fig 7b shows that fish as small as 8 cm were detected in MIS 3, yet no fish less than 18 cm were identified in Fig 7a MIS 3. Since the absence of young fish is biologically impossible, the smaller otoliths were either not present in nature (rejecting any hypotheses about mortality) or, more likely, preferentially dissolved due to their higher surface area: volume ratio. With dissolution having been demonstrated in one era, who is to say that it didn’t occur at slightly higher rates in the periods with missing otoliths?

Steven Campana

Reviewer #2

(Remarks to the Author)
I read the paper by Chawarski et al on the codfish presence in the Arctic in the last 50 ky with great interest, and indeed this

is an excellent work and a very significant contribution in my opinion. I found that the results are indeed very significant and the paper is well-written. This work I believe will form a significant contribution to the field, particularly because it demonstrates once more the great value of using fish otoliths obtained from sediment cores to understand the geohistorical evolution of fish populations. The methodology is sound and I only added very minor comments directly on the pdf. I very much look forward to seeing this paper published.

Reviewer #3

(Remarks to the Author)
Thank you for the opportunity to review this paper, I read it with great interest. Chawarski et al. present a very exciting analysis of long-term distribution of cod fishes using otolith morphometrics. The approach is ambitious, thorough and yields important insights into the past and potential future distribution of cold-adapted fish. This paper offers key knowledge for current management efforts under the rising climate change. I only have some minor comments/suggestions that I would like the authors to consider.

Line 60. Be more precise in what are "these adaptations" for both fish species.
Line 63. Is it possible to find any other fish bone in the sediment core? Why not using those too? Consider adding a very small statement of why exactly otoliths are preferred to investigate past environmental conditions and fish movement.
14C dating samples can always be problematic, but could the broad range in the age of some samples be related with the ΔR values used for the marine reservoir correction? You used the same value for both fishes, but shouldn't we use different values given the sampling locations and/or the feeding grounds/diet specificity for each fish? Could the selected ΔR value potentially affect the distributional range (in years) given in Figure 3?
Line 143: misspelling of "Further".
I found the presence/absence of polar cod very interesting, specially because there seems to be a trend in "higher presence of polar cod" with "low presence of ice cod". Do these two fish species interact/compete? From your data here (Line 191), it seems likely to be looking to "isolated populations" of ice cod as mentioned in the text. But what about (predator/prey) competition interactions specially during the MIS3 and MIS1? Ecological dynamics will generally direct distributional patterns of different species. It is not clear from your text whether one species outcompeted the other under certain conditions.
Line 521. Spell out the name of the Greenland cod (Gadus ogac).

Version 1:

Reviewer comments:

Reviewer #2

(Remarks to the Author)
I appreciate the efforts made by the authors to address my previous comments on the manuscript. However, I still have some reservations, particularly about the identification of the otoliths.

1) In response to my previouse comment that "Since otoliths are formed throughout the lifetime of the fish, these radiocarbon ages would represent the times when the fishes "lived", not died." the authors reponded that "To be most accurate, we chose to use the term 'died' because the radiocarbon "clock" starts ticking when 14C stops being replenished, i.e. upon death. We recommend keeping the text as is."
This is not correct. What you analyzed was the carbon in the aragonite of the otoliths which is deposited throughout the lifetime of the fish and not replenished. Every day, a new increment of aragonite crystallites is deposited around the previous ones.

2) In response to my comment "How were the morphological features decided?", the authors responded that "We used photographic images of our otoliths to derive two-dimensional features. After checking for covariance among features, these were the remaining two-dimensional features we could compute using Fiji software."
This does not answer my question. There is very abundant literature on this, such as the pivotal works of Nolf (1985, Otolith Piscium; and 2013) or several works by Werner Schwarzhans. I am surprised that the authors did not consider these at all. Furthermore, otoliths that end up in sediments go through significant taphonomic process, particularly some erosion of the outer surface, but also occasional breaking, and other modifications (see Agiadi et al. 2022; Paleobiology). Therefore, it is vital that the features used to identify them are clearly defined, even more so than for otoliths extracted from live-collected fish.
This reservation is further reinforced by the authors' answer to my next comment "Does "Area" refer to the inner face of the otoliths?", to which they replied "Yes, but as these otoliths lack concavity, we assume the two-dimensional area of the otolith is identical on both sides."
Absolutely not! Otoliths (of all species) do not have identical inner and outer faces! The inner face is the one characterized by the sulcus acusticus, which is a depening where the sensory macula of the inner ear are attached. It has a clear functional role, and it the most characteristic identifying feature of otoliths.

Without properly addressing the issue of otolith identification, I cannot recommend accepting the manuscript.

Reviewer #3

(Remarks to the Author)
Thank you to the authors for the thoughtful evaluation of the revisions. All my comments have been successfully addressed. I have no additional comments/feedback.

Reviewer #4

(Remarks to the Author)
General comments:
This manuscript is interesting and presents a rare and potentially high-impact dataset. The approach is original and, in my view, aligns well with current efforts to push sedimentary fish archives (otolith-based records) towards broader palaeoecological and climate-relevance questions. My suggestions are primarily intended to 1. streamline and refocus the Results/Discussion, and 2. improve the otolith-specific accuracy and citations, as several statements and references currently read as coming from a non-specialist otolith perspective.

General and major suggestions
1) on the morphometrics and identification: I appreciate the authors' effort to make identifications more objective using morphometric analyses, especially given that the authors are not primarily otolith specialists. However, the morphometrics currently occupy a disproportionate fraction of the manuscript (it is important, but currently over-emphasised in the main text) In my opinion, the otoliths of the two species are readily distinguishable, and the highlighted feature (aspect ratio) is visually obvious. Based on the figures and descriptions provided, I consider the identifications to be credible. Therefore, I do not think it is necessary to present the morphometric analyses at such length in the main manuscript.

Related to your methodology: if the main objective is to separate two species, then a supervised discriminant framework (e.g., LDA or another classifier with cross-validation) is generally more appropriate than PCA, which is exploratory/unsupervised. This aligns with Reviewer #1's view. My reading is that you selected PCA primarily to visualise and corroborate an a priori observed feature (i.e., aspect ratio) rather than to serve as a formal classification tool. If so, PCA can be retained, but the manuscript should explicitly clarify this intent and avoid presenting PCA as an "objective validation" of identifications. That said, one important nuance about what the PCA is being used to claim is needed. If feasible, I recommend adding a supervised classification analysis (or at least reporting classification accuracy with an appropriate validation scheme) as the primary quantitative support for species separation, with PCA presented as supplementary, exploratory context.

2) For the chronology and sediment mixing, I think the "two time-layer" framing is reasonable, but this has to be kept clear and more concise. I appreciate that the authors have tried their best to resolve the age issue and possible sediment mixing. In our experience, minor to moderate mixing can indeed occur (see also Lin et al. 2019 for a reefal example and discussion). I also think the authors have responded reasonably by clarifying that the material can be broadly divided into two time-layers following Reviewer #1's view, which I aalso think is a critical clarification.
My suggestion is mainly about presentation: keep this interpretation front-and-centre and ensure the manuscript does not inadvertently drift back into an implied continuous age–depth narrative. A short schematic could help (even in the Supplement).

3) At the moment parts of the results read unfocused relative to the key findings, so that it reads methodologically heavier than necessary in the main narrative. The manuscript would benefit from sharper writing and a clearer hierarchy of what is central versus ancillary. Especially the sections titled "Otoliths from Arctic endemic cod species" and "Gadoid otoliths and climate over the past 50,000 years" are currently distracting compared with the most important findings. For example, the PCA results read as diffusing the narrative; it is written partly as "validation," partly as "exploration," partly as "methods defence." That blend tends to bloat text.
In Lines 123–140 include marginal details that could be moved to the Supplementary Information without loss of scientific value.
I strongly encourage a restructure so the main text concentrates on the most important biological/climate conclusions, with methodological depth placed in the Supplement.

4) Dissolution problem. I agree that dissolution of otoliths, particularly affecting smaller specimens is not likely to be the primary driver here, given repeated discoveries of small otoliths. Previous actuo-palaeontological works also show that dissolved otoliths can typically be distinguished from intact specimens (e.g., Schwarzhans 2013; Lin et al. 2016, 2017). However, I suggest adding the ACD/lysocline context for the region (and explicitly relating your station depths to it) would make the depth-related carbonate dissolution argument much clearer.

**Lin, C.-H. et al., 2016. Fish otolith assemblages from Recent NE Atlantic sea bottoms: A comparative study of palaeoecology. Palaeogeography, Palaeoclimatology, Palaeoecology 446: 98–107.
**Lin, C.-H. et al., 2017. Fish otoliths in superficial sediments of the Mediterranean Sea. Palaeogeography, Palaeoclimatology, Palaeoecology 471: 134–143.
**Schwarzhans, W., 2013. Otoliths from dredges in the Gulf of Guinea and off the Azores – an actuo-paleontological case study. Palaeo-Ichthyologica 13, 7–40.

Additional comments on otolith-specific accuracy
5) Line ~62: Please revise the statement about otolith resistance and chemical composition. Your revised text is incorrect. The statement that otoliths are more resistant to degradation than skeletal elements because of their chemical composition is not correct. Otoliths are aragonitic and can be vulnerable under lower pH settings, whereas phosphatic remains (teeth,

vertebrae) are often more robustly preserved. In many oceanic cores, otoliths may be absent while vertebrae/teeth are abundant (see e.g., Salvatteci et al. 2022; Sibert et al.).
The fundamental advantage of otoliths is instead their taxonomic value and often high representation (when preserved), rather than a blanket claim of superior resistance. In my view, otoliths, teeth, and other fish remains are complementary proxies, each with different preservation biases and strengths, rather than one being "better" than the others.

6) Following the above, citations around deep-sea otolith applications need correction
Jones & Checkley (2019) is appropriate. However: Salvatteci et al. (2022) did not use otoliths (as cited here); and the setting and focus of Agiadi et al. (as currently cited) do not align with the deep-sea sediment-core otolith context you are invoking. Because otolith studies from deep-sea cores remain relatively rare (and novel), the literature selection here matters. More appropriate examples to cite in this context include Lin et al. (2023) and Pallacks et al. (2025) (both explicitly demonstrate downcore otolith records used for palaeoecological inference).

**Pallacks, et al., 2025. Ocean deoxygenation linked to ancient mesopelagic fish decline. Communications Earth & Environment 6: 596.
**Lin et al., 2023. Ocean temperature drove changes in the mesopelagic fish community at the edge of the Pacific Warm Pool over the past 460,000 years. Science Advances 9: 27, eadf0656.

7) Lines ~96, 102, 111 and Supplementary Fig. 1: Please revise standard otolith terminology: Use otolith length (OL) rather than "X mm long"; Use length and height (not "length and width") for primary dimensions, and ensure consistency throughout (including Supplementary figures).

8) Line ~137: The acid-leaching approach and use of inner material for radiocarbon dating is reasonable. As an optional improvement, micro-Raman spectroscopy can provide a rapid check of mineralogy (aragonite vs alteration products) to support your diagenesis discussion.

9) Line ~350: The illustrations in Nolf & Steurbaut (1989c; fig. 8) include otoliths of both species and support your identifications. I recommend explicitly citing this source (and, if feasible, using it as a comparative reference figure in the Supplement).

10) Line ~352: revise awkward citations, these are non-gadid sources. Agiadi et al. (your ref. #20) and Schwarzhans (#76) do not deal with gadids in the way needed here. The most relevant citations for gadid otolith morphology and comparative framework should be Nolf & Steurbaut (1989a, b, c).

**Nolf, D. & Steurbaut, E., 1989a. Evidence from otoliths for establishing relationships between gadiforms and other groups. In: Cohen, D.M. (ed.), Papers on the systematics of gadiform fishes. Science Series, Natural History Museum of Los Angeles County, 32: 37–45.
**Nolf, D. & Steurbaut, E., 1989b. Importance and restrictions of the otolith-based fossil record of gadiform and ophidiiform fishes. In: Cohen, D.M. (ed.), Papers on the systematics of gadiform fishes. Science Series, Natural History Museum of Los Angeles County, 32: 47–58.
**Nolf, D. & Steurbaut, E., 1989c. Evidence from otoliths for establishing relationships within gadiforms. In: Cohen, D.M. (ed.), Papers on the systematics of gadiform fishes. Science Series, Natural History Museum of Los Angeles County, 32: 89–111.

11) Line ~353 and Supplementary Fig. 1: plural of "colliculum"
The plural of colliculum should be collicula (not "colliculi"). Please revise consistently in text and figures.

12) Figure 1 legend. In the Figure 1 legend, "Stations 26 and 38" are listed on Lomonosov Ridge and "Stations 38, 50, and 53" on Morris Jesup Rise. The second "38" appears to be a typo

13) Supplementary Figs. 1 & 3. The otolith face orientation needs improvement; it appears the authors misunderstood the basic otolith orientation. The face bearing the sulcus is the medial/inner face, and the opposite is the lateral/outer face. Please revise these figures and thoroughly check the text.

Version 2:

Reviewer comments:

Reviewer #4

(Remarks to the Author)
I believe the authors have revised their manuscript thoroughly. All my comments have been successfully addressed, and I have no additional comments.

**Open Access** This Peer Review File is licensed under a Creative Commons Attribution 4.0 International License, which permits use, sharing, adaptation, distribution and reproduction in any medium or format, as long as you give appropriate credit to the original author(s) and the source, provide a link to the Creative Commons license, and indicate if changes were made.
In cases where reviewers are anonymous, credit should be given to 'Anonymous Referee' and the source.
The images or other third party material in this Peer Review File are included in the article's Creative Commons license, unless indicated otherwise in a credit line to the material. If material is not included in the article's Creative Commons license and your intended use is not permitted by statutory regulation or exceeds the permitted use, you will need to obtain permission directly from the copyright holder.
To view a copy of this license, visit https://creativecommons.org/licenses/by/4.0/

**REVIEWER COMMENTS and OLD TEXT are in BLACK**
**AUTHOR RESPONSES are in BLUE**
**NEW TEXT is in red**

**Reviewer #1 – General comments**

I really liked the intent of this paper. The use of deep-sea sediment core layers to test for temporal trends in fish diversity and abundance under the Arctic ice is an intriguing idea. Although otoliths recovered from sediment cores have been reported (rarely) before, their use under semi-permanent sea ice is an elegant solution to very challenging fish sampling logistics. That being said, I have several concerns about the design and interpretation of this study which should be addressed before any consideration of publication.
AUTHOR RESPONSE: We are glad to hear that Reviewer #1 finds our research focus intriguing, and we appreciate his thorough review, which significantly helped us improve the manuscript. Below, we address the reviewer's concerns about our study design and interpretation, and provide a detailed explanation of the changes made in the resubmitted manuscript to address these concerns.

Reviewer #1, General comment: In particular, I was concerned by the very low otolith numbers (often ≤2) in most of the sediment strata, which made the probability of zero counts in some strata more of a statistical inevitability than the evidence of biological absence suggested by the authors.
AUTHOR RESPONSE: We agree that the number of otoliths we recovered from the sediments was low, especially on the Morris Jesup Rise, and that the absence of otoliths does not necessarily imply biological absence. We now stress in the text that otoliths were rare in our samples – in fact, we were fortunate to find them in an area with such low productivity over the past 50,000 years. During the icebreaker expedition, it was logistically impossible to collect more box core samples. We only mention the absence of otoliths in the period 35-12 ka BP for the Lomonosov Ridge (Central Arctic Ocean), which is a very long time span (half the 50,000 years covered in this paper) during which otoliths did occur on the Morris Jesup Rise (northern Greenland shelf) but not on the Lomonosov Ridge. However, it is more accurate to divide the total number of otoliths found at a sampling station by the total sediment volume (across all strata) at that station, as is now done.

TEXT REPLACED (Lines 78-79 in the originally submitted ms):
Average otolith density varied between 1.4 and 7.7 otoliths per $dm^{-3}$ sediment.
TEXT REPLACEMENT (Lines 77-78 in the resubmitted ms):
Otoliths were rare in the sediment samples, with densities ranging from 1.0 (Station 50) to 6.4 (Station 26) otoliths per cubic decimeter of sediment (Supplementary Data 2).
Please note that the numbers in Lines 77-78 have been slightly adjusted because previously, the average of the 11 strata per sampling station was reported.

TEXT REPLACED (Lines 491-493 in the originally submitted ms - Methods):
Otoliths were rare in the sediments, and it was necessary to sieve larger sediment volumes than is usual for studying protist remains, such as foraminifers[69] or meiofauna such as ostracods[40].
TEXT REPLACEMENT (Lines 324-328 in the resubmitted ms - Methods):
Otoliths were rare in our sediment samples, and absent from 19 of the 55 strata collected at the five sampling stations, even though we sieved considerably larger sediment volumes from the stratified samples (9 cubic decimeters per station) than are typically used for studying remains of smaller organisms, such as foraminifers[74] and ostracods[45].

TEXT REPLACED (Lines 499-501 in the originally submitted ms - Methods):
To maximize the yield of otoliths, the 0-15 cm deep sediment remaining in-between the push cores (including push cores taken for other research projects) was also sampled as much as possible.
TEXT REPLACEMENT (Lines 330-334 in the resubmitted ms - Methods):
Due to the rarity of the otoliths, we maximized the yield per sampling station by also collecting as many otoliths as possible from the 0–15 cm deep sediment remaining between the push cores (including those gathered for other research projects). These otoliths could not be used for analyzing stratification, but they could be used for species identification, morphometric measurements, and radiocarbon dating.

Reviewer #1, General comment: Perhaps more concerning was the presence of otoliths in all sediment strata, in contrast with the radiocarbon dating indicated that half of the time (25,000 years), there were no fish. The apparent absence of intermediate dates seems more compatible with sediment mixing within strata or radiocarbon dating issues then true absence. Indeed, in the only two stations where there were more than 10 radiocarbon dates, the 40,000+ yr BP dates stretched through 90% of the core depth.
AUTHOR RESPONSE: On the western Lomonosov Ridge (Central Arctic Ocean), where otoliths were not recorded for about 25,000 years, otoliths were actually recovered from nearly all strata (20 out of 22). We have now clearly stated that there is no linear relationship between sediment depth and otolith radiocarbon age, but rather two layers of different otolith radiocarbon ages with probable bioturbation within the layers. The reviewer elsewhere correctly pointed out the clear clustering of the otolith radiocarbon ages into two sediment layers with a sharp boundary, as well as potential sediment mixing within the two layers. We acknowledge that a more detailed and accurate explanatory text was needed here, including references to relevant literature. We have now completely rephrased the paragraph addressing this issue. Contextual paleoclimatic evidence provides a mechanism to explain this finding (intense ice cover, little to no primary production, hardly any sedimentation), lending credibility to the proposal of severely slowed or halted fish production during these 25,000 years on the Lomonosov Ridge. We also agree that the absence of otoliths within the sediment record for 25,000 years on the Lomonosov Ridge (Central Arctic Ocean) does not necessarily imply the complete absence of fish production during that time. Therefore, we have now better emphasized the difference between the absence of otoliths (observation) and their interpretation (slowed or halted production of fish) in the text. It is also important to note that the pattern of 25,000 years of absence was replicated at both sampling stations in the Central Arctic Ocean, separated by hundreds of kilometers from each other, thereby strengthening our conclusion.

TEXT REPLACED (Lines 117-125 in the originally submitted ms – Main text):
As expected, otolith-sample age generally increased with increasing sediment depth (Fig. 2). The radiocarbon results showed that the otoliths in the upper 3-4 cm were deposited during the Holocene, which was characterized by a distinct sediment lithology comprising fine-grained, brown-colored, clayey silt rich in manganese with a minor sand component. Below this layer, there was a transition to glacial sediments with coarser-grained lighter-colored sandy silt rich in calcium (Fig. 2). Exceptions to this pattern were two >40,000 years old otoliths recovered from the upper 0-2 centimeters of the Lomonosov Ridge cores and one otolith of about 20,000 years old from the Morris Jesup Rise (Fig. 2), which were possibly displaced to the surface by bioturbation.
TEXT REPLACEMENT (Lines 141-160 in the resubmitted ms – Main text):
There was no linear correlation between sediment depth and the radiocarbon age of the otoliths. Instead, two distinct layers indicated two separate time periods: otoliths younger than 12,000 years (Holocene) in the darker-brown top 0 to ~5 cm sediment layer, and otoliths older than 35,000 years in the lighter-colored (calcium-enriched) lower ~5 to 15 cm sediment layer (Fig. 3; Supplementary Data 4). Exceptions to this pattern included two otoliths older than 40,000 years recovered from the top 0 to 2 cm of the Lomonosov Ridge cores, which might have been displaced by bioturbation between the two layers. Besides these exceptions, our data suggest that sediment mixing (bioturbation) likely occurred within each of the two layers. These observations could be explained by changes in sedimentation and bioturbation rates over time. One possibility is that sedimentation rates during the final stage of the last ice age, but before the LGM, were relatively high for a short period of time[31], which might explain the apparent clustering and the larger sediment layer interval (5–15 cm) with otoliths older than 35,000 years. Subsequently, as the climate cooled toward the peak of the LGM, primary productivity, sedimentation rates, and activity of benthic macrofauna were essentially halted in the Central Arctic Ocean beneath very thick perennial ice, as previously suggested[3,32]. The LGM period would thus be a very condensed horizon (<1 cm) in our records, or possibly a hiatus[7,31,33,35,36], corresponding to the lithological boundary observed around 5 cm of sediment depth (Fig. 3). Thereafter, sedimentation rates would have returned to the more typical, relatively low interglacial levels, accompanied by bioturbation in the top 0–5 cm of sediment[23,30,34,35,36], thus mixing Holocene-aged otoliths within the Holocene layer.

The following six references have been added to the manuscript to support the above discussion on stratigraphy and radiocarbon ages:


31. Hanslik, D. et al. Quaternary Arctic Ocean sea ice variations and radiocarbon reservoir age corrections. *Quaternary Science Reviews* **29**, 3430–3441 (2010).
32. Stein, et al. A 430 kyr record of ice-sheet dynamics and organic-carbon burial in the central Eurasian Arctic Ocean. *Nature Communications* **16**, 3822 (2025).
33. Polyak, L. et al. Late Quaternary stratigraphy and sedimentation patterns in the western Arctic Ocean. *Global and Planetary Change* **68**, 5–17 (2009).
34. Backman, J., Jakobsson, M., Løvlie, R., Polyak, L., Febo, L. A. Is the central Arctic Ocean a sediment starved basin? *Quaternary Science Reviews* **23**, 1435-1454 (2004).
35. Löwemark, L., Singh, A. Influence of deep-reaching bioturbation on Arctic Ocean radiocarbon chronology. *Communications Earth & Environment* **5**, 293 (2024).

36. Wollenburg, J. et al. Reply to: Influence of deep-reaching bioturbation on Arctic ocean radiocarbon chronology. *Communications Earth & Environment* **5**, 303 (2024).

TEXT REPLACED (Lines 165-170 in the originally submitted ms – Main text):
Accordingly, an about 25,000 year long absence of *A. glacialis* and *B. saida* otoliths in our sediment record on the western Lomonosov Ridge towards the end of MIS 3 and throughout MIS 2 (Fig. 3) suggests that biological productivity became too low to sustain gadoid fish stocks under the severe ice conditions of the LGM, involving thick perennial sea ice over much of the Arctic Ocean, and ice shelves protruding from the Arctic continental margins potentially hundreds of kilometers offshore[43].
TEXT REPLACEMENT (Lines 202-214 in the resubmitted ms – Main text):
Consistent with these changes, our sediment records from the western Lomonosov Ridge (Stations 26 and 38, separated by hundreds of kilometers) show an approximately 25,000–year absence of *A. glacialis* and *B. saida* otoliths towards the end of MIS 3 and throughout MIS 2 (Fig. 4a,b). Out of the 44 radiocarbon-dated otoliths from these two stations, 25 are older than 35,000 years, 19 are younger than 12,000 years, and none are from the period between 12 and 35 ka BP. These data suggest that, during the LGM, biological productivity in the Central Arctic Ocean dropped below levels needed to sustain significant gadoid fish populations, coinciding with thick perennial sea ice across much of the deep basins and ice shelves extending potentially hundreds of kilometers from the coasts[48]. This interpretation aligns with other proxies indicating extensive perennial ice and near-zero biological productivity in the Central Arctic Ocean at that time[3,6,45]. The Greenland, Laurentide, and Fennoscandian ice sheets reached their maximum extents in the past 50,000 years during the LGM[4,49], and even the Nordic Seas were largely ice-covered with low net primary productivity (Fig. 4g,h)[41,50].

Reviewer #1, General comment: Finally, there was no verification of the species identity of the otoliths, even though such would have been easy using fresh specimens.
AUTHOR RESPONSE: We agree that species identification would have been stronger if we had had data available to build a classification model that included otoliths from modern *A. glacialis* and *B. saida*. However, we are fully confident with our identifications. We believe accurate identification of gadoid otolith to the species level requires careful microscopic inspection of three-dimensional microstructures, including concavity, crenulation, and sulcus shape (as shown in Extended Data Fig. 1). During the inception of this study, we considered an approach using otoliths from fresh specimens in our analysis and we contacted various researchers and institutions to obtain otoliths from *A. glacialis*. While we have our own extensive collection of *B. saida* otoliths at the Alfred Wegener Institute (AWI), colleagues we contacted in Greenland, Iceland, the USA, Norway, and Russia were unable to provide otoliths from *A. glacialis* due to their infrequent identification/occurrence in fisheries sampling surveys. Given these circumstances, we intensively used the AWI otolith collection to verify the identification of otoliths from *B. saida*, and further relied on published images and descriptions of both species. Moreover, due to diagenetic processes that affect otolith morphology, we assigned confidence scores to illustrate our caution in species assignments to each otolith (see “Methods”). Furthermore, our conclusions regarding the differences in patterns between the two species, specifically the peak of *A. glacialis* during the LGM at 19-21 ka, align with our highest confidence scores (4-5).

NEWLY ADDED TEXT (Lines 359-363 in the resubmitted ms - Methods)
To optimize species identification, we also compared our otoliths with the extensive otolith collection from *B. saida* available at the Alfred Wegener Institute (AWI), in Bremerhaven, Germany. For *A. glacialis*, we had to rely solely on the identification literature because we were unable to acquire otoliths from recent collections, as this species is rarely caught in fisheries sampling surveys.

Reviewer #1, General comment: The statistical analysis of the otolith morphometrics was incorrect, and thus not at all reliable in supporting the visual identification.
AUTHOR RESPONSE: We do not believe that the PCA is incorrect. Here, we summarize all our responses to the PCA statistical analysis and how we clarified the text. The PCA was performed using four two-dimensional morphometric features, and no other features were included in the statistical analysis. Later, we visualized our earlier species identifications, confidence scores, and otolith lengths in the ordination plot to discuss whether two-dimensional morphometric data (in particular, the aspect ratio) alone could distinguish between the two species, as previously suggested in the identification literature. In another line-referenced comment (Line 832, see below), the reviewer was concerned about the collinearity of the morphometric features in the PCA. In Extended Data Fig. 4, we provide two correlation coefficient matrices: the first (A) includes all nine morphometric features measured, and the second (B) includes only the four features used in PCA, i.e., the five features with $r_s$>0.90 were removed. Furthermore, the features used in the PCA were standardized to unit variance and centered (see the R "prcomp" method in the R "stats" package). It is not mathematically incorrect to use collinear variables that are in PCA ordination, but strongly correlated variables may overemphasize particular eigenvectors. Therefore, we did not use the five features that were heavily correlated to other features in the PCA. For more clarity, the text about the statistical analysis (PCA) has been revised as follows. Additionally, the legend for Extended Data Fig. 5 has been updated.

TEXT REPLACED (Lines 86-90 in the originally submitted ms):
Altogether, 206 (80 %) of the 256 otoliths could be identified to the species level based on microscopic evaluation of their distinctive morphology, and morphometric features (Methods, Extended Data Figs 1-3). The identified otoliths originated from the two Arctic endemic codfishes (gadoids) ice cod (*A. glacialis*; 57 otoliths) and polar cod (*B. saida;* 145 otoliths), as well as snailfishes (*Paraliparis* sp.; 4 otoliths) (Extended Data Table 3).
TEXT REPLACEMENT (Lines 90-97 in the resubmitted ms):
Altogether, 206 (80%) of the 256 otoliths could be identified to the species level through microscopic evaluation of their distinctive three-dimensional microstructures, including concavity, crenulation, and sulcus shape (Supplementary Figs 1–3; Supplementary Data 3). … The identified otoliths originated from the two Arctic endemic cod species, ice cod (*A. glacialis*; 57 otoliths, 2.8–14.2 mm long) and polar cod (*B. saida;* 145 otoliths, 2.2–8.3 mm long) (Fig. 2), as well as snailfishes (*Paraliparis* sp.; 4 otoliths, 1.2–2.0 mm long).

TEXT REPLACED (Lines 92-96 in the originally submitted ms):
Correlation and Principal Component Analyses for 206 otoliths for which two-dimensional morphometric data could be obtained (*A. glacialis*, *B. saida*, 'unidentified gadoid') confirmed

that the aspect ratio (length/width) of the otoliths was the morphometric feature that contributed most to interspecific variability between the Arctic gadoid otoliths[22] (Extended Data Figs 4-6).

TEXT REPLACEMENT (Lines 100-120 in the resubmitted ms):

In one of the otolith identification keys we used, it was stated that "*A. glacialis* otoliths look similar to those of *B. saida* but can usually be distinguished by proportionately greater widths"[22], and this is also what we observed in our otoliths. Because relying solely on two-dimensional morphometric features could simplify distinguishing the two species, we tested how an analysis of only two-dimensional morphometric features aligned with (1) our careful visual otolith identifications, (2) our estimated identification confidence scores, and (3) otolith size ranging from juvenile to old individuals. Out of nine two-dimensional morphometric features, measured or derived from 206 fully intact gadoid otoliths, five features showed strong collinearity with others (Spearman's Rank Correlation Coefficients ($r_S$) > 0.90, Supplementary Fig. 4a). A principal component analysis (PCA) using the remaining four morphometric features (area, solidity, ellipse and aspect ratio; Supplementary Fig 4b) confirmed that the aspect ratio (length to width ratio) of the otoliths was indeed the two-dimensional morphometric feature that contributed most to interspecific variability as recorded by our visual identification (Supplementary Fig. 5). A binomial logistic regression model predicted that an aspect ratio of 2.09 ± standard error 0.02 denotes the 0.5 probability level for distinguishing between the two species (Supplementary Fig. 6). However, considerable overlap in the aspect ratio ranges of the two species limits the reliability of using aspect ratio alone for species identification. For *A. glacialis*, the aspect ratio ranged from 1.84 to 2.23 (n = 49 otoliths), and for *B. saida*, it ranged from 1.88 to 3.00 (n = 129). The PCA ordination and the regression model also demonstrated that the separation between the two species, based only on two-dimensional features, was more distinct for larger otoliths than for smaller ones.

TEXT REPLACED (Lines 534-545 in the originally submitted ms – Methods):

Nine two-dimensional morphometric features were measured for assessment of the quantitative morphology of the Arctic gadoid otoliths to support the visual identification. Not all otoliths could be analyzed in this way due to damages to the otoliths; altogether, results were obtained for 206 otoliths from 99 stratified and 107 bulk sediment samples (49 *A. glacialis*, 129 *B. saida*, 28 'unidentified gadoid'). The morphological features included: (1) Area = the surface area of the otolith in $mm^2$, (2) Perimeter = the length of the outside boundary of the otolith in mm, (3) Major axis = the primary axis of the best fitting ellipse in mm, (4) Minor axis = the secondary axis of the best fitting ellipse in mm, (5) Elongation = 4*Perimeter /Area, (6) Solidity = the area of the otolith divided by its convex area (also known as 'convexity'), (7) Roundness = $(4*area)/(\pi*major\_axis^2)$, where a value of 1 indicates a circle and a values greater than 1 indicate an increasingly non-circular shape, (8) Ellipse = Major axis minus minor axis in mm, (9) Aspect ratio = the ratio between the major and the minor axis. The features were derived from photographic images, using Fiji software[77]. Since two species were involved, the data were not normally distributed as tested by the Kolmogorov-Smirnov Test of Normality and the relationships between some of the features were not linear. Spearman's Rank Correlation Coefficients ($r_S$) were calculated to assess correlations between the features between the nine morphometric features. Principal Components Analysis (PCA) implemented with the R 'stats' package[78] and 'FactoMineR'[79] was used was used to visualize multivariate patterns in the morphometric features of 206 otoliths (49 *A. glacialis*, 129 *B. saida*, 28 'unidentified gadoid')

TEXT REPLACEMENT (Lines 337-395 in the resubmitted ms – Methods):
Nine two-dimensional morphometric features were extracted from photographic images using Fiji software[82]. Data were collected from 206 fully intact gadoid otoliths (46 otoliths were damaged and could not be included). Ninety-nine intact otoliths came from stratified sediment samples, and 107 from bulk samples (49 *A. glacialis*, 129 *B. saida*, 28 unidentified gadoids). Since two species were involved, the data were not normally distributed, as confirmed by the Kolmogorov-Smirnov Test of Normality, and the relationships between some of the features were not linear. Spearman's Rank Correlation Coefficients ($r_S$) were calculated to evaluate the relationships among the nine morphometric features. Five morphometric features were excluded from further statistical analysis due to high collinearity (Spearman's Rank Correlation Coefficients ($r_S$) > 0.90), which could overemphasize specific eigenvectors in a principal component analysis (PCA). PCA, implemented with the R "stats"[83] and "FactoMineR"[84] packages, was used to visualize multivariate patterns in the morphometric features of the 206 otoliths using the four remaining morphometric features as input data: area = the surface area of the otolith in square millimeters, solidity = the area of the otolith divided by its convex area (also known as "convexity"), ellipse = the difference between the major and minor axis in millimeters, and aspect ratio = the ratio between the major and minor axis. Our species identifications, identification confidence scores, and otolith length were passively overlaid (not included in the statistical analysis) on the ordination plot to compare them with the results of the PCA based solely on the four two-dimensional morphometric features.

Reviewer #1, General comment: The morphometric analysis and verification of species identity should be easy to remedy in a revision, but I'm not at all certain that the poor correlation between sediment depth and radiocarbon age can be addressed. I guess we'll have to see what the authors come up with there.
AUTHOR RESPONSE: The morphometric analysis and verification of species identity have been addressed in response to the reviewer's comments. We now clearly discuss the poor correlation between sediment depth and radiocarbon age of the otoliths (see above) and have added five additional literature references to support our explanation.

**Reviewer #1 – Specific comments**

Reviewer #1, Line 33: Are fish sentient? That is arguable and certainly not well accepted. I suggest using a different word here.
AUTHOR RESPONSE: Point taken – "sentient" (meaning to perceive or feel things) has been replaced with "vertebrate".

Reviewer #1, Line 53 and throughout MS: The word "codfish" may be a valid word in informal writings, but the scientific literature only uses the word "cod". Using "codfish" throughout the MS will immediately cast the paper in a negative light to any reader familiar with fish or fisheries.
AUTHOR RESPONSE: We have changed "codfish" to "gadoid" in the entire manuscript. We cannot use the expression "Arctic cod" because that is used as a vernacular name for *Boreogadus*

*saida* in North America and specific populations of *Gadus morhua* in Norway. Thus, "Arctic cod" is confusing for readers from different continents.

Reviewer #1, Line 62: The previous section states that the two fish species are highly adapted to extreme cold, but this sentence questions whether the environment during the last Ice Age might have been too extreme. We need some clarity here. What is the current thermal range for these fish species? State if they have any known minimum temperature tolerance. If not, please clarify what environmental extreme you're talking about.

AUTHOR RESPONSE: We agree that the text was somewhat confusing. The thermal range of the two species (around 0 degrees Celsius) will be the same now as it was during the last ice age. However, the lack of food (ecosystem productivity) is a crucial factor for survival and is a major conclusion of our paper. The last two sentences of this paragraph have now been revised.

TEXT REPLACED (Lines 57-62 in the originally submitted ms – Main text):
These two Arctic codfishes constitute important links between the lower and higher trophic levels of the Arctic food web[12,17] and are highly adapted to cold ocean temperatures and under-ice habitats. and are highly adapted to cold ocean temperatures and under-ice habitats. Whether these adaptations enabled them to endure the harshest environmental conditions in the Arctic Polar Domain during the last ice age, is unknown.
TEXT REPLACEMENT (Lines 56-61 in the resubmitted ms – Main text):
Together, these two Arctic gadoids form key links between the lower and higher trophic levels of the contemporary Arctic food web[12,17]. However, despite their cold adaptations, such as antifreeze glycoproteins that enable them to thrive in water temperatures below 0 ºC, it remains an open question whether these fish could have survived the extreme conditions of the last ice age, when ecosystem productivity in the Arctic Polar Domain was likely much lower than it is today.

Reviewer #1, Line 96: There is some circular reasoning here. The authors used their own judgement in identifying the species for each of the otoliths, and then used the same otoliths to classify them using ordination methods. If the authors erred in their initial identifications, the interpretation of the ordination results would also be wrong. What is required here is a set of otoliths of known species identity. Even otolith experts test their identification accuracy using otoliths extracted from fish of known species identity. No such test is reported here, leaving the reader uncertain if the authors are sufficiently skilled in otolith identification. Since fresh specimens of Arctic fish species are readily available, there is no reason why this test of accuracy could not be done, and the results reported. The accuracy of both the authors' otolith morphometric identifications and the ordination should be tested.
AUTHOR RESPONSE: This matter is addressed in detail by the changes made to lines 86-90 and 92-96 (main text) and lines 534-345 (methods) of the originally submitted manuscript, see "Reviewer #1 - General comments" above for further details.

Reviewer #1, Fig 1: Figure 1 would benefit from some minor changes: 1) add place names to the land masses visible at the bottom left of the map; 2) indicate the age range of each of the sediment depths, either as min-max or a secondary axis; 3) indicate in the figure caption if the 131 otoliths are all species, or only gadoids.
AUTHOR RESPONSE: 1) The map has been modified to include the land mass names "Canada" and "Greenland"; 2) Adding the age range of sediment depth is not possible as no stratification-age model has been constructed for these cores. As mentioned above in more detail: there is no linear relationship between sediment depth and age due to variation in sedimentation processes throughout the study period, instead there are two clusters as shown in Fig. 2; 3) "(all species)" has been included in the figure legend.

Reviewer #1, Line 117: The authors state that there is a general correlation between otolith radiocarbon date and sediment age, but that statement is misleading if not inaccurate. Fig. 2 shows that there is a cluster of dates <10 ka BP in the upper 3 cm of sediment, with no correlation therein. There is also another cluster of dates around 35-45 ka in the lower 12 cm, and again with no correlation within. In the intervening age period, there are only 2 dated otoliths: one at 20 ka and one at 30 ka BP. This suggests to me that much of the sediment column has been mixed up within (but not necessarily across) the major sediment layers. Indeed, in the only two stations (26 and 38) where there were more than 10 radiocarbon dates, the 40,000+ yr BP dates stretched through 90% of the core depth, from 2-15 cm (the end), with absolutely no correlation between age and core depth. The authors need to explain this, since this bears on the validity of the conclusions.
AUTHOR RESPONSE: This matter is addressed in detail by the changes made to lines 117-125 and 165-170 (main text) of the originally submitted manuscript, see "Reviewer #1 - General comments" above for further details.

Reviewer #1, Line 166: The MS states that otoliths from the two gadoid species were absent from the Lomonosov Ridge for 25,000 years. However, the depth panel for Station 26 in Fig 1 shows that otoliths were recovered from every sediment stratum, with no gaps. Most of the other stations either lack otoliths in the lower half of the core, or show declining numbers from the surface, until there are vanishingly small sample sizes. Looking at Fig 2, it appears that the authors have interpreted the absence of radiocarbon dates between 10 and 35 ka BP as evidence of lack of fish. But if that's the case, then the Fig 1 Station 26 panel demonstrates that there is no correlation between sediment depth and radiocarbon age (as per my comment on Line 117). In addition, the supposed absence of fish for 25,000 years could simply be an artifact of very low sample sizes. With the exception of the upper 2 cm of each core, otolith sample sizes are ≤3 for almost all depths, making zero counts a statistical inevitability, not necessarily a biological reality. These are serious issues, and need to be addressed. Otherwise, the conclusions of this MS become invalid.
AUTHOR RESPONSE: As was well observed by the reviewer and addressed in previous comments, there is indeed no linear correlation between otolith age and sediment depth in the sediments. We are grateful to the reviewer for the comments about this, which made us clarify that there are two (bioturbated) clusters, separated by a condensed LGM unit or possible hiatus, as reported in the literature. This greatly improves the manuscript. This matter is addressed in

detail by the changes made to lines 17-125 and 165-170 (main text) of the originally submitted manuscript, see “Reviewer #1 - General comments” above for further explanation.

Reviewer #1, Line 204: There is absolutely no basis for suggesting reduced predation or competition as a source of larger otoliths. The suggestion for a reduced mortality rate is possible, but the most likely explanation is an enhanced dissolution rate, which would preferentially dissolve the otoliths with the highest surface area to volume ratio (i.e. the smallest otoliths). After all, large fish cannot exist without having also existed as smaller fish, yet the smaller fish are not evident in some of the samples. Such should be acknowledged in the MS. It would be interesting to correlate minimum otolith size with the various glacial regimes, to see if warmer conditions were most associated with larger otoliths.
AUTHOR RESPONSE: We found that small otoliths were challenging to identify due to a lack of distinguishing features that are clearly present in adult (larger) otoliths, and we showed as well that it is impossible to use the otolith aspect ratio alone for identification. However, e.g., about equally many radiocarbon-dated smaller otoliths of juvenile *B. saida* (22 otoliths of 2.7-4.9 mm) were found on the Morris Jessup Rise compared to larger ones of adult fish (23 otoliths of 5.2-8.4 mm), see Extended Data Table 4. The presence of small otoliths thus negates the premise that dissolution is responsible for the variation in sizes we see across each period. Fry and very small juveniles are not expected in the open ocean (our stations) since these occur in coastal hatching areas. Furthermore, the finding of the small *Paraliparis* otoliths (1-2 mm; Extended Data Fig. 3), possessing distinguishing morphological characteristics, further demonstrates that dissolution does not lead to the complete absence of small otoliths. Nonetheless, our interpretation of the reconstructed size distributions should be more distinctly confined to the size diversity and the maximum size of fish. Population measures such as size diversity and maximum size can be driven by a range of ecological processes (including predation, competition), population dynamics (including mortality and migration), and environmental conditions (including temperature and habitat structure). However, we agree that predation and competition processes are speculative drivers of otolith size distribution. Therefore, we removed mention of predation and competition here and modified the text as follows.

TEXT REPLACED (Lines 200-204 in the originally submitted ms – Main text):
A different ecosystem structure in these glacial refuges compared to the present situation is suggested by differences in the size distribution of *A. glacialis* indicated by our otolith record. Size reconstructions from otoliths (Extended Data Fig. 7) suggest a higher proportion of larger (>25 cm fork length) *A. glacialis* during MIS 3 (Lomonosov Ridge) and MIS 2 (Morris Jesup Rise) compared to MIS 1, possibly due to lower mortality or enhanced growth resulting from reduced predation pressure or competition.
TEXT REPLACEMENT (Lines 244-251 in the resubmitted ms – Main text):
Differences in the size distributions of *A. glacialis* otoliths across marine isotope stages indicate changes in ecological conditions. Fish size reconstructions from otoliths (Fig. 5) reveal a larger mean fish size of *A. glacialis* during MIS 3 compared to MIS 1 at the Lomonosov Ridge. At the Morris Jesup Rise, both mean size and size diversity of *A. glacialis* were higher during a period of increased occurrence in MIS 2 compared to MIS 3 and MIS 1. When considered alongside the absence of *B. saida* from the Morris Jesup Rise during MIS 3 and MIS 2, these patterns suggest

that changes in habitat structure contributed to the subtle yet divergent life histories of the two species we see today.

Reviewer #1, Line 246: Did I misunderstand something along the way? The authors argued that there was a 25,000 year absence of all fish species in the high Arctic due to extreme glaciation. Yet here they argue that it was not a glacial desert. The two statements seem to be incompatible. Is it not possible that during the extreme glaciation events, that the two species existed only in suitable microhabitats in deep water, and then re-colonized the central basin when the temperatures warmed?
AUTHOR RESPONSE: We believe a misunderstanding happened here. We interpret our results as indicating the climatological variation during the ice age, which led to temporary periods of fish presence in the high Arctic. We hypothesize that *A. glacialis* found an isolated refuge at or near polynyas along the edge of the Greenland ice sheet, close to the Atlantic gateway. During this same period, *B. saida* likely retreated to lower latitudes, as suggested by Pálsson et al. (2009). We have updated the text accordingly.

TEXT REPLACED (Lines 196-198 in the originally submitted ms – Main text)
The absence of *B. saida* on the Morris Jesup Rise during MIS 2 could have been related to the isolation of localized refuges of *A. glacialis*, possibly in combination with unfavorable habitat conditions for *B. saida*.
TEXT REPLACEMENT (Lines 237-243 in the resubmitted ms – Main text):
The absence of *B. saida* from the Morris Jesup Rise and Lomonosov Ridge during MIS 2, along with the presence of only *A. glacialis* at the Morris Jesup Rise, aligns well with previous hypotheses about their climate-change adaptation strategies: *A. glacialis* likely persisted in a localized refuge, experiencing a population bottleneck and reduced genetic diversity, whereas *B. saida* appears to have retreated to lower latitudes, maintaining higher genetic variation through range expansion, as proposed by Pálsson et al. (2009)[56].

Reviewer #1, Line 512: The Methods state that otolith identification was based on Mecklenburg et al (2018), but that book only deals with fish identification, not otoliths. Which volumes were used in otolith identification?
AUTHOR RESPONSE: We recognize that we have not clearly referred to the identification literature, and we have now included the literature citations used to identify the *A. glacialis* and *B. saida* otoliths in this sentence. All these references (numbers 22, 73, 74, 75, 76) were already mentioned later in the Methods section, but it is appropriate to also include them already in this sentence for clarity.

TEXT REPLACED (Lines 510-516 in the originally submitted ms – Methods)
Identification of the otoliths from the sediment samples was based on previously published descriptions of otoliths from the 594 known fish species recorded in and around the Arctic Ocean[14]. Codfishes (Gadidae) in general possess relatively large and morphologically distinct otoliths, enhancing their preservation potential in marine sediments and facilitating their taxonomic identification[20,70,71,72]. Gadoid otoliths have a straight sulcus with a pseudobiostial

opening with clearly divided colliculi (also referred to as advanced homosulcoid), which are closed far from the edges of the otolith[71,73].

TEXT REPLACEMENT (Lines 344-353in the resubmitted ms – Methods):
The otoliths were identified by visually comparing their three-dimensional microstructures, including concavity, crenulation, and sulcus shape, under a microscope using otolith identification literature focused on the 594 known fish species recorded in and around the Arctic Ocean[14,22,78,79,80,81]. Out of the 256 otoliths in our sediment samples, 252 had sizes and characteristics similar to those of the Arctic endemic cods, the ice cod (*A. glacialis*) and polar cod (*B. saida*), which are most clearly described by Härkönen (1986)[81]. Gadidae in general possess relatively large and morphologically distinct otoliths, enhancing their preservation potential in marine sediments and facilitating their taxonomic identification[20,75,76,77]. Gadoid otoliths have a straight sulcus with a pseudobiostial opening with clearly divided colliculi (also referred to as advanced homosulcoid), which are closed far from the edges of the otolith[76,78].fragment

Reviewer #1, Line 573: I don't know what you mean by "elaborated"

AUTHOR RESPONSE: The word "elaborated" has been replaced by "processed", and the sentence was rephrased.

TEXT REPLACED (Lines 572-574 in the originally submitted ms – Methods)
To satisfy minimum weight requirements for $^{14}C$ analysis, including leaching, small (<3 mm) otoliths were elaborated whole and large otoliths were broken into fragments.

TEXT REPLACEMENT (Lines 414-416 in the resubmitted ms – Main text):
To meet the weight requirements for radiocarbon analysis, including leaching, small (<3 millimeters) otoliths were processed as whole, while only fragments were used from larger otoliths.

Reviewer #1, Line 586: Given that the outer leachate was much older than the inner otolith core, isn't it possible that the leaching process was asymmetric around the otolith, as is commonly observed? If so, some of the regions affected by diagenesis may not have been removed. Were any sequential leach samples investigated to test your assumption that the core was pristine? Contaminated cores might well explain the poor correspondence between sediment depth and radiocarbon age.

AUTHOR RESPONSE: The opposite is true: the outer leachate was much younger than the inner parts of the otoliths. We have rephrased this to avoid any confusion (see below). In line 112 (original ms), we state that otolith cores (after leaching) were verified for the absence of diagenesis (well-identified) using scanning electron microscopy (SEM). In line 575 (original ms). We further state that otoliths containing metal deposits or boreholes from worms were manually scraped to avoid contamination from diagenetic material (Line 574 original ms). Poor correlation between sediment depth and radiocarbon age is addressed in detail by the changes made to lines 117-125 and 165-170 (main text) of the originally submitted manuscript, see "Reviewer #1 - General comments" above for further explanation.

TEXT REPLACED (Lines 584-586 in the originally submitted ms – Methods)
The leaching treatment proved effective as the inner otolith parts were on average 8 ± 10 % (mean ± % standard deviation) older than their leachates, and more so for otoliths older than 20 ka, which were on average 20 ± 8 % (up to 37 %) older than their leachates.
TEXT REPLACEMENT (Lines 426-429 in the resubmitted ms – Methods):
The leaching treatment was effective, as the inner otolith parts were on average 8 ± 10% (mean ±% standard deviation) older than the outer parts, and even more so for the inner parts of the otoliths older than 20 ka, which were on average 20 ± 8% (up to 37%) older than the outer parts.

Reviewer #1, Line 832: The rationale for the otolith identification ordination is very strange. Why would you use PCA to analyze otoliths of unconfirmed species identity, rather than first analyze otoliths from fresh specimens of known identity? Why did you not use a discriminant analysis for classification, and then jackknife the classification to get an unbiased measure of classification accuracy? And most importantly, why did you include otolith area, perimeter and axis length in the PCA, when they are all highly correlated with fish length? You cannot put a covariate into a PCA; if you do, all you do is develop a classification tool that helps you tell large otoliths from small otoliths. The standard approach is to detrend the features that are correlated with fish length, then introduce the detrended variables into the PCA. As it stands, this PCA appears to be completely inappropriate and misleading.
AUTHOR RESPONSE: This matter is addressed in detail by the changes made to lines 86-90 and 92-96 (main text) and lines 534-345 (methods) of the originally submitted manuscript, see "Reviewer #1 - General comments" above for further explanation.

Reviewer #1, Extended Data Fig 7: The reconstructed fork lengths are more consistent with otolith dissolution, ecological size segregation or size-biased detection in the sediment core, than enhanced growth rate or maximum length. Both species typically mature at more than 18-20 cm, so it is plausible that some samples/dates are associated with periods of very small maturation and maximum lengths. But if there are otoliths present from large fish, then smaller individuals must have been present too. Extended Data Fig 7b shows that fish as small as 8 cm were detected in MIS 3, yet no fish less than 18 cm were identified in Fig 7a MIS 3. Since the absence of young fish is biologically impossible, the smaller otoliths were either not present in nature (rejecting any hypotheses about mortality) or, more likely, preferentially dissolved due to their higher surface area: volume ratio. With dissolution having been demonstrated in one era, who is to say that it didn't occur at slightly higher rates in the periods with missing otoliths?
AUTHOR RESPONSE: This matter is addressed in detail by the changes made to lines 200-204 (main text) of the originally submitted manuscript, see "Reviewer #1 - Specific comments" above for further explanation.

**Reviewer #2 – General comments**

I read the paper by Chawarski et al on the codfish presence in the Arctic in the last 50 ky with great interest, and indeed this is an excellent work and a very significant contribution in my opinion. I found that the results are indeed very significant and the paper is well-written. This

work I believe will form a significant contribution to the field, particularly because it demonstrates once more the great value of using fish otoliths obtained from sediment cores to understand the geohistorical evolution of fish populations. The methodology is sound and I only added very minor comments directly on the pdf. I very much look forward to seeing this paper published.
AUTHOR RESPONSE: We are pleased to hear that Reviewer #2 is positive about our manuscript, and we appreciate the review that helped us improve the manuscript. Below, we address the reviewer's specific comments.

**Reviewer #2 – Specific comments**
(the comments were inserted directly in manuscript)

Reviewer #2, Line 65: we added "and millions"
…, enabling the investigation of species composition of fish which lived thousands and millions of years ago.

Reviewer #2, Line 73: we added "(Fig. 1)"
… at five locations between 84 and 89 °N in the Arctic Polar Domain (Fig. 1).

Reviewer #2, Line 74: we added "s"
The otoliths were sampled from 889-1,537 m water depths on

Reviewer #2, Line 76: we added "from sieved samples (300 µm mesh)"
The otoliths were manually extracted from sieved samples (300 µm mesh) taken from the top 0–15 cm of sediment in box core samples covering an area of 0.25 square meters.

Reviewer #2, Line 97:
Reviewer comment: Since otoliths are formed throughout the lifetime of the fish, these radiocarbon ages would represent the times when the fishes "lived", not died.
To be most accurate, we chose to use the term 'died' because the radiocarbon "clock" starts ticking when $^{14}C$ stops being replenished, i.e. upon death. We recommend keeping the text as is.

Reviewer #2, Line 76: we deleted "usually"
Otoliths ~~usually~~ consist of precipitated calcium carbonate in the form of aragonite or vaterite, which are more prone to dissolution than calcite[24].

Reviewer #2, Lines 112-114:
Reviewer comment (since we leached away the outer part of the otoliths): The radiocarbon age would be closer actually to the earlier part of the life of the fish than the time of its death, which is represented by the outermost surface of the otolith, which has either been dissolved due to diagenesis or due to the leaching applied here. Of course, this does not affect he results drawn in this paper, but this point should be clarified anyway.
AUTHOR RESPONSE: The uncertainty in radiocarbon dating is much greater than the lifespan of the individual fish. Thus, the radiocarbon age cannot be linked to any particular stage of the fish's life, as these fish species have a lifespan of up to 6-12 years.

Reviewer #2, Line 538 (Methods):
Reviewer comment: How were the morphological features decided? Can these be related to the features used in the AFORO database? http://aforo.cmima.csic.es/
AUTHOR RESPONSE: We used photographic images of our otoliths to derive two-dimensional features. After checking for covariance among features, these were the remaining two-dimensional features we could compute using Fiji software. We are grateful to the reviewer for sharing this (to us) previously unknown database. Unfortunately, *Arctogadus glacialis* is not available in this database, and the only reference found for *Boreogadus saida* was also cited and used in our morphological identification (Campana et al. 2004).

Reviewer #2, Line 538 (Methods):
Reviewer comment: Does "Area" refer to the inner face of the otoliths?
AUTHOR RESPONSE: Yes, but as these otoliths lack concavity, we assume the two-dimensional area of the otolith is identical on both sides.

**Reviewer #3 – General comments**

Thank you for the opportunity to review this paper, I read it with great interest. Chawarski et al. present a very exciting analysis of long-term distribution of cod fishes using otolith morphometrics. The approach is ambitious, thorough and yields important insights into the past and potential future distribution of cold-adapted fish. This paper offers key knowledge for current management efforts under the rising climate change. I only have some minor comments/suggestions that I would like the authors to consider.
AUTHOR RESPONSE: We are pleased to hear that Reviewer #3 is positive about our manuscript, and we appreciate the review that helped us improve the manuscript. Below, we address the reviewer's specific comments.

**Reviewer #3 – Specific comments**

Reviewer #3, Line 60:
Be more precise in what are "these adaptations" for both fish species.

AUTHOR RESPONSE: This sentence has also changed according to the comment of Reviewer #1 (see above and below).

TEXT REPLACED (Lines 57-62 in the originally submitted ms – Main text):
These two Arctic codfishes constitute important links between the lower and higher trophic levels of the Arctic food web[12,17] and are highly adapted to cold ocean temperatures and under-ice habitats. and are highly adapted to cold ocean temperatures and under-ice habitats. Whether these adaptations enabled them to endure the harshest environmental conditions in the Arctic Polar Domain during the last ice age, is unknown.
TEXT REPLACEMENT (Lines 56-61 in the resubmitted ms – Main text):
Together, these two Arctic gadoids form key links between the lower and higher trophic levels of the contemporary Arctic food web[12,17]. However, despite their cold adaptations, such as antifreeze glycoproteins that enable them to thrive in water temperatures below 0 ºC, it remains an open question whether these fish could have survived the extreme conditions of the last ice age, when ecosystem productivity in the Arctic Polar Domain was likely much lower than it is today.

Reviewer #3, Line 63: Is it possible to find any other fish bone in the sediment core? Why not using those too? Consider adding a very small statement of why exactly otoliths are preferred to investigate past environmental conditions and fish movement.
AUTHOR RESPONSE: Otoliths are composed of aragonite, whereas bones and scales are composed of collagen, hydroxyapatite (a calcium phosphate mineral), and organic material that is more readily broken down by microbes, worms, and chemical processes. The mineral rich microcrystalline structure of otoliths also slows down dissolution, compared to other bony structures. These processes might be the reason for the absence of any bones or scales at our sampling sites. We have adapted the sentence as follows:

TEXT REPLACED (Lines 63-64 in the originally submitted ms):
"Ear stones" (otoliths) from the heads of bony fishes, which are commonly preserved in sea-floor sediments represent …
TEXT REPLACEMENT (Lines 62-64 in the resubmitted ms)
Otoliths (ear stones) from the heads of bony fishes are more resistant to degradation than other skeletal remains because of their chemical composition, and therefore are readily preserved in seafloor sediments.

Reviewer #3, Figure 3: $^{14}C$ dating samples can always be problematic, but could the broad range in the age of some samples be related with the ΔR values used for the marine reservoir correction? You used the same value for both fishes, but shouldn't we use different values given the sampling locations and/or the feeding grounds/diet specificity for each fish? Could the selected ΔR value potentially affect the distributional range (in years) given in Figure 3?
AUTHOR RESPONSE: This is an interesting point, and the reviewer correctly notes that, technically, differences in ΔR may be necessary for different species. Unfortunately, there is no available data to constrain this. However, since the fish species generally occupy similar habitat depths, the difference is likely minor. Additionally, differences in ΔR for the Holocene period

typically span up to hundreds of years, leading us to believe that even significant differences in ΔR would not impact the main conclusions (i.e., we consider it unlikely or impossible for this to alter the observed distribution of Holocene-aged versus MIS 3 otoliths).

Reviewer #3, Line 143: misspelling of "Further".
AUTHOR RESPONSE: We have corrected the spelling of "Further".

Reviewer #3, Line 191: I found the presence/absence of polar cod very interesting, especially because there seems to be a trend in "higher presence of polar cod" with "low presence of ice cod". Do these two fish species interact/compete? From your data here (Line 191), it seems likely to be looking to "isolated populations" of ice cod as mentioned in the text. But what about (predator/prey) competition interactions specially during the MIS3 and MIS1? Ecological dynamics will generally direct distributional patterns of different species. It is not clear from your text whether one species outcompeted the other under certain conditions.
AUTHOR RESPONSE: Ecological dynamics across these different periods are of great interest to our team. We discuss some changes in the ecological landscape at lines 188-189, 203-204, and in the paragraph following line 234 (see the original manuscript for line numbers). Our current understanding of modern populations suggests that, in some geographic regions, they are sympatric with highly overlapping life histories. However, we can only speculate on how they may have interacted or differed over the timescale of our study period. Therefore, we are cautious about providing additional details without supporting evidence. Nonetheless, genetic evidence offers an essential clue to their potential speciation mechanisms, and we have updated the sentence at lines 196-198 to emphasize this point. This sentence has also been revised in accordance with the comments of Reviewer #1 (see above and below).

TEXT REPLACED (Lines 196-198 in the originally submitted ms – Main text)
The absence of *B. saida* on the Morris Jesup Rise during MIS 2 could have been related to the isolation of localized refuges of *A. glacialis*, possibly in combination with unfavorable habitat conditions for *B. saida*.
TEXT REPLACEMENT (Lines 237-243 in the resubmitted ms – Main text):
The absence of *B. saida* from the Morris Jesup Rise and Lomonosov Ridge during MIS 2, along with the presence of only *A. glacialis* at the Morris Jesup Rise, aligns well with previous hypotheses about their climate-change adaptation strategies: *A. glacialis* likely persisted in a localized refuge, experiencing a population bottleneck and reduced genetic diversity, whereas *B. saida* appears to have retreated to lower latitudes, maintaining higher genetic variation through range expansion, as proposed by Pálsson et al. (2009)[56].

Reviewer #3, Line 521: Spell out the name of the Greenland cod (*Gadus ogac*).
AUTHOR RESPONSE: We have now spelled out the full name *Gadus ogac*.

250603 – Paper submitted to Nature Communications
250719 – First review
250927 – First resubmission
260122 – Second review
260401 – Second resubmission

## REVIEWER COMMENTS SECOND REVIEW 260122

## AUTHOR RESPONSES IN BLUE 260401

### Reviewer #2 (Remarks to the Author):

I appreciate the efforts made by the authors to address my previous comments on the manuscript. However, I still have some reservations, particularly about the identification of the otoliths.

AUTHOR RESPONSE: In the revised manuscript, we have carefully addressed the reservations of Reviewer #2 about their remaining Comments 1 and 2 below. We are very grateful to Reviewer #2 for helping us clarify our species identification procedure and for helpful comments on the manuscript.

Reviewer #2 Comment 1:

In response to my previous comment that "Since otoliths are formed throughout the lifetime of the fish, these radiocarbon ages would represent the times when the fishes "lived", not died." the authors responded that "To be most accurate, we chose to use the term 'died' because the radiocarbon "clock" starts ticking when 14C stops being replenished, i.e. upon death. We recommend keeping the text as is." This is not correct. What you analyzed was the carbon in the aragonite of the otoliths, which is deposited throughout the lifetime of the fish and not replenished. Every day, a new increment of aragonite crystallites is deposited around the previous ones.

AUTHOR RESPONSE: The sentence "Since otoliths are formed throughout the lifetime of the fish, these radiocarbon ages would represent the times when the fishes "lived", not died." was already removed from the manuscript during the previous revision, but we overlooked this detail in our previous Author Response. We recognize that this drawback was mentioned by both Reviewers #1 and #2, and we agree with the sentiment. The aforementioned sentence was removed to reduce confusion. Several sentences were merged into one, and it now reads (Lines 89-92): Radiocarbon dating of a subset of 79 otoliths showed that the calibrated ages ranged from 1,3 ± 0.2 ka BP (mean ± standard deviation) to 47,8 ± 2,2 ka BP (Supplementary Data 4). Thirty-seven otoliths were dated to 11.3–1.3 ka BP (Holocene), 13 to 20.3–18.6 ka BP (LGM), and 29 to 47.8–29.1 ka BP (last ice age before the LGM).

2) In response to my comment "How were the morphological features decided?", the authors responded that "We used photographic images of our otoliths to derive two-dimensional features. After checking for covariance among features, these were the remaining two-dimensional features we could compute using Fiji software." This does not answer my question. There is very abundant literature on this, such as the pivotal works of Nolf (1985, Otolith Piscium; and 2013) or several works by Werner Schwarzhans. I am surprised that the authors did not consider these at all. Furthermore, otoliths that end up in sediments go through significant taphonomic process, particularly some erosion of the outer surface, but also occasional breaking, and other modifications (see Agiadi et al. 2022; Paleobiology). Therefore, it is vital that the features used to identify them are clearly defined, even more so than for otoliths extracted from live-collected fish. This reservation is further reinforced by the authors' answer to my next comment "Does “Area” refer to the inner face of the otoliths?", to which they replied "Yes, but as these otoliths lack concavity, we assume the two-dimensional area of the otolith is identical on both sides." Absolutely not! Otoliths (of all species) do not have identical inner and outer faces! The inner face is the one characterized by the sulcus acusticus, which is a depending where the sensory macula of the inner ear are attached. It has a clear functional role, and it the most characteristic identifying feature of otoliths. Without properly addressing the issue of otolith identification, I cannot recommend accepting the manuscript.

**Nolf, D. & Steurbaut, E., 1989a. Evidence from otoliths for establishing relationships between gadiforms and other groups. In: Cohen, D.M. (ed.), Papers on the systematics of gadiform fishes. Science Series, Natural History Museum of Los Angeles County, 32: 37–45.

**Nolf, D. & Steurbaut, E., 1989b. Importance and restrictions of the otolith-based fossil record of gadiform and ophidiiform fishes. In: Cohen, D.M. (ed.), Papers on the systematics of gadiform fishes. Science Series, Natural History Museum of Los Angeles County, 32: 47–58.

AUTHOR RESPONSE (NOTE: the same as to Comment 1 of Reviewer #4):

We understand the reviewer's reservations about the identification process and believe these reservations stem primarily from unclear paper structure and wording on our part. We have substantially modified the text by completely rephrasing the key paragraph in the METHODS section (Lines 315-337) called “Identification of gadoid fish species”, (now also including a reference to Nolf & Steurbaut (1989)), in which the identification procedure is explained step by step, and referring to Supplementary Figs 1 and 3, and their legends, which show photographs and further details on the morphological features of the two gadoids that were used for species identification. We have also modified and reduced the text in the section called “Two-dimensional feature analysis” (Lines 339-349). Two-dimensional features, such as “area” (square millimeters) are derived from photographs and are not related to the surface area of a given side of the otolith. **We emphasize that this two-dimensional feature analysis step was not used in the species identification processes and is not essential to the results and conclusion of this study.** Here we describe – in a comprehensive way – to demonstrate the steps we have taken to visually identify the otoliths, the literature used, and the secondary

purpose of the 2-dimensional morphometrics that are summarized in Lines 315-349 of the manuscript

**Step 1**: We went through the list of species that are known in the Arctic Oceans:
Mecklenburg, C. W. et al. Marine fishes of the Arctic region, Volume I. CAFF Monitoring Report 18, 1–454 (2018)
This is a critical step as there is little to no knowledge of the fish species composition in the Central Arctic Ocean where our sampling stations were, especially on pelagic species. Also, recently we are increasingly finding more boreal species such as Atlantic cod, walleye pollock, and *Benthosema glaciale*. Thus, knowing which species occur in and around the Arctic gave us a first starting point on which otolith morphology we have to be aware of.

**Step 2**: Then we did a literature review of manuscripts that contained otolith identification features and descriptions of the species we expected to encounter in our sediment samples:

- Frost, K. J. Descriptive key to the otoliths of gadid fishes of the Bering, Chukchi, and Beaufort Seas. Arctic 34, 55–59 (1981)
- Svetocheva, O., Stasenkova, N. & Fooks, G. Guide to the otoliths of the bony fishes of the White Sea. IMR/PINRO Joint Report Series 3, 1–46 (2007)
- Boulva, J. Morphometries of three sympatric Arctic codfishes of the genera Arctogadus and Gadus. Journal of the Fisheries Research Board of Canada 29, 243–249 (1972)
- Lidster, W. W., Lilly, G. R. & Dawe, E. G. Otoliths of Arctic Cod (*Boreogadus saida*), small Atlantic Cod (Gadus morhua), and three other fish species from Newfoundland waters: Description and relationship of body length to otolith lengths. Journal of Northwest Atlantic Fishery Science 16, 33–40 (1994).
- Campana, S.E. Photographic Atlas of Fish Otoliths of the Northwest Atlantic Ocean. NRC Research Press, Ottawa, Canada. 284 pp. (2004)
- Nolf, D. & Steurbaut, E., 1989a. Evidence from otoliths for establishing relationships between gadiforms and other groups. In: Cohen, D.M. (ed.), Papers on the systematics of gadiform fishes. Science Series, Natural History Museum of Los Angeles County, 32: 37–45.

It is noteworthy that *Arctogadus glacialis* is not included in the open database AFORO for otolith identification. We contacted several Arctic fisheries agencies and were unable to find any that held modern otolith collections of *Arctogadus glacialis*. Otolith photographs in Härkönen 1986 are the clearest depictions of the ice cod otolith, which are supported by the drawings the following two manuscripts:

- Frost, K. J. Descriptive key to the otoliths of gadid fishes of the Bering, Chukchi, and Beaufort Seas. Arctic 34, 55–59 (1981)
- Nolf, D. & Steurbaut, E., 1989a. Evidence from otoliths for establishing relationships between gadiforms and other groups. In: Cohen, D.M. (ed.), Papers on the systematics of gadiform fishes. Science Series, Natural History Museum of Los Angeles County, 32: 37–45.

**Step 3**: After this literature search, combined with our prior expertise in working with fish otoliths from modern surveys, we were confident that the morphology of our sediment otoliths were of Gadoids, and that the features were related to the two native gadoids *Boreogadus saida* and *Arctogadus glacialis*. Furthermore, we were able to easily compare these sediment otoliths with modern collections of *Boreogadus saida* otoliths, which are extensively studied at one of our collaborating institutes (Alfred Wegener Institute).
In Supplementary Figure 1 we summarised our findings on how the otoliths differ from the sub-arctic gadoids:

- More concave lateral surface of subarctic gadoids compared the native Arctic gadoids
- Collicula more raised and defined in the native gadoids and present in juvenile otoliths
- Crenulations more pronounced in otoliths of sub-arctic gadoid juveniles
- Lateral surface being smoother in the Arctic native gadoids, compared to the extensive lobing in sub-arctic gadoids

The images of the actual sediment otoliths show the differences between the two native Arctic gadoids:

- Elongated shape of the *Boreogadus saida* otolith compared to the wider elliptoid shape of the Arctogadus. Regarding these differences: Nolf et al. (1989) described as *Arctogadus* otoliths as “rather high” and Frost et al. (1981) described *Arctogadus* otoliths as “Otolith width usually 50% or more of otolith length” compared to *Boreogadus* as “Otolith width usually less than 47% of otolith length”
- Adult *Boreogadus saida* otoliths contain more crenulations around the entire otolith margin than *Arctogadus glacialis*.

These visual features allowed us to distinguish our sediment otoliths from other Arctic fish species and related gadoids, and distinguish between these two species, despite naturally occurring variations among individuals and sizes.

**Step 4**: Due to the varying state of preservation of otoliths (i.e. diagenesis, damage, erosion), we found it prudent to add a confidence score to our identifications. We observed smoothing of features along otolith margins, boreholes presumably caused by worms, degradation where otoliths become brittle, and losses of otolith parts due to fragmentation. Our confidence scores and examples for each score are provide Supplementary Figure 3, and in our included datasheet you will find these scores for each otolith. Most otoliths were in suitable conditions, such that shape and the features described in step 3 for the Arctic native gadoids were consistently identifiable, especially if differences in the Aspect Ratio (width/length) are taken into account. We have also included new SEM/EDS results (Supplementary Fig. 7) showing that there are no significant differences between the structure of sediment otoliths and recent otoliths (from freshly caught fish), indicating that the organic surface coating present in recent otoliths is absent in our sediment otoliths.

**Step 5**: As an extra step, which was never used to identify species, we opted to conduct an unbiased analysis of the 2D morphometric features based on the otolith pictures. This allowed us to compare our identifications clustered across a four-dimensional feature space and further examine how our IDs, otolith sizes, confidence scores compared to these groupings (Supplementary Figure 5). While we find two-dimensional features insufficient for identification alone, several prior papers (as noted in Step 3) mentioned difference in the aspect ratio can be a key differentiator between *A. glacialis* and *B. saida*. This aligned with results of a binomial logistic regression of aspect ratio (Supplementary Figure 6), but we caution that the strength of this feature in differentiation is limited to larger otoliths only (also demonstrated in Supplementary Figure 5). We included this analysis because we believe that it will provide useful insight for future researchers who may encounter gadoids otoliths in Arctic sediments.

**To reiterate, we did not use Step 5 to identify the species.** The actual identifications were done visually and are primarily based on the finer morphological features described above that are displayed on both sides of the otolith (medial and ventral surface). These finer 3D features (sulcus, collicula) mostly distinguish the native Arctic gadoids from the sub-arctic gadoids and rely on visual evaluation. The larger 2D features (such as area, which refers to the surface area in square millimeters) can be captured in a broad analysis. This 2D 'area' feature is thus the same at each side of the otolith and is not related to the finer 3D structures such as the sulcus acousticus.

## Reviewer #3 (Remarks to the Author):

Thank you to the authors for the thoughtful evaluation of the revisions. All my comments have been successfully addressed. I have no additional comments/feedback.

AUTHOR RESPONSE: We are grateful to Reviewer #3 for helping us improve the manuscript.

## Reviewer #4 (Remarks to the Author):

General comments:
This manuscript is interesting and presents a rare and potentially high-impact dataset. The approach is original and, in my view, aligns well with current efforts to push sedimentary fish archives (otolith-based records) towards broader palaeoecological and climate-relevance questions. My suggestions are primarily intended to 1. streamline and refocus the Results/Discussion, and 2. improve the otolith-specific accuracy and citations, as several statements and references currently read as coming from a non-specialist otolith perspective.

AUTHOR RESPONSE: We thank Reviewer #4 for helping us improve the manuscript, including the sections on otolith structure and nomenclature.

General and major suggestions:

1) On the morphometrics and identification: I appreciate the authors' effort to make identifications more objective using morphometric analyses, especially given that the authors are not primarily otolith specialists. However, the morphometrics currently occupy a disproportionate fraction of the manuscript (it is important, but currently over-emphasised in the main text). In my opinion, the otoliths of the two species are readily distinguishable, and the highlighted feature (aspect ratio) is visually obvious. Based on the figures and descriptions provided, I consider the identifications to be credible. Therefore, I do not think it is necessary to present the morphometric analyses at such length in the main manuscript. Related to your methodology: if the main objective is to separate two species, then a supervised discriminant framework (e.g., LDA or another classifier with cross-validation) is generally more appropriate than PCA, which is exploratory/unsupervised. This aligns with Reviewer #1's view. My reading is that you selected PCA primarily to visualize and corroborate an a priori observed feature (i.e., aspect ratio) rather than to serve as a formal classification tool. If so, PCA can be retained, but the manuscript should explicitly clarify this intent and avoid presenting PCA as an "objective validation" of identifications. That said, one important nuance about what the PCA is being used to claim is needed. If feasible, I recommend adding a supervised classification analysis (or at least reporting classification accuracy with an appropriate validation scheme) as the primary quantitative support for species separation, with PCA presented as supplementary, exploratory context.

AUTHOR RESPONSE (the same as to Comment 2 of Reviewer #2, see above for more information):

We appreciate that Reviewer #4 identified the distinction between supervised discriminant analysis and visualization of a priori observed features and identifications. Our intent was indeed to use PCA for the latter. Future studies would certainly benefit from an expanded database of validated otoliths of both species that could be use in discriminant analysis. Unfortunately, supervised classification using validated otoliths was unavailable during our study and therefore unfeasible. We emphasize that this step was not used in the species identification processes and is not essential to the results and conclusion of this study. Nevertheless, we believe that this information will be useful for future researchers who encounter otoliths in deep Arctic sediments. We have reduced the text and emphasis on the this analysis which is now briefly described in the METHODS section called "Two dimensional feature analysis (Lines 339-349)."

We highly appreciate the Reviewer's advice about correctly describing the process of species identification. In the RESULTS AND CONCLUSIONS section (Lines 82-88), we now only refer to the numbers of otoliths identified to species and Fig. 2, including its legend. There is a completely rephrased paragraph in the METHODS section (Lines 315-337) called "Identification of gadoid fish species", now also including Nolf & Steurbaut (1989) (Reference 69), in which the identification procedure is explained step by step, and referring to Supplementary Figs 1-3, and

their legends, which show microscopic micrographs and further details on the morphological features of the two gadoids that were used for species identification.

2) For the chronology and sediment mixing, I think the "two time-layer" framing is reasonable, but this has to be kept clear and more concise. I appreciate that the authors have tried their best to resolve the age issue and possible sediment mixing. In our experience, minor to moderate mixing can indeed occur (see also Lin et al. 2019 for a reefal example and discussion). I also think the authors have responded reasonably by clarifying that the material can be broadly divided into two time-layers following Reviewer #1's view, which I also think is a critical clarification. My suggestion is mainly about presentation: keep this interpretation front-and-centre and ensure the manuscript does not inadvertently drift back into an implied continuous age–depth narrative. A short schematic could help (even in the Supplement).

AUTHOR RESPONSE: We are pleased to hear that the revised clarifications regarding the "two-layer" framework were well received. We have carefully rephrased the part about mixing in the RESULTS AND DISCUSSION SECTION (Lines 111-130), where **we emphasize that our interpretations stem from direct dating of the otoliths, without constructing age-depth models for the cores**. Instead of a schematic, we added the following explanation to the legend of Fig. 3: "Otoliths younger than 12,000 years (Holocene) were generally found in the Holocene layer, and otoliths older than 35,000 years in the lower 5–15 cm glacial layers. Sediment mixing (bioturbation) likely occurred within each layer." Since the absence of LGM otoliths on the Lomonosov Ridge is evident from direct dating of the otoliths (Fig. 4), and does not stem from any stratigraphic interval lacking in otoliths (Fig. 3), the paucity in otolith deposition during this time (on the Lomonosov Ridge) is most likely related to changes in environmental conditions. Furthermore, considerable dissolution of aragonite is an unlikely cause for the paucity in otolith deposition during the LGM (on the Lomonosov Ridge), since the sediment horizons with otoliths are rich in calcareous microfossil assemblages, including occurrences of (aragonitic) pteropod shells, e.g., found at Stations 26 and 53. This is mentioned in the RESULTS and DISCUSSION section (Line 105), and **an example of this is seen in the following photograph, which could be included as a Supplementary Figure upon request of the Editor.**

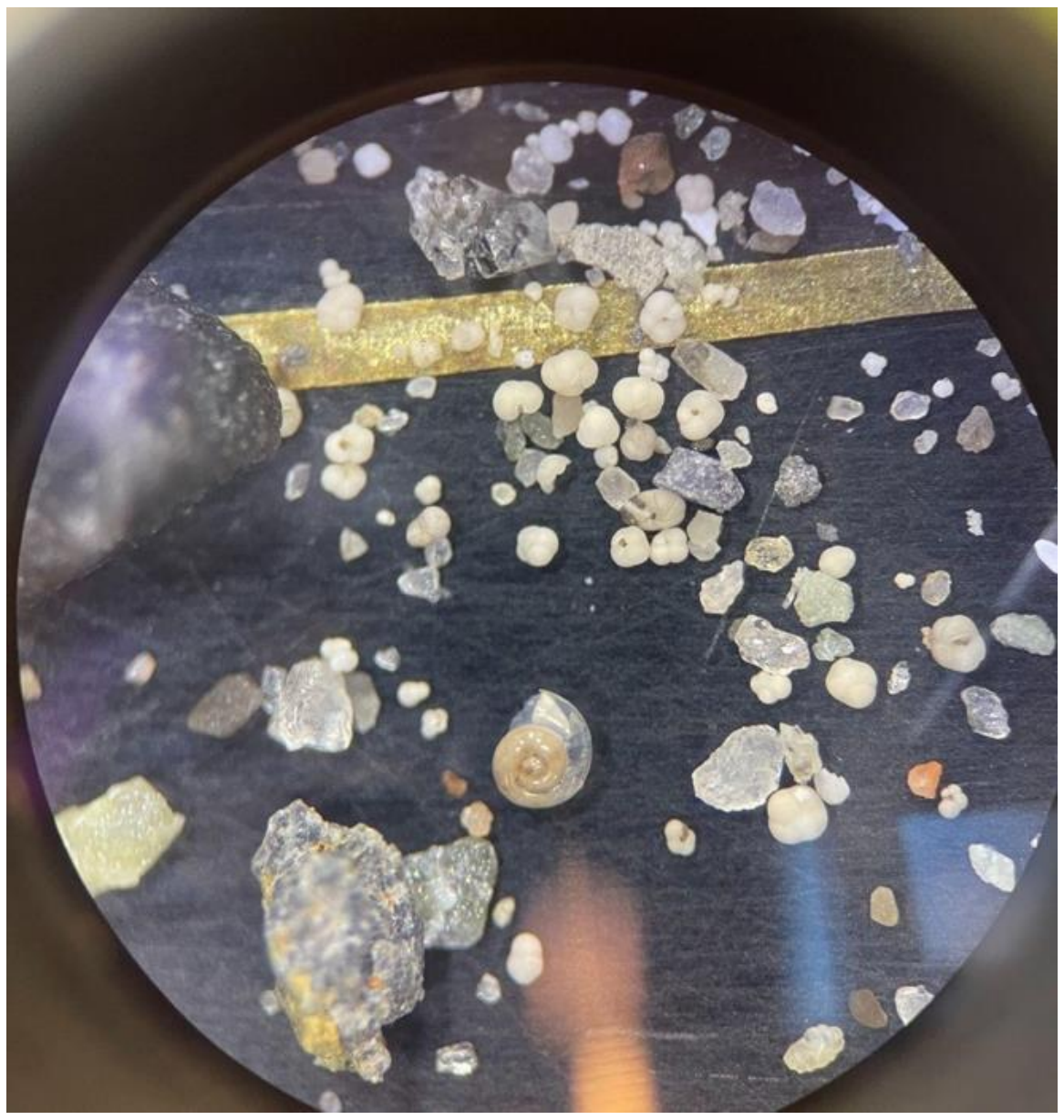

**Microscopic view of a sediment sample from Station 53, stratum 13-14 cm, showing a pteropod (red arrow) and foraminifers (blue arrows).** The co-occurrence of our MIS3-aged otoliths with aragonitic pteropods and calcareous microfossils (foraminifers) and (aragonitic) pteropod shells makes aragonite dissolution bias of our otoliths unlikely.

3) At the moment, parts of the results read unfocused relative to the key findings, so that it reads methodologically heavier than necessary in the main narrative. The manuscript would benefit from sharper writing and a clearer hierarchy of what is central versus ancillary. Especially the sections titled “Otoliths from Arctic endemic cod species” and “Gadoid otoliths and climate over the past 50,000 years” are currently distracting compared with the most important findings. For example, the PCA results read as diffusing the narrative; it is written partly as “validation,” partly as “exploration,” partly as “methods defense.” That blend tends to bloat text. In Lines 123–140 include marginal details that could be moved to the Supplementary Information without loss of scientific value. I strongly encourage a restructure so the main text concentrates on the most important biological/climate conclusions, with methodological depth placed in the Supplement.

AUTHOR RESPONSE: We have revised, refocused and reconstructed the RESULTS AND DISCUSSION section so that the main results, as summarized in the Abstract (also revised), are central. As already explained under Comment 1, the morphometric analyses (Supplementary Figs. 4-6) have been removed from RESULTS AND DISCUSSION as they have no impact on the results of the study.

4) Dissolution problem. I agree that dissolution of otoliths, particularly affecting smaller specimens is not likely to be the primary driver here, given repeated discoveries of small otoliths. Previous actuo-palaeontological works also show that dissolved otoliths can typically be distinguished from intact specimens (e.g., Schwarzhans 2013; Lin et al. 2016, 2017). However, I suggest adding the ACD/lysocline context for the region (and explicitly relating your station depths to it) would make the depth-related carbonate dissolution argument much clearer.

**Lin, C.-H. et al., 2016. Fish otolith assemblages from Recent NE Atlantic sea bottoms: A

comparative study of palaeoecology. Palaeogeography, Palaeoclimatology, Palaeoecology 446: 98–107.
**Lin, C.-H. et al., 2017. Fish otoliths in superficial sediments of the Mediterranean Sea. Palaeogeography, Palaeoclimatology, Palaeoecology 471: 134–143.
**Schwarzhans, W., 2013. Otoliths from dredges in the Gulf of Guinea and off the Azores – an actuo-paleontological case study. Palaeo-Ichthyologica 13, 7–40.

AUTHOR RESPONSE: We have expanded the discussion about dissolution and the aragonite lysocline in the CAO in the RESULTS AND DISCUSSION section (Lines 92-106), where we further argue that an aragonite dissolution bias is unlikely in our otolith material from the Arctic Ocean. See also Comment 2. We have included the reference Lin et al. (2016) (Reference 22).

Additional comments on otolith-specific accuracy
5) Line ~62: Please revise the statement about otolith resistance and chemical composition. Your revised text is incorrect. The statement that otoliths are more resistant to degradation than skeletal elements because of their chemical composition is not correct. Otoliths are aragonitic and can be vulnerable under lower pH settings, whereas phosphatic remains (teeth, vertebrae) are often more robustly preserved. In many oceanic cores, otoliths may be absent while vertebrae/teeth are abundant (see e.g., Salvatteci et al. 2022; Sibert et al.).
The fundamental advantage of otoliths is instead their taxonomic value and often high representation (when preserved), rather than a blanket claim of superior resistance. In my view, otoliths, teeth, and other fish remains are complementary proxies, each with different preservation biases and strengths, rather than one being "better" than the others.

AUTHOR RESPONSE: We agree with the Reviewer's arguments that both otoliths and phosphatic remains are readily preserved in sediments and that there may be reasons for the absence of either. We have rephrased the sentence in RESULTS AND DISCUSSION (Lines 76-77) as follows: "Other fish remains, such as teeth or vertebrae, were not immediately apparent in the sieved sediments."

6) Following the above, citations around deep-sea otolith applications need correction
Jones & Checkley (2019) is appropriate. However: Salvatteci et al. (2022) did not use otoliths (as cited here); and the setting and focus of Agiadi et al. (as currently cited) do not align with the deep-sea sediment-core otolith context you are invoking.
Because otolith studies from deep-sea cores remain relatively rare (and novel), the literature selection here matters. More appropriate examples to cite in this context include Lin et al. (2023) and Pallacks et al. (2025) (both explicitly demonstrate downcore otolith records used for palaeoecological inference).

**Pallacks, et al., 2025. Ocean deoxygenation linked to ancient mesopelagic fish decline. Communications Earth & Environment 6: 596.

**Lin et al., 2023. Ocean temperature drove changes in the mesopelagic fish community at the edge of the Pacific Warm Pool over the past 460,000 years. Science Advances 9: 27, eadf0656.

AUTHOR RESPONSE: We agree with the Reviewer that the previous references were not ideal, and we have now referred to Lin et al. (2023) and Pallacks et al. (2025) (References 17 and 18) instead of the now deleted references Jones & Checkley (219), Salvatteci et al. 2022) and Agiadi et al. (2023).

7) Lines ~96, 102, 111 and Supplementary Fig. 1: Please revise standard otolith terminology: Use otolith length (OL) rather than "X mm long"; Use length and height (not "length and width") for primary dimensions, and ensure consistency throughout (including Supplementary figures).

AUTHOR RESPONSE: We have updated the terminology throughout the manuscript following the Reviewer's comments, including in Supplementary Fig. 1 (now "height" instead of "width").

8) Line ~137: The acid-leaching approach and use of inner material for radiocarbon dating is reasonable. As an optional improvement, micro-Raman spectroscopy can provide a rapid check of mineralogy (aragonite vs alteration products) to support your diagenesis discussion.

AUTHOR RESPONSE: We have studied our otoliths by SEM/EDS and observed no authigenic overgrowth (METHODS, Lines 375, 395). We have also included new SEM/EDS results (Supplementary Fig. 7) showing that there are no significant differences between the structure of sediment otoliths and recent otoliths (from freshly caught fish), indicating that the organic surface coating present in recent otoliths is absent in our sediment otoliths. Unfortunately, we do not have access to micro-Raman spectroscopy.

9) Line ~350: The illustrations in Nolf & Steurbaut (1989c; fig. 8) include otoliths of both species and support your identifications. I recommend explicitly citing this source (and, if feasible, using it as a comparative reference figure in the Supplement).

AUTHOR RESPONSE: We have included the reference Nolf & Steurbaut (1989) (Reference 69, Lines 328, 599, 773). **Upon request of the Editor, we are prepared to include the following line drawings from this publication as a Supplementary Figure, but we do not find this really necessary.**

Published Data Figure Redacted

10) Line ~352: revise awkward citations, these are non-gadid sources. Agiadi et al. (your ref. #20) and Schwarzhans (#76) do not deal with gadids in the way needed here. The most relevant citations for gadid otolith morphology and comparative framework should be Nolf & Steurbaut (1989a, b, c).

**Nolf, D. & Steurbaut, E., 1989a. Evidence from otoliths for establishing relationships between gadiforms and other groups. In: Cohen, D.M. (ed.), Papers on the systematics of gadiform fishes. Science Series, Natural History Museum of Los Angeles County, 32: 37–45.
**Nolf, D. & Steurbaut, E., 1989b. Importance and restrictions of the otolith-based fossil record of gadiform and ophidiiform fishes. In: Cohen, D.M. (ed.), Papers on the systematics of gadiform fishes. Science Series, Natural History Museum of Los Angeles County, 32: 47–58.
**Nolf, D. & Steurbaut, E., 1989c. Evidence from otoliths for establishing relationships within gadiforms. In: Cohen, D.M. (ed.), Papers on the systematics of gadiform fishes. Science Series, Natural History Museum of Los Angeles County, 32: 89–111.

AUTHOR RESPONSE: We have included the reference Nolf & Steurbaut (1989) (Reference 69, Lines 328, 599, 773) and deleted Agiadi et al. (2023) and Schwarzhans (1978).

11) Line ~353 and Supplementary Fig. 1: plural of "colliculum"

The plural of colliculum should be collicula (not “colliculi”). Please revise consistently in text and figures.

AUTHOR RESPONSE: We have corrected the spelling of “collicula” throughout the manuscript following the Reviewer's comment, including in Supplementary Fig. 1.

12) Figure 1 legend. In the Figure 1 legend, “Stations 26 and 38” are listed on Lomonosov Ridge and “Stations 38, 50, and 53” on Morris Jesup Rise. The second “38” appears to be a typo

AUTHOR RESPONSE: This was indeed a typo. It has now been corrected to: “Stations 26 and 38 are located on the western Lomonosov Ridge (Central Arctic Ocean), while Stations 48, 50, and 53 are on the Morris Jesup Rise, …” (Lines 678-680).

13) Supplementary Figs. 1 & 3. The otolith face orientation needs improvement; it appears the authors misunderstood the basic otolith orientation. The face bearing the sulcus is the medial/inner face, and the opposite is the lateral/outer face. Please revise these figures and thoroughly check the text.

AUTHOR RESPONSE: The face orientations were wrong in both figures. We have corrected them in Supplementary Figs 1 and 2 (note that the latter is the old Supplementary Figure 3 with *Paraliparis* otoliths).
